# Unmasking the UV Photobleaching of β-Diketonate [Eu(BTFA)_4_]^−^ Complexes as an Energy-Driven Photoreduction Process

**DOI:** 10.1021/acs.inorgchem.4c05014

**Published:** 2025-02-18

**Authors:** Lizandra L. L. S. Melo, Gerson P. Castro, Marcelo Navarro, Simone M. C. Gonçalves, Alfredo M. Simas

**Affiliations:** Departamento de Química Fundamental, CCEN, Universidade Federal de Pernambuco, Recife, Pernambuco 50670-901, Brazil

## Abstract

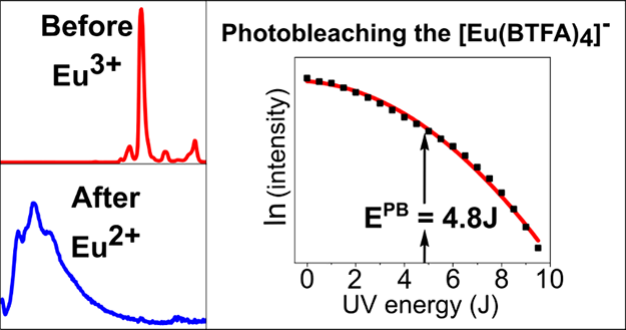

We elucidate the nature of the gradual attenuation of luminescence under sustained ultraviolet (UV) radiation exposure in europium [Eu(BTFA)_4_]^−^ complexes, revealing that it originates from a nonlinear energy-driven process, wherein Eu(III) is irreversibly reduced to Eu(II). This photoreduction was confirmed via the electrochemical techniques cyclic voltammetry and chronoamperometry, which, coupled with luminescence experiments, provided direct evidence of the Eu^3+^/Eu^2+^ reduction and the disappearance of Eu(III) after UV exposure. Indeed, extended UV exposure induces a gradual decrease in Eu(III) luminescence, paired with an increased intensity of Eu(II) luminescence, upon direct excitation of the metal ion. To provide an adequate description of this process, we advanced a photochemical law in the energy domain (rather than the time domain) and introduced the concept of photobleaching energy to facilitate comparisons of photostability among various complexes in different solvents. The nonlinearities detected in the energy domain suggest an underlying multiphoton process. As a consequence, we uncovered a trade-off between photostability and luminescence: enhancing one implies diminishing the other. Specifically, the proximity between the complex and cation in nonpolar solvents induces asymmetry in the complex, enhancing luminescence but reducing photostability. Conversely, distancing the cation leaves the complex within a more symmetrical solvent enclosure, decreasing luminescence and increasing photostability. Our findings also indicate that exposing Eu(III) complexes to UV light can function as a method for the photochemical synthesis of Eu(II) complexes, a process further substantiated by our electrochemical experiments.

## Abstract 2

Ultraviolet photobleaching is a phenomenon that arises from the nonlinear, energy-dependent, and irreversible photoreduction of Europium(III) in the complex.

## Introduction

Luminescent lanthanide complexes, particularly β-diketonate europium(III) complexes, which stand out for their exceptional luminescence, play a pivotal role in several applications.^1^ β-diketonate Eu^3+^ complexes, however, face challenges related to their low photostability, which occasionally poses an obstacle to their utilization.^2^ To circumvent this issue, it is often necessary to embed them in solid matrices, such as polymers,^3,4^ zeolites,^5,6^ OLEDs,^7,8^ etc. Therefore, unraveling the intricacies of this photoinstability is crucial for understanding the fundamental aspects that hinder the exploitation of the potential of these complexes in diverse technological contexts.

Certainly, many trivalent lanthanide (Ln^3+^) coordination compounds exhibit strong luminescent properties, positioning them as promising materials for diverse applications such as barcode,^9,10^ anticounterfeit materials,^11^ luminescent thermometers,^12^ probes and sensors,^13,14^ functional coordination polymers,^15^ theranostics,^16^ electrochemiluminescence immunoassay,^17^ and investigation of cellular environments.^18^ Indeed, complexes featuring β-diketonate ligands are acknowledged for their exceptional luminescence, ranking among the most radiant europium coordination compounds.^1^ Luminescence due to two-photon absorption arising from femtosecond laser pulses over a spectral range of 730–830 nm has also been reported for the europium β-diketonate complex [Eu(tta)_3_(dpbt)], where tta stands for thenoyltrifluoroacetonato and dpbt stands for 2-(N,N-diethylanilin-4-yl)-4,6-bis(3,5-dimethylpyrazol-1-yl)-1,3,5-triazine. This compound displays efficient two-photon sensitization and high-purity red emission.^19^ Despite their qualities, these complexes often display instability, and their luminescence diminishes upon exposure to UV radiation. Turning a potential setback to a notable advantage, this luminescence fading upon UV exposure inspired Santa-Cruz et al. to develop a portable dosimeter using these complexes as the emitting layer in an OLED device,^20^ on functional inks printed on cellulose sheets,^21^ and in a printed UV molecular dosimeter strip for monitoring cutaneous vitamin D3 production.^22^ Nevertheless, despite commonly being referred to as “photodegradation” or “photobleaching”, and prompting various mitigation strategies, this gradual dimming associated with photoinstability poses a significant challenge that could potentially limit the effectiveness of devices utilizing such complexes for the majority of other applications, such as luminescent solar concentrators.^23^ Other researchers have focused on understanding the mechanism of photodegradation or photobleaching, a phenomenon that, when observed in β-diketonates, seemingly does not depend on the ligand type.^24^ More recently, Kovacs and Borbas^25,26^ addressed this phenomenon as the transfer of an electron from the excited state of a donor ligand or antenna to an acceptor Eu(III) central metal ion upon photoexcitation. According to the authors, because Eu(III) ions have a relatively low reduction potential compared to other lanthanides, Eu(III) redox states are easily accessible to excited-state aromatics in the complexes. The authors concluded that the resulting electron transfer contributed significantly to the luminescence quenching in the Eu complex. Resistance to this photochemical process has been linked to the rigidity of the molecular structure of the ligands as indicated, for example, in the case of 4-hydroxy-1,5-naphthyridine.^27^ Likewise, the incorporation of the complexes into matrices, seems to offer a stable framework for the molecules, shielding them from the adverse impact of different energy oscillators (C–H, C–C, or C–N) and intermolecular collisions as stated by Iftikhar.^28^ Alternatively, deflecting incident radiation away from other detrimental photochemical or photophysical processes, by a “photo-click” trans-to-cis isomerization of ligands upon continuous UV-A exposure, has been proven to improve photostability for the case of the t-bpete (4,4,4-trifluoro-1-phenyl-1,3-butanedionate).^24^

Remarkably, there is a lack of studies discussing the detection of species after the presumed photodegradation, with few exceptions.^29−31^ Although various types of photon-induced chemical damage have been attributed, the fundamental reasons behind this seemingly irreversible and continuous decrease in emission intensity remain unclear.

Nonetheless, this phenomenon, commonly referred to as photodegradation, is frequently investigated by analyzing the luminescence decay curve over time.^28−30,32−36^ This decay curve is sometimes modeled as a single exponential, and the resulting lifetime is used as a metric to assess the photostability of the complex within a solution and across diverse matrices.^28,37^ However, a rigorous examination of photodegradation requires consistent exposure to UV illumination with a stable intensity. Thus, challenges arise when comparing results across articles, owing to variations in the intensity of the employed UV sources and the inherent variability in their spectral power distributions.

The simple fact is that such photodegradation is not primarily a time-dependent phenomenon, but rather an energy-dependent phenomenon in adherence to the Grötthuss–Draper law.^38^ Indeed, in the absence of UV illumination, photodegradation does not occur and remains paused. Thus, the conventional approach of tracking it over time presupposes continuous and stable UV irradiation. However, the most fundamental approach for observing and analyzing this phenomenon, addressing it at its core cause-and-effect relationship, is to measure the dimming of the luminescence intensity as a function of the energy dose, as quantified by a dosimeter. This approach was employed by Barreto et al. in their discovery of a strict correlation between the increase in the refractive index and the quenching of photoluminescence in [Eu(dbm)_3_(phen)] thin films.^31^ Analogously, albeit with a more inventive twist, Santa Cruz and co-workers developed a UV sensor to monitor the cutaneous production of vitamin D_3_.^21,22^

**Figure 1:**
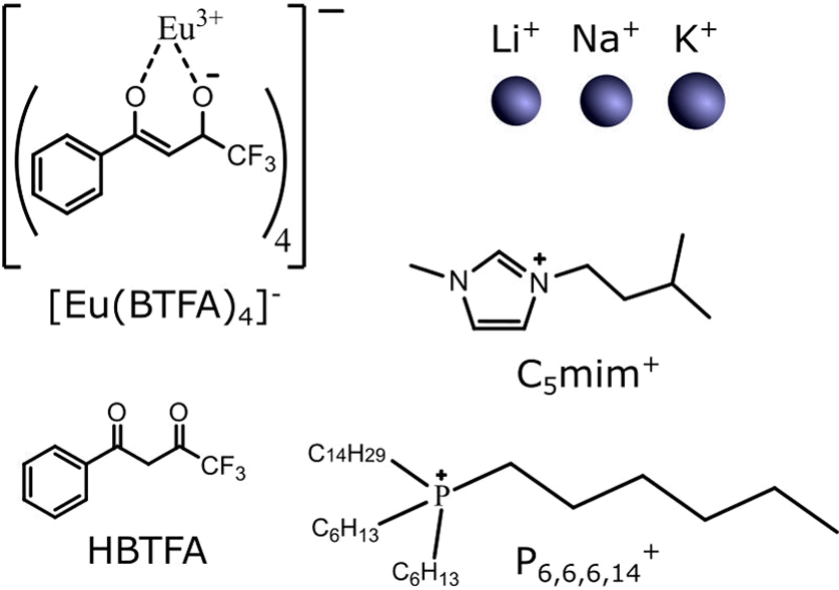
Chemical structures of the species. Only the keto form of HBTFA is shown, though it exhibits keto-enol tautomerism.

In this article, we address the issue of the photostability of europium β-diketonate complexes X[Eu(BTFA)_4_], where X represents Li^+^, Na^+^, K^+^, C_5_mim^+^, or P_6,6,6,14_^+^ (Figure 1). The focus is on the diminishing luminescence during prolonged ultraviolet (UV) exposure. Our research findings indicate that an energy-driven photochemical process that causes the reduction of Eu(III) to Eu(II) is the reason behind the consequent dimming of luminescence. To confirm this photoreduction process we obtained emission spectra, and employed electrochemical techniques, specifically cyclic voltammetry and chronoamperometry,^39,40^ which provided direct evidence of the Eu^3+^/Eu^2+^ reduction and the disappearance of Eu(III) after UV exposure. Extended UV exposure resulted in decreased Eu(III) luminescence and heightened Eu(II) luminescence upon direct excitation.

Subsequently, we introduce an energy-based kinetic law, defining the concept of photobleaching energy, to compare the photoreduction dynamics across complexes and solvents. Our study further highlights the impact of counterion nature and distance on the photostability of tetrakis europium complexes: organic cations and greater counterion distances lead to slower photoreductions. Ultimately, the nonlinearities revealed by the energy-controlled kinetics suggest a multiphoton process influenced by the counterion and solvent type. These results provide new insights into the photochemical and electrochemical behavior of β-diketonate Eu(III) complexes, which will be further elaborated upon in the Conclusions section.

## Results and Discussion

The studied complexes were europium β-diketonate complexes X[Eu(BTFA)_4_], where X represents Li^+^, Na^+^, K^+^, C_5_mim^+^ (1-(2-methyl-butyl)-3-methyl-imidazolium), P_6,6,6,14_^+^ (trihexyl(tetradecyl)phosphonium), or BTFA (4,4,4-trifluoro-1-phenyl-1,3-butanedionate) (Figure 1). All complexes were prepared using a microwave-assisted synthesis technique developed in our laboratory,^41^ followed by characterization using various analytical techniques, including infrared spectroscopy (IR), nuclear magnetic resonance (NMR) spectroscopy, matrix-assisted laser desorption/ionization time-of-flight mass spectrometry (MALDI-TOF), and elemental analysis. Details of the syntheses and analyses are discussed in subsequent sections of this article.

### The Irreversible Photoreduction of Eu(III) to Eu(II)

To investigate this photobleaching phenomenon, we conducted a series of excitation experiments on chloroform solutions of the three complexes with the counterions Li^+^, K^+^, and P_6,6,6,14_^+^. Initially, the samples were highly luminescent. After subjecting the samples to UVC radiation for a few hours, a decrease in luminescence was detected. In some instances, a complete halt in emitted light was observed.

Merely claiming that the samples underwent photodegradation would not be adequate for our needs. Furthermore, the Eu(III) luminescence is highly sensitive to the presence of ligands. Therefore, the complete cessation of luminescence led us to conclude that Eu(III) metal ions were no longer present in the completely photobleached samples. Hence, we decided to explore the mechanism described by Kovacs and Borbas,^25^ which involves the transfer of an electron from the excited state of the ligands to the acceptor Eu(III) central metal ion upon photoexcitation, resulting in the formation of its reduced form, Eu(II). In the current study, we noticed that this photoreduction process is irreversible based on our empirical observations of the phenomenon.

The direct Eu(II) excitation wavelength falls between 260 and 395 nm,^42−46^ overlapping with the ligands excitation range. Given that we observed luminescence from Eu(II) in the completely photobleached samples, we conducted experiments to pinpoint the optimal excitation wavelength for intense Eu(II) ion emission in our samples. We determined that the optimal wavelength was 370 nm, which we then used in our experiments to capture the simultaneous emission spectra from both Eu(III) and Eu(II) metal ions within an expanded acquisition window.

**Figure 2:**
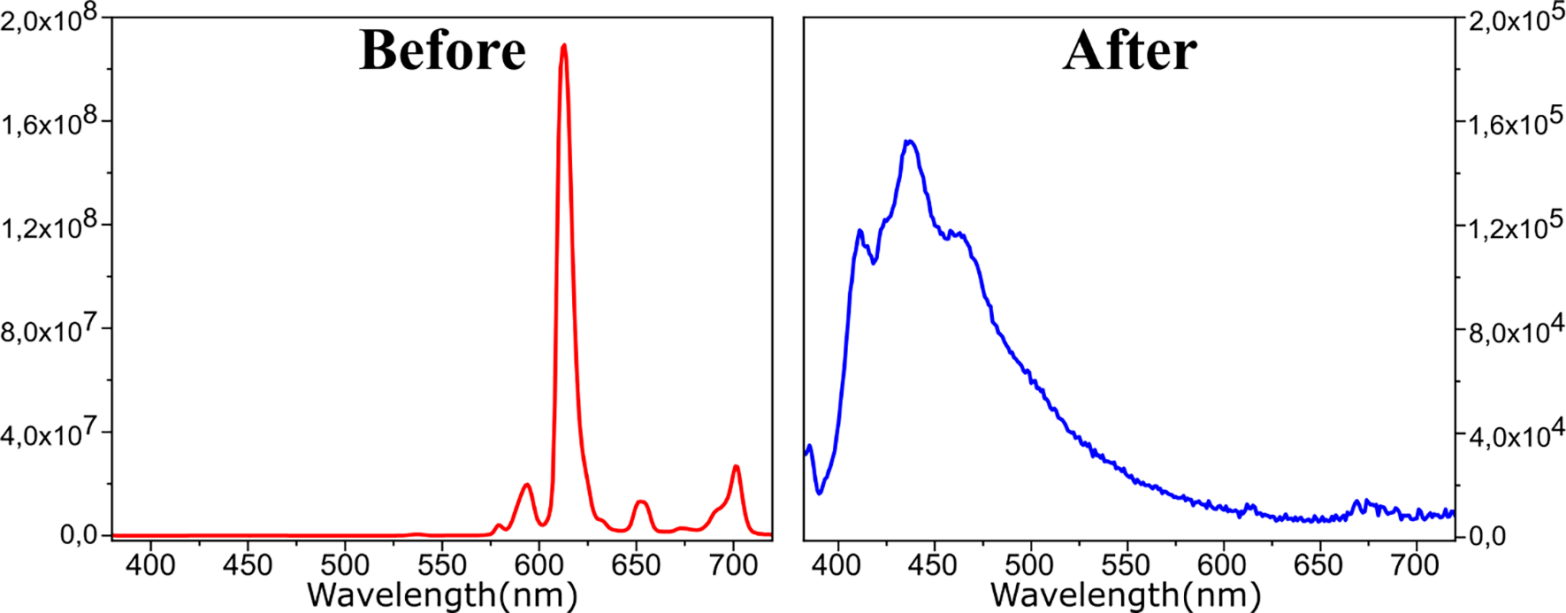
Emission spectra of a 1 × 10^–4^ M solution of P_6,6,6,14_ [Eu(BTFA)_4_] in chloroform before (left) and after (right) complete photobleaching with UVC light. The intensity units are arbitrary but identical for both cases, indicating the relative magnitude of the emission intensities from both samples.

The resulting emission spectra exhibit significant differences. Figure 2 shows the emission spectra of P_6,6,6,14_[Eu(BTFA)_4_] before and after UVC irradiation. Figures S42 and S43 in the Supporting Information show the equivalent spectra for the same complex with Li^+^ and K^+^ counterions with similar characteristics. From these figures, one can observe that before initiating UVC irradiation of the samples, only emission peaks from 575 to 720 nm, characteristic of Eu(III), can be observed. After complete photobleaching, the emission intensity of Eu(III) ions was suppressed and Eu(II) emission was observed in the 400–550 nm region. Additionally, in Figure S44, we show the emission spectrum of an acetonitrile solution of the P_6,6,6,14_[Eu(BTFA)_4_] complex in the middle of the photobleaching process, displaying the appearance of a band corresponding to Eu(II) along with peaks typical of Eu(III), indicating an ongoing photoreduction process. Hence, this transition in emission from the red to the blue region indicates that the photobleaching process induces irreversible photoreduction of Eu(III) to Eu(II). Our endeavors to reverse this process and oxidize Eu(II) to Eu(III) in completely photobleached samples proved unsuccessful, underscoring irreversibility as a distinctive characteristic of the process.

### Conjecture on the Photoreduction Mechanism

Chemical reactions are complex and multifaceted processes. Therefore, we do not claim that what will be presented here is the actual chemical mechanism taking place. Our intention was to present a conjecture of what could be happening in chloroform solvent upon UV illumination in the form of an overall stepwise and stoichiometrically correct logical structure to guide our investigation. We begin with the proposal by Kovacs and Borbas^25^ of the transfer of an electron from the excited state of the ligands to the acceptor Eu(III) central metal ion upon photoexcitation. Below, we outline the photoreduction reaction in a stepwise manner for clarity and illustrative purposes:
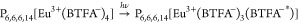
where BTFA^–^* represents the anion BTFA^–^ in an excited state. Then,
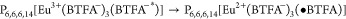


Subsequently, the neutral radical •BTFA is decoordinated as follows:
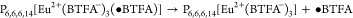


Simultaneously, upon UV irradiation, chloroform undergoes homolytic dissociation, followed by secondary reactions,^47^ of which the relevant reactions are
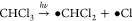

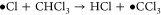


Hydrogen chloride now reacts with •BTFA:
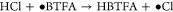


There are now many possibilities for the free radicals •CCl_3_ and •Cl to react as well as to engage in other chemical reactions, potentially forming many species. We simply point out that what should be logically expected is that HBTFA is formed as a byproduct of the photoreduction of P_6,6,6,14_[Eu^3+^(BTFA^–^)_4_]. This conjectured mechanism is consistent with the observed irreversibility of the photoreduction process.

### Confirmation of Eu^3+^/Eu^2+^ Ion Reduction of the P_6,6,6,14_[Eu(BTFA)_4_] Complex via Cyclic Voltammetry and Chronoamperometry

**Figure 3:**
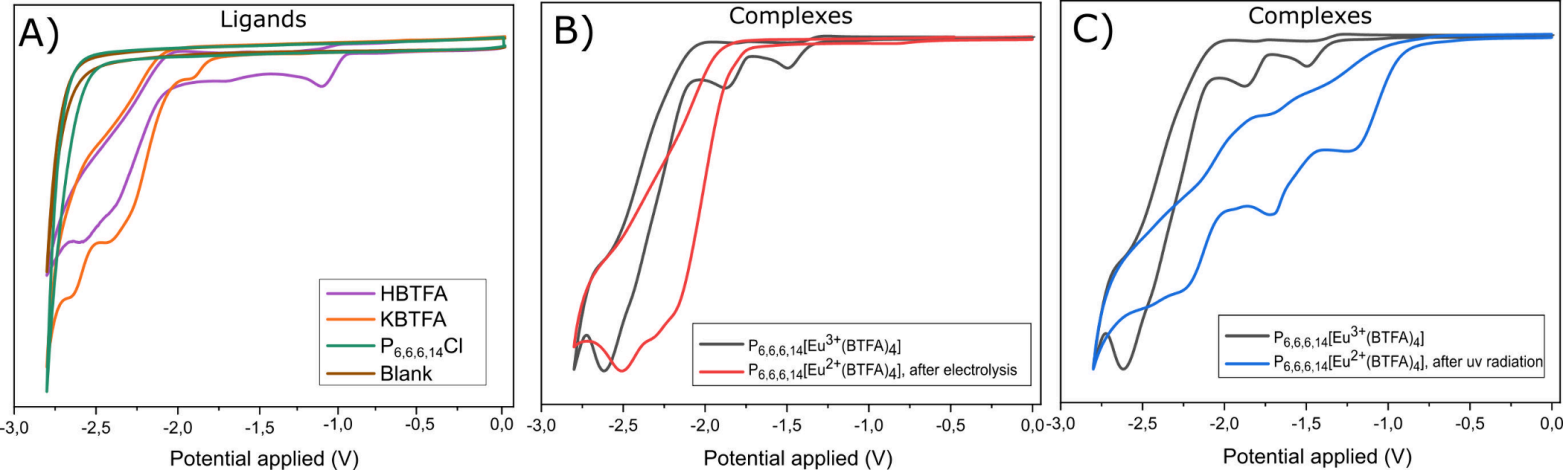
(A) Cyclic voltammograms of the blank (brown line), 10.0 mmol·L^–1^ KBTFA (orange line), 10 mmol·L^–1^ P_6,6,6,14_Cl (green line), 10 mmol·L^–1^ HBTFA (purple line). (B) Cyclic voltammograms of the 10 mmol·L^–1^ P_6,6,6,14_[Eu^3+^(BTFA)_4_] solution before (black line) and after electrolysis (red line, Q = 12.5 C), and (C) cyclic voltammograms of the same solution before (black line) and after UV photoirradiation (blue line). All experiments were carried out in acetonitrile/0.1 mol·L^–1^ TBABF_4_, v = 100 mV·s^–1^, using Ag/AgCl, 3.0 mol·L^–1^ KCl as the reference electrode.

Cyclic voltammetry experiments were carried out to determine the Eu^3+^/Eu^2+^ reduction pair potential in the P_6,6,6,14_[Eu^3+^(BTFA)_4_] complex before photoreduction, the absence of Eu^3+^, and the presence of free ligands in the solutions after photoreduction, as conjectured in the previous section. First, cyclic voltammograms of the salts 10 mmol·L^–1^ K(BTFA) and 10 mmol·L^–1^ P_6,6,6,14_Cl were registered in acetonitrile/tetra-*n*-butylammonium tetrafluoroborate (0.1 mol·L^–1^ TBABF_4_) medium to determine the cathodic potential window of the experiments. A glassy carbon (GC) was used as the working electrode (WE) at a scan rate of 100 mV·s^–1^. Figure 3A (Orange line) shows cyclic voltammogram of the K(BTFA) salt, where it can be observed three reduction peaks at −1.90, −2.42, and −2.64 V vs Ag/AgCl, attributed to the [BTFA]^−^ reduction. None of the reduction peaks presented the corresponding oxidation peaks after scan inversion, indicating irreversible behavior (Figures S66 and S67). Figure 3A (green line) also shows the cyclic voltammogram of P_6,6,6,14_Cl salt, and no peak was observed in the cathodic region, showing electron inactivity on the GC electrode surface.

Let us now consider the complex before photoreduction with UV light. Thus, a potential window between −1.80 to 0.0 V vs Ag/AgCl could be explored to determine the reduction potential of the P_6,6,6,14_[Eu^3+^(BTFA)_4_] complex. Figure 3B (black line) shows the scanning of the cathodic region for the 10 mmol·L^–1^ Eu complex solution; the first peak was observed at −1.49 V vs Ag/AgCl. No oxidation peak was observed after scan inversion (Figure S73), indicating irreversible behavior. This reduction peak potential is in agreement to the Eu^3+^/Eu^2+^ pair electron transfer potential ascribed for other Eu complexes: Eu(hfa)_3_(H_2_O)_2_ (−1.65 V vs Ag/Ag^+^, ITO-WE),^48^ Eu^3+^(DOTA) (−1.10 V vs Ag/AgCl, GC-WE).^49^ Other two reduction peaks were observed at −1.86 V and −2.61 V (Figure 3B (black line)), which are associated with the reduction peaks of the [BTFA]^−^ ligand, as previously described in the K[BTFA] cyclic voltammogram, Figure 3A (orange line).

In sequence, a controlled potential electrolysis (−1.50 V vs Ag/AgCl) was carried out in the same V-cell, using a glassy carbon rod as WE, and a Pt wire as auxiliary electrode placed in a separated compartment. Electrolysis was performed until the Eu^3+^ complex luminescence completely disappeared, passing a total charge of 12.5 C. A cyclic voltammogram of the electrolyzed solution was obtained (Figure 3B), showing two peaks at −2.23 and −2.50 V, which are associated with the BTFA^–^ ligand. Thus, the reduction peak previously observed for the Eu^3+^/Eu^2+^ pair (−1.50 V) disappeared, indicating the total consumption of Eu^3+^ ions in the solution.

Let us now consider the P_6,6,6,14_[Eu^3+^(BTFA)_4_] complex solution after UV irradiation in chloroform. Figure 3C (blue line) shows the cyclic voltammogram of the putative P_6,6,6,14_[Eu^2+^(BTFA)_3_] solution together with dissolved P_6,6,6,14_[BTFA] and HBTFA, among other species. It is immediately evident the disappearance of the Eu^3+^/Eu^2+^ pair reduction peak (−1.49 V, black line), indicating the total Eu^3+^ complex consumption. The two peaks observed at −2.23 V and −2.45 V can be associated with the [BTFA]^−^ ligand, as ascribed on the cyclic voltammogram of the Eu complex electrolyzed solution Figure 3A (orange line).

However, other two reduction peaks at −1.2 V and −1.7 V vs Ag/AgCl can be observed. To explain the species causing the appearance of these two new peaks, the electrochemical behavior of HBTFA, which is a species that can be formed during the conjectured photodegradation process, was investigated. A cyclic voltammogram of 10 mmol·L^–1^ HBTFA solution showed peaks at −1.12 V, −1.7 V, –2.39 V, and −2.59 V vs Ag/AgCl (Figure 3A (purple line)). Therefore, the first and second reduction peaks observed on the HBTFA solution are in agreement with the peaks observed on the UV-photoirradiated Eu complex solution (E = −1.2 and −1.7 V, respectively, Figure 3C (blue line)).

As a result, the electrochemical analyses, after the photoreduction of P_6,6,6,14_[Eu^3+^(BTFA)_4_] provided, as conjectured, evidence of the presence of the following species in solution: HBTFA, [BTFA]^−^; as well as of the absence of Eu^3+^. Other species may be present, but could not be identified at this stage.

### Chronoamperometry and Luminescence

**Figure 4:**
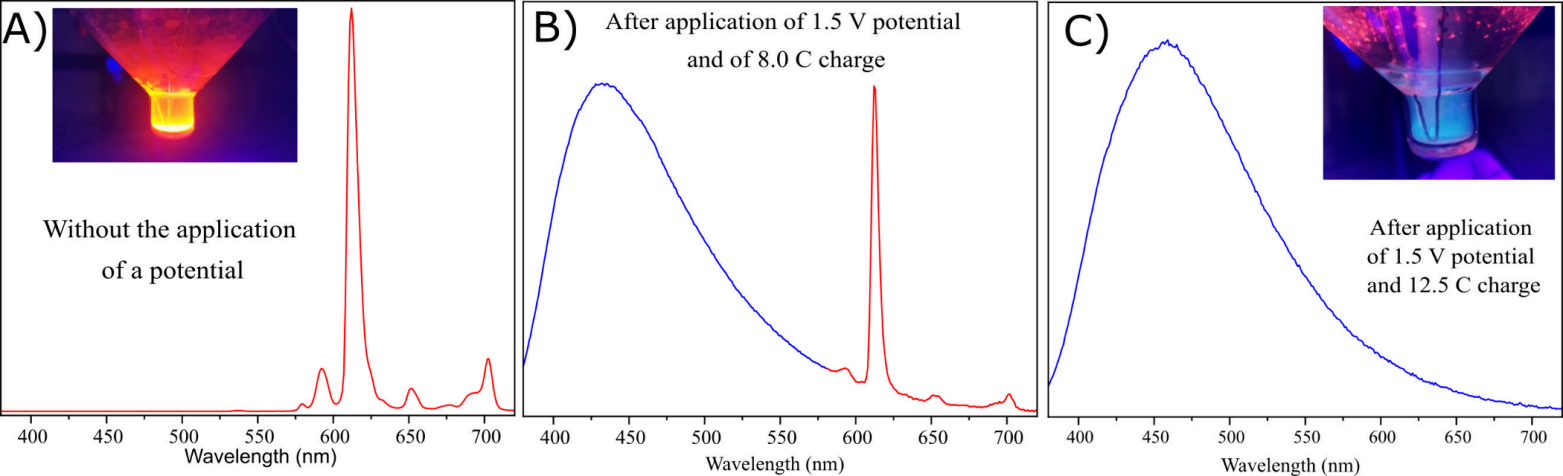
Emission spectra of a 3 mmol·L^–1^ solution of P_6,6,6,14_[Eu^3+^(BTFA)_4_] in acetonitrile with excitation at 370 nm. Spectra were recorded at three stages of electrolysis at −1.5 V: (A) before electrolysis, (B) during electrolysis after passing a charge of 8.0 C, and (C) after completing electrolysis with a total charge of 12.5 C passed. Acetonitrile was used as the solvent and λ _exc_ = 370 nm.

To confirm that the observed “photodegradation” of P_6,6,6,14_[Eu^3+^(BTFA)_4_] is in fact a photoreduction, we performed an electrochemical reduction of Eu^3+^ to Eu^2+^ via electrolysis. By comparing the emission spectrum obtained after this electrochemical reduction with that of the photoreduced complex, we aimed to provide strong evidence that illuminating the solution of the complex indeed photoreduces Eu^3+^ to Eu^2+^. Thus, controlled potential electrolysis experiments were performed and accompanied by emission spectroscopy to investigate the emergence of the Eu^2+^ emission band during and after electrochemical reduction. Before electrolysis, the emission spectrum of the electrolyzed solution was recorded, showing the expected f–f transition bands of the excited P_6,6,6,14_[Eu^3+^(BTFA)_4_] complex in the range of 570–720 nm (Figure 4A). Following the transfer of a charge totaling 8.0 C, a new emission band appeared between 400 and 550 nm and it was attributed to mostly d–f transitions in Eu^2+^, observed during the reduction of Eu^3+^ to Eu^2+^ (Figure 4B).^48^

The electrolysis process continued, requiring an additional charge of 4.5 C to achieve the complete disappearance of the Eu^3+^ emission bands, leaving only the Eu^2+^ emission band (Figure 4C), which exhibited a bluish tint visible to the naked eye. The theoretical charge necessary for the reduction of Eu^3+^ is 2.98 C (Q = *n*.*z*.*F*), where *n* is the reagent mole number, *z* = number of electrons involved, and *F* is the Faraday constant = 96,485 s·A·mol^–1^). However, an excess of electrons was necessary to complete the electrochemical reduction of the complex, which can be attributed to the rearrangement of the coordination structure of the Eu complex, along with the discoordination of [BTFA]^−^ and subsequent protonation as conjectured. Indeed, the excess of charge necessary for the total consumption of the Eu^3+^ present in the reaction solution can be a result of the electroreduction of HBTFA in parallel to the Eu^3+^ complex, which explains why HBTFA was not observed after the electrolysis process because it is an electroactive species (−1.12 V) at the potential applied of −1.5 V. Hence, HBTFA was identified only during the UV photoirradiation process. Consequently, the excess charge provides additional evidence of the discoordination of BTFA^–^ from Eu^2+^ in both processes. Finally, additional charge was required because of the possible residual water present in acetonitrile.

Thus, the cyclovoltammetric analysis of the UV-photoirradiated and electrolyzed solutions of the P_6,6,6,14_[Eu^3+^(BTFA)_4_] complex proved that the reduction of the Eu^3+^ occurs, giving Eu^2+^ which was determined by the appearance of the Eu^2+^ emission band in the range 400–550 nm, as described in the literature.^48^

### Quantifying Photostability: Theory

As highlighted in the Introduction, previous studies have assessed the photostability of different samples by comparing the kinetic first-order time decay constants.^28,37^ However, the photobleaching phenomenon is not primarily time-controlled, but rather energy-controlled, requiring UV illumination to be exposed continuously and at an uninterrupted and constant irradiance for all different samples if time decay is to be comparatively meaningful. Variations in UV lamp radiation intensity can occur during experiments, and the utilization of UV lamps with differing power outputs has hindered accurate sample comparisons among different research groups.^24,27−29,33,34,50^ A more systematic and standardized approach to data analysis is required to ensure reliable and meaningful conclusions regarding the photostability of these complexes.

Accordingly, we have devised a different approach to thoroughly investigate and quantify the photostability of europium tetrakis complexes, and introduced the concept of “photobleaching energy,” as the energy required to reduce the amount of emitting Eu(III) to a fraction 1/e of the original amount in our samples. Our method enhances robustness by accounting for nonuniformity in the reported data, mitigating the challenges associated with comparing samples reported in the literature, because it relies solely on the accumulated dose of incident radiation on the sample. The dose was meticulously controlled by the equipment, thereby minimizing significant variations. Seeking to expand, refine, and specifically target our understanding of the photobleaching phenomenon in europium complexes as energy-controlled phenomena, we now introduce a formalism to study this process solely in the energy domain.

As a given number of photons illuminate a sample comprising Eu(III) complex molecules M dissolved in a solvent, a number of complex molecules, in proportion to their concentration, are placed in an excited state M* according to the well-known Stark-Einstein law of photochemistry.
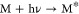
with [M*] being proportional to [M]. Once in the excited state, two scenarios may occur, both in proportion to [M]: either M* luminesces as Eu(III), or M* undergoes photoreduction of the Eu(III) to produce Eu(II), which we express in terms of the concentration of [M] in the following form:
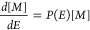
1where, P(E) is the photobleaching function. By separating the variables and expanding P(E) in a Taylor series around E = 0, a situation corresponding to the outset of the experimental process when the sample has not yet received any radiant energy, eq 2 becomes
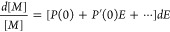
2

As the intensity of luminescence photons, I, is directly proportional to the fraction of M* that luminesces, which is proportional to M, we can replace M by I and integrate the equation to obtain
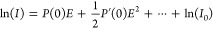
3where I_0_ is the luminescence intensity at the beginning of the experiment. We now define the photobleaching constants as κ_*1*_*= P(0)*; κ_*2*_*= P’(0)/2*; so that eq 4 now becomes
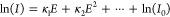
4

Of course, the prerequisites for the validity of these equations are (i) that the solution must be diluted (generally on the order of 10^–4^ M) to avoid concentration quenching, which is the result of strong interactions among dissolved complex molecules;^51,52^ (ii) the solute must be pure to avoid effects of other materials present in the solution;^53,54^ and (iii) the source of incident light should not be inhomogeneous or too intense (as is the case with a powerful laser) to avoid other effects.^55^ The origin of the nonlinear effects, implied by the presence of κ_2,_ is the result of a complicated variety of factors^55^ and can perhaps be interpreted as arising from two-photon absorptions. The fact is that we observed these nonlinear effects. They are real and deserve mention, especially since they appear to follow regular patterns.

If we were to truncate the above equation to the first order, the equation would then become compatible with what is implied by the Bunsen-Roscoe law^56,57^ that in our case, the amount of photoreduced Eu(II) is determined by the total amount of radiant energy incident on the sample:
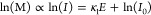
5

Of course, κ_*1*_ is always a negative quantity. In this first-order approximation, we can now define the concept of photobleaching energy E^PB^ as the amount of incident radiant energy E needed to reduce the initial luminescence intensity in our samples to a fraction 1/e as being *E*^*PB*^ = −1/κ_*1*_ in the first order approximation. Likewise, we define the half-photobleaching energy as *E*^*PB*^_*1/2*_ = −ln(2)/κ_*1*_. These can now be used to quantify and compare the photostability of different Eu(III) complexes, under our strictly controlled experimental conditions.

However, it is necessary to consider eq 6, which has been truncated to the second order, in light of our findings.
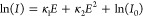
6

In this case, the photobleaching energy *E*^*PB*^ is calculated as
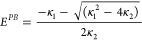
7

We can also define the half-photobleaching energy as the energy required to halve the luminescence intensity in our samples. Accordingly, the half-photobleaching energy, *E*^*PB*^_*1/2*_, can be expressed as
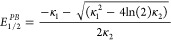
8

### Quantifying Photostability: Experiment

The experimental methodologies for the photostability and luminescence investigations are detailed in the subsequent sections of this manuscript. Utilizing the LUMPAC software,^58^ we acquired photophysical data for the complexes, encompassing quantum efficiency values (η), radiative decay rates (A_rad_), nonradiative decay rates (A_nrad_), and lifetime (τ).

Photostability measurements were conducted by subjecting diluted solutions of [Eu(BTFA)_4_]^−^ to UVA light exposure, spanning a cumulative radiation dosage ranging from 0.0 to 10.0 J/cm^2^, with increments of 0.5 J/cm^2^. Our endeavors to discern the influence of composition, structure, and interionic distancing in solution on complex photostability prompted the adoption of two strategies: variation of solvents with different polarities, specifically chloroform and dichloromethane as nonpolar solvents, and acetone and acetonitrile as polar solvents, and the utilization of counterions from different classes: the alkaline inorganic cations Li^+^, K^+^, and Na^+^, and the organic ones C_5_mim^+^ and P_6,6,6,14_^+^.

**Figure 5:**
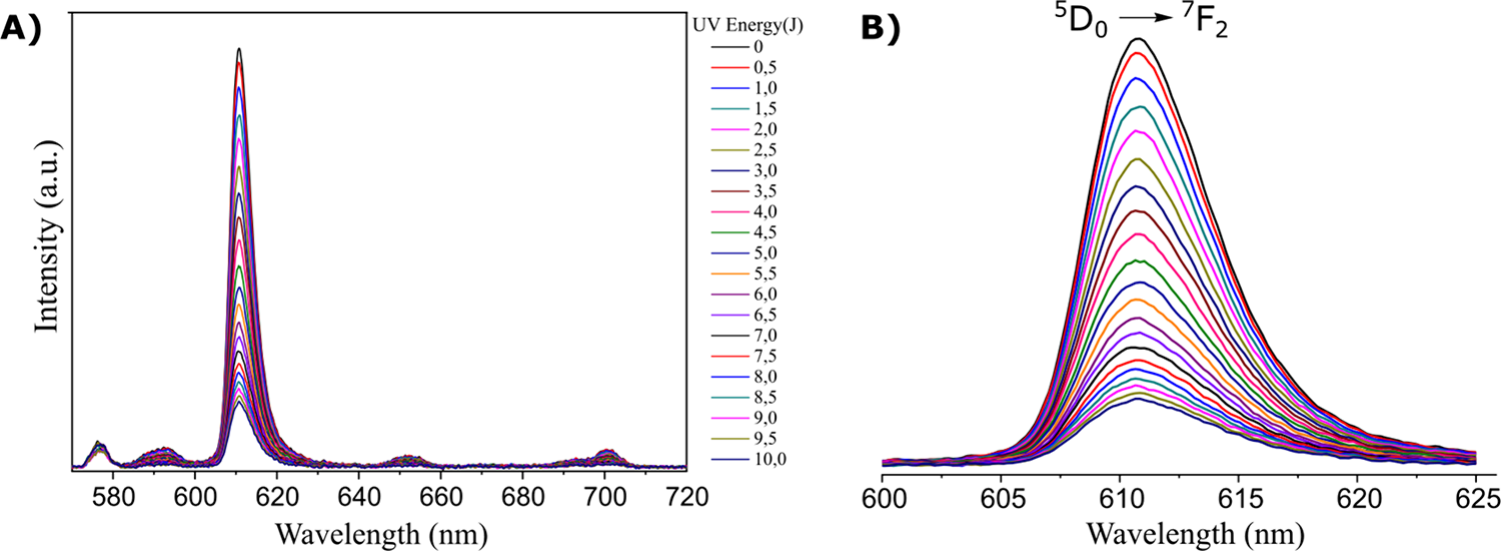
(A) Emission curves of C_5_mim[Eu(BTFA)_4_] complex in chloroform after increasing doses of UVA irradiation. (B) Magnified view of the hypersensitive transition ^5^D_0_ → ^7^F_2_.

Figures 5A and 5B show a representative decay of the emission intensity of the C_5_mim[Eu(BTFA)_4_] complex in chloroform as a function of the accumulated energy applied. From top to bottom, each curve was obtained by adding increment doses of 0.5 J. In Figure 5B, we magnified the hypersensitive ^5^D_0_ → ^7^F_2_ transition, the peak values of which were used in eq 6 as the emission intensity (I).

**Figure 6:**
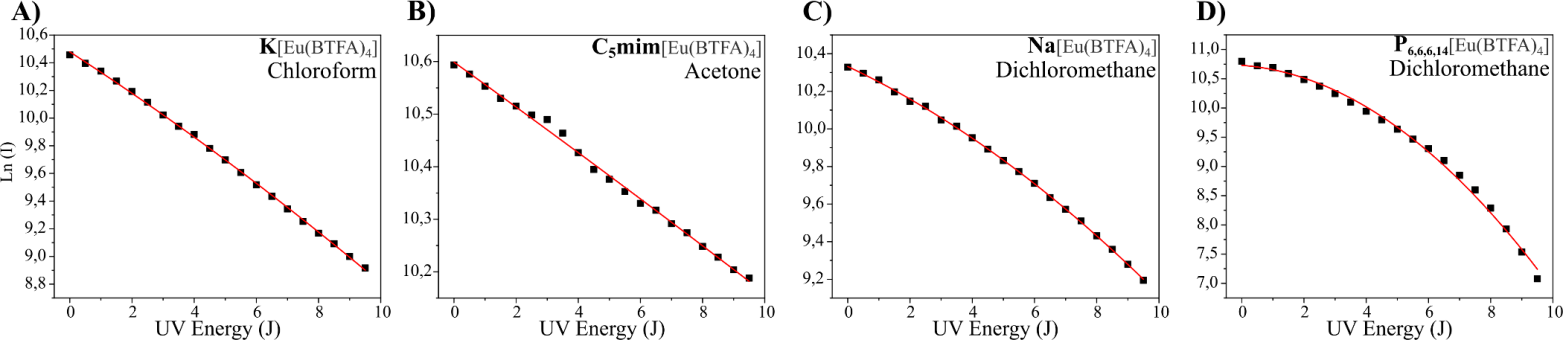
Polynomial adjustment ln(I)= κ_1_ + κ_2_E + κ_3_E^2^ as a function of the UV energy. A) K[Eu(BTFA)_4_] in chloroform; B) C_5_mim[Eu(BTFA)_4_] in acetone; C) Na[Eu(BTFA)_4_] in dichloromethane; and D) P_6,6,6,14_[Eu(BTFA)_4_] in dichloromethane.

In situations like, with K[Eu(BTFA)_4_] in chloroform (Figure 6A) and C_5_mim[Eu(BTFA)_4_] in acetone (as shown in Figure 6B) the relationship between ln(I) and the accumulated incident energy may initially seem linear as, suggested by eq 5. However, for Na[Eu(BTFA)_4_] (Figure 6C) and P_6,6,6,14_[Eu(BTFA)_4_] (Figure 6D) in dichloromethane, the behavior is clearly nonlinear, as implied by eq 6, leading us, in the present article, to employ eq 6 and calculate E^PB^ using eq 7 for all scenarios discussed.

The photobleaching energy, E^PB^, represents, as we defined, the energy required to reduce the Eu(III) emission intensity of our samples by a fraction equal to 1/*e* of the initial intensity. This value is a measure of the resistance to photoreduction of a lanthanide complex sample to UV radiation exposure under given experimental conditions. Consequently, this method allows for the evaluation of both the qualitative and quantitative aspects of the photostability of luminescent lanthanide complexes.

### Quantifying Photostability: Results

Values and their averages by solvent and by counterion of photobleaching energy, E^PB^, of quantum efficiencies of luminescence, η, and of the radiative, A_rad_, and nonradiative, A_nrad_, decay rates are presented in Tabs. 1, 2, 3, and 4, respectively.

**Table 1:** Photobleaching Energy Values (E^PB^) for all [Eu(BTFA)_4_]^−^ Complexes in Solutions of the Four Different Solvents Studied^a^

	**Photobleaching Energies, E**^**PB**^**(J)**						
**Cations**	**Solvents**						
	**Nonpolar**			**Polar**			**AVG.**
	CHCl_3_	CH_2_Cl_2_	**Avg. Np.**	(CH_3_)_2_CO	CH_3_CN	**Avg. P.**	
Li^+^	8.5	5.6	**7.1**	7.5	9.5	**8.5**	**7.8**
Na^+^	3.5	8.7	**6.1**	9.2	17.5	**13.4**	**9.7**
K^+^	6.3	13.4	**9.9**	13.3	14.6	**14.0**	**11.9**
**Avg. Inorg.**	**6.1**	**9.2**	**7.7**	**10.0**	**13.9**	**11.9**	**9.8**
C_5_mim^+^	5.5	9.3	**7.4**	21.8	16.9	**19.4**	**13.4**
P_6,6,6,14_^+^	2.0	4.8	**3.4**	9.0	20.6	**14.8**	**9.1**
**Avg. Org.**	**3.8**	**7.1**	**5.4**	**15.4**	**18.8**	**17.1**	**11.2**
**AVG.**	**5.2**	**8.4**	**6.8**	**12.2**	**15.8**	**14.0**	**10.4**

a “Avg” implies averages across classes, while “AV*G*″ implies averages across all results from all classes. E^PB^ values correspond to measurements on 1 cm^2^ surfaces of 2.50 mL solutions at a concentration of 1 × 10^−4^ M for all samples.

**Table 2:** Quantum Efficiency Values η for all [Eu(BTFA)_4_]^−^ Complexes in the Four Solvents Studied^a^

	**Quantum Efficiencies, η (%)**						
**Cations**	**Solvents**						
	**Nonpolar**			**Polar**			**AVG.**
	CHCl_3_	CH_2_Cl_2_	**Avg. Np.**	(CH_3_)_2_CO	CH_3_CN	**Avg. P.**	
Li^+^	53.3	50.4	**51.9**	43.1	40.8	**41.9**	**46.9**
Na^+^	52.4	53.5	**53.0**	41.8	40.7	**41.3**	**47.1**
K^+^	49.8	50.8	**50.3**	41.6	40.2	**40.9**	**45.6**
**Avg. Inorg.**	**51.8**	**51.6**	**51.7**	**42.2**	**40.6**	**41.4**	**46.5**
C_5_mim^+^	55.3	44.7	**50.0**	39.1	38.0	**38.5**	**44.2**
P_6,6,6,14_^+^	45.5	42.5	**44.0**	39.4	40.1	**39.7**	**41.9**
**Avg. Org.**	**50.4**	**43.6**	**47.0**	**39.2**	**39.0**	**39.1**	**43.1**
**AVG.**	**51.3**	**48.4**	**49.8**	**41.0**	**39.9**	**40.5**	**45.1**

a “Avg” implies averages across classes, while “AV*G*″ implies averages across all results from all classes

**Table 3:** Values of Radiative Decay Rates, A_rad_, for all [Eu(BTFA)_4_]^−^ Complexes in Solutions of the Four Different Solvents Studied^a^

	**Radiative decay rate, A**_**rad**_**(s**^**–1**^**)**						
**Cations**	**Solvents**						
	**Nonpolar**			**Polar**			
	CHCl_3_	CH_2_Cl_2_	**Avg. Np.**	(CH_3_)_2_CO	CH_3_CN	**Avg. P.**	**AVG.**
Li^+^	798	744	**771**	743	640	**692**	**731**
Na^+^	770	690	**730**	677	636	**657**	**693**
K^+^	694	612	**653**	673	626	**650**	**651**
**Avg. Inorg.**	**754**	**682**	**718**	**698**	**634**	**666**	**692**
C_5_mim^+^	1041	717	**879**	632	597	**615**	**747**
P_6,6,6,14_^+^	854	704	**779**	642	618	**630**	**705**
**Avg. Org.**	**947**	**711**	**829**	**637**	**608**	**622**	**726**
**AVG.**	**831**	**693**	**762**	**673**	**624**	**649**	**705**

a “Avg” implies averages across classes, while “AV*G*″ implies averages across all results from all classes

**Table 4:** Values of Nonradiative Decay Rates, A_nrad_, for All [Eu(BTFA)_4_]^−^ Complexes in Solutions of the Four Different Solvents Studied^a^

	**Non-Radiative decay rate, A**_**nrad**_**(s**^**–1**^**)**						
**Cations**	**Solvents**						
	**Nonpolar**			**Polar**			**AVG.**
	CHCl_3_	CH_2_Cl_2_	**Avg. Np.**	(CH_3_)_2_CO	CH_3_CN	**Avg. P.**	
Li^+^	699	731	**715**	980	931	**955**	**835**
Na^+^	699	599	**649**	943	926	**934**	**792**
K^+^	673	622	**648**	947	933	**940**	**794**
**Avg. Inorg.**	**690**	**651**	**671**	**956**	**930**	**943**	**807**
C_5_mim^+^	843	889	**866**	984	976	**980**	**923**
P_6,6,6,14_^+^	1056	952	**1004**	990	926	**958**	**981**
**Avg. Org.**	**950**	**920**	**935**	**987**	**951**	**969**	**952**
**AVG.**	**794**	**759**	**776**	**969**	**938**	**953**	**965**

a “Avg” implies averages across classes, while “AV*G*″ implies averages across all results from all classes

We next investigated the influence of the counterions on the photostability of the anion complex [Eu(BTFA)_4_]^−^. According to Table 1, the photobleaching energy (E^PB^) of [Eu(BTFA)_4_]^−^ in polar solvents follows the order K^+^ > Na^+^ > Li^+^. This pattern indicates that larger alkaline cations tend to increase photostability. Organic cations, which are larger than alkali metals, generally boost the photostability, albeit in a more nuanced manner, owing to the likely interplay of factors such as steric effects, counterion-induced alterations in the electronic structure of europium anion complexes, and complex solvation.

We have demonstrated^59^ that the proximity between cations and anions in solution, determined through NMR contact ion pair (CIP) and solvent-separated ion pair (SSIP) experiments, plays a critical role in the geometric structure and luminescence properties of Europium (Eu) complexes across different solvents. In nonpolar solvents, the detection of CIPs indicates a close interaction between cations and anions, distorting the coordination environment and decreasing symmetry. This close proximity enhances luminescence, as measured by the quantum efficiencies η (Table 2) but, on the other hand, increases the susceptibility to photoreduction.

In polar solvents, the SSIP data indicate that the complex is separated from the cation and is primarily surrounded by the solvent, promoting a more symmetric atmosphere for the anionic complex and leading to lower quantum efficiencies, less luminescence, and enhanced photostability.

**Figure 7:**
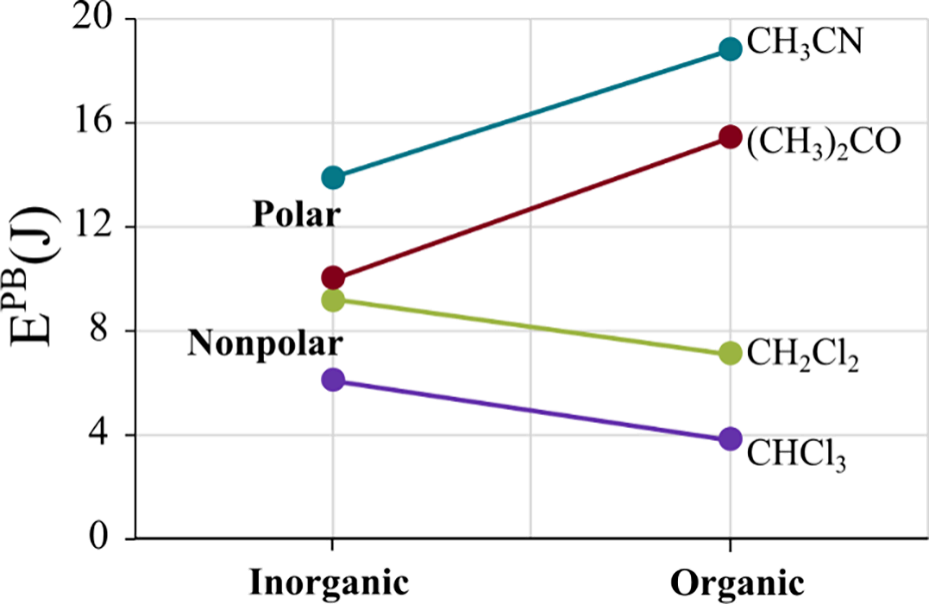
Average photobleaching energy values (E^PB^) for all [Eu(BTFA)_4_]^−^ complexes as a function of the class of counterions for each of the four different solvents employed: dichloromethane, chloroform, acetone, and acetonitrile. The lines are included for visual guidance purposes.

Figure 7 shows the average E^PB^ values grouped by class of counterions (inorganic or organic) for each of the four solvents. By examining Figure 7, we note that the stabilizing/destabilizing effect of inorganic cations with varying solvent polarities is much less impressive than that of organic cations. Indeed, in nonpolar solvents, the proximity of organic cations to the anion complex tends to promote photoreduction, whereas their separation in polar solvents significantly enhances the photostabilization. This solvent effect is notably more pronounced than the influence of inorganic alkaline cations, highlighting the complex interplay between counterion nature and photostability.

As it is known that all four solvents can form free radicals when exposed to UV light,^60−63^ this fact might play a potential role in this behavior. Notably, the lines extending from the inorganic to organic cations displayed parallelism in Figure 7 for each class of solvent polarity. This indicates a similarity in the effects that were comparably modulated by the solvents in each instance.

**Figure 8:**
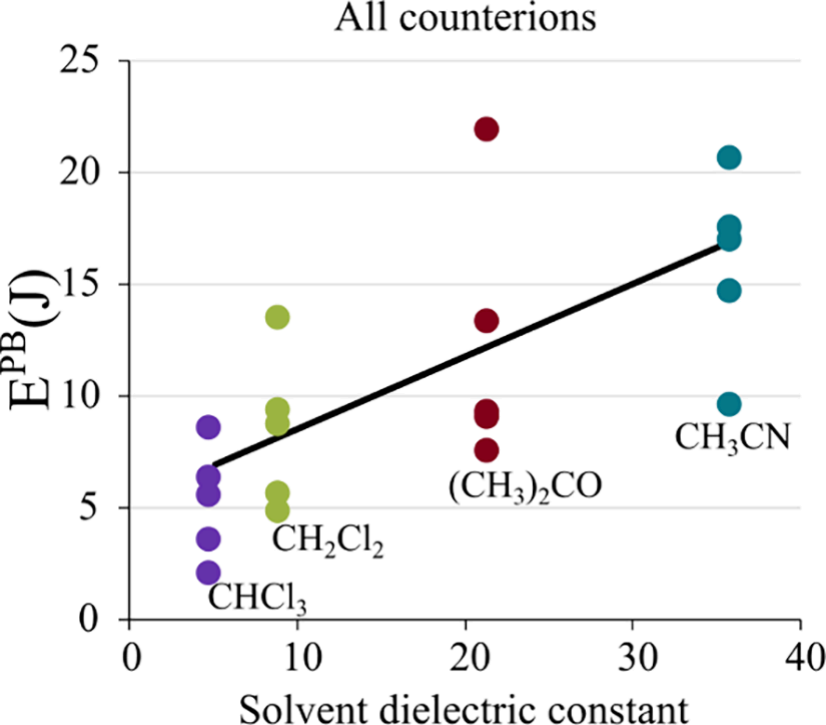
Photobleaching energy values (E^PB^) for all [Eu(BTFA)_4_]^−^ complexes as a function of the dielectric constants of the solvents employed: chloroform (ε = 4.9), dichloromethane (ε = 9.0), acetone (ε = 21.4), and acetonitrile (ε = 35.9). The dotted regression line, calculated by considering all the points, is included to illustrate the general trend.

Figure 8 plots the E^PB^ values of the five compounds against the dielectric constants of the four solvents, revealing a trend in which higher dielectric constants (indicative of higher polarity) are correlated with increased photostability.

This finding suggests that polar solvents provide a protective environment against photobleaching by maintaining solvent-separated ion pairs, whereas in nonpolar solvents, the close proximity of the counterion to the complex with detectable contact ion pairs enhances susceptibility to photoreduction.

**Figure 9:**
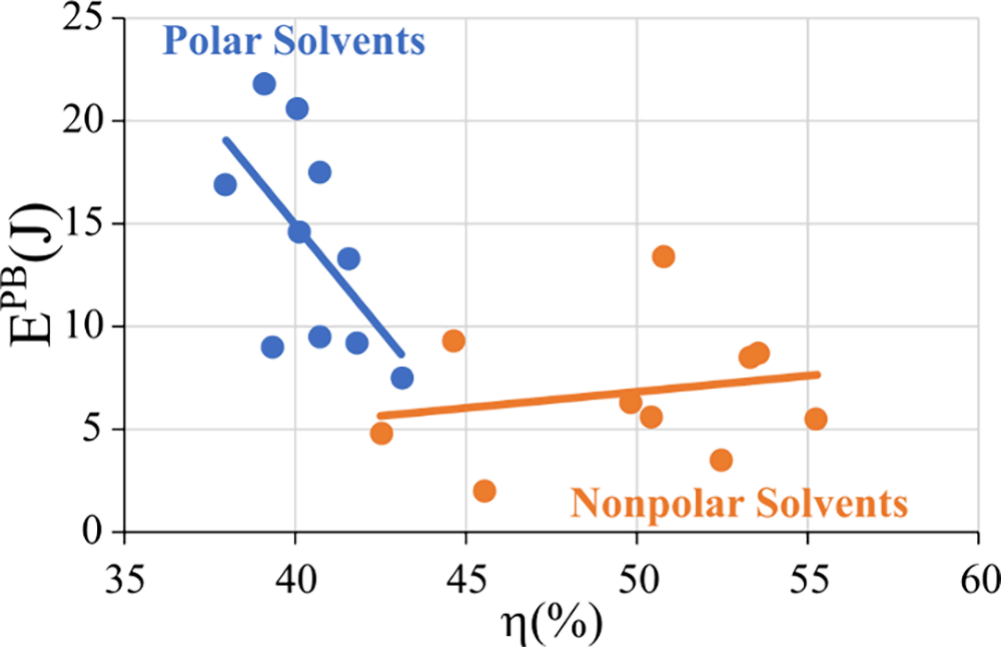
Photobleaching energy values (E^PB^) for all [Eu(BTFA)_4_]^−^ as a function of the quantum efficiencies of luminescence (η), distinguished by color for each class of solvents employed: polar solvents comprising acetone and acetonitrile, and nonpolar solvents comprising dichloromethane and chloroform. Dotted regression lines were calculated for each solvent class.

Now, let us compare the photostability measured by E^PB^ with the luminescence, as represented by the quantum efficiency η for all compounds presented in Table 2 and shown in Figure 9. The quantum efficiencies of luminescence are generally higher in nonpolar solvents because they favor contact-ion pairs,^59^ resulting in distorted coordination polyhedra with lower symmetry, which leads to less forbidden metal centered f-f transitions, and therefore enhanced luminescence. This is usually accompanied by decreased nonradiative decay rates^64^ (Table 4), leaving more complex molecules in the excited state upon UV illumination, which makes them more prone to photobleaching. Conversely, polar solvents favor solvent-separated ion pairs,^59^ which leave the ion complex surrounded by a much more spherically symmetric enclosure, leading to a more spherically symmetric coordination polyhedron, and thus to less luminescence due to more forbidden metal-centered f-f decays and larger nonradiative decay rates^64^ (Table 4). These nonradiative decay rates likely redirect Eu(III) ions from their excited states through alternative pathways, reducing the likelihood of photoreduction and thereby contributing to their photostability. This insight highlights the significance of the solvent environment in modulating the photostability of complexes.

Figure 9 shows the quantum efficiency (η) and E^PB^ values for the different solvents. Upon examination of Figure 9, two distinct sets of data points were evident. The first set represents complexes in polar solvents with lower η values ranging from 38.0% to 43.1% and higher E^PB^ values from 21.8 to 7.5 J indicating resistance to photobleaching.

Conversely the second set includes complexes in nonpolar solvents with η values ranging from 55.3% to 42.5% and lower E^PB^ values ranging from 2.0 to 13.4 J suggesting increased susceptibility to photoreduction.

In this second set, as quantum efficiencies were already notably high and E^PB^ values low, they were less affected by increasing η values, hinting at a saturation effect. The data displayed in Figure 9 thus support the idea that higher quantum efficiencies tend to correspond to lower photostability.

**Figure 10:**
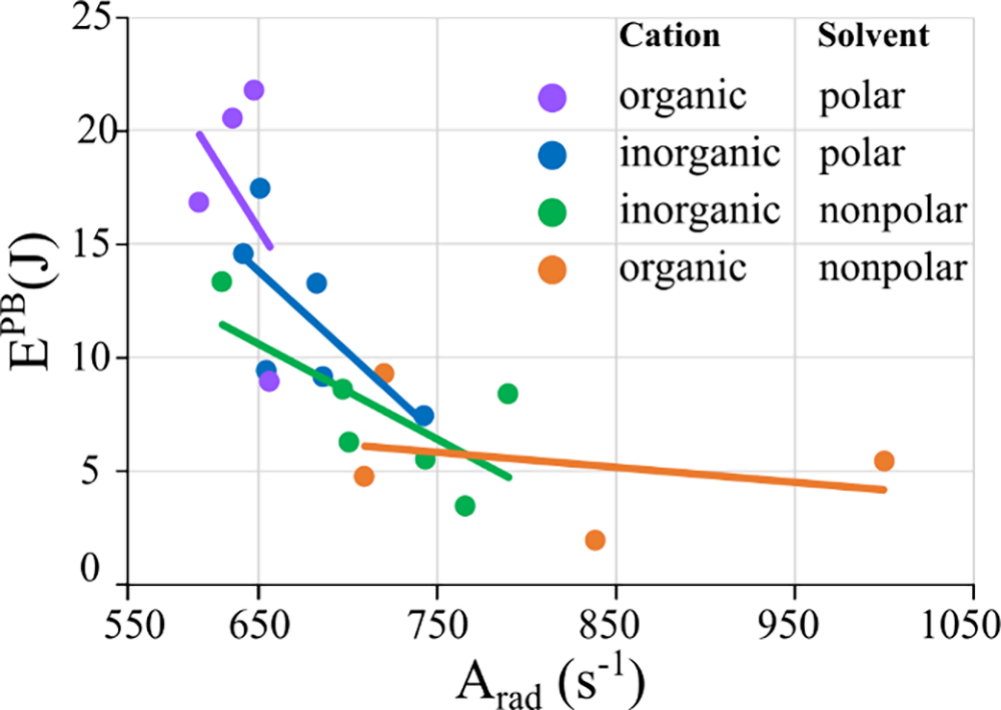
Photobleaching energy values (E^PB^) for all [Eu(BTFA)_4_]^−^ as a function of the radiative decay rate (A_rad_), distinguished by color for each combination of solvent and type of counterion. Polar solvents are acetone and acetonitrile, nonpolar solvents are dichloromethane and chloroform, inorganic counterions are Li^+^, Na^+^, K^+^ and organic counterions are C_5_mim^+^ and P_6,6,6,14_^+^. Regression lines were calculated for each of the four classes to highlight individual trends.

If electronic excitation indeed occurs before photoreduction and a greater number of complexes in the excited state indicate increased vulnerability to this phenomenon, then higher radiative decay rates (A_rad_) should correspond to lower values of E^PB^. Table 3 and Figure 10 illustrate this relationship using a scatter plot of E^PB^ against A_rad_ values for all complexes in the four solvents, highlighting this pattern. Figure 10 further shows that complexes with organic cations vary considerably in their E^PB^ values with varying solvent polarities, much more so than complexes with inorganic cations do. Hence, the photostability of complexes with organic cations is significantly enhanced by their dissolution in polar solvents.

**Figure 11:**
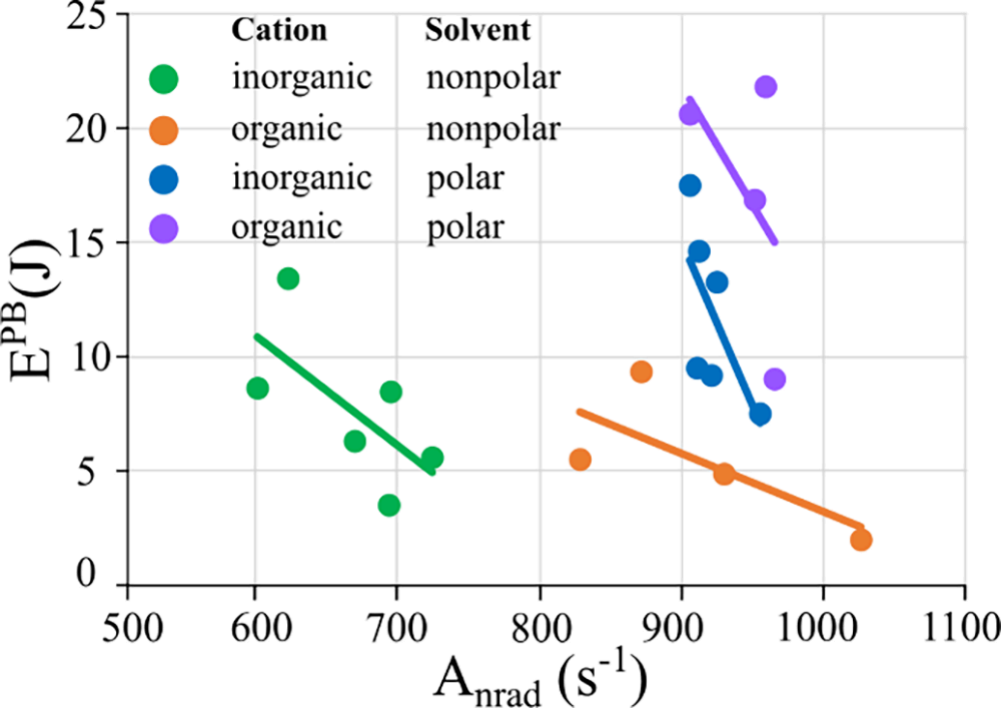
Photobleaching energy values (E^PB^) for all [Eu(BTFA)_4_]^−^ as a function of the nonradiative decay rate (A_nrad_), distinguished by color for each combination of solvent and type of counterion employed. Polar solvents are acetone and acetonitrile, nonpolar solvents are dichloromethane and chloroform, inorganic counterions are Li^+^, Na^+^, K^+^, and organic counterions are C_5_mim^+^ and P_6,6,6,14_^+^. Regression lines were calculated for each of the four classes to highlight individual trends.

Another factor that may contribute to the typically higher quantum efficiencies observed in nonpolar solvents is the reduction in their nonradiative decay rates (A_nrad_) (Table 4). This reduction could occur because of the inhibition of these nonradiative pathways caused by the separation of ion pairs by the solvent. To examine this possibility, let us make a scatter plot of pairs of E^PB^ and, this time, of A_nrad_ values (Figure 11).

For inorganic cations, which is indeed the case, their A_nrad_ values in nonpolar solvents (green dots) are smaller than those in polar solvents (blue dots), suggesting a connection between the reduced nonradiative pathways and solvent polarity for these species. In contrast, for organic cations, the E^PB^ values were strongly influenced by the solvent polarity (Figure 7), with minimal impact from the A_nrad_ variations (Figure 11).

These results indicate that lower quantum efficiencies with larger nonradiative decay rates provide protection against photobleaching. On the other hand, higher quantum efficiencies (greater η) that require ligand electronic excitation for the posterior transfer of energy to the europium ion also lead to increased susceptibility to photoreduction. This reinforces electronic excitation as a precursor to the subsequent photoreduction, where higher proportions of complexes in the excited state imply an increased propensity for this process.

In summary, the photostability of Eu complexes is significantly influenced by the solvent polarity and type of counterion. Inorganic cations are more stable in polar solvents because of their reduced nonradiative decay rates, whereas organic cations show greater variability, suggesting a more complex interplay with the solvent environment.

## Conclusions

Our study addressed the intricate photostability challenges encountered in europium [Eu(BTFA)_4_]^−^ β-diketonate complexes, shedding light on their photochemical, electrochemical, and luminescence behaviors, potentially offering valuable insights for diverse applications. By working directly in the energy domain, the evidence we unveiled unequivocally indicates that this photoreduction process is nonlinear in terms of the accumulated incident energy. Central to our investigation is the concept of photobleaching energy (E^PB^), which we have advanced as a parameter for comparing the photostabilities of lanthanide metal complexes: a convenient measure of their resistance to photobleaching.

Our findings pinpoint the irreversible photoreduction of Eu(III) to Eu(II) as the primary mechanism underlying photodegradation of these complexes. This conclusion is strongly supported by our electrochemical analyses, including cyclic voltammetry and chronoamperometry, which confirmed the reduction of Eu(III) to Eu(II) upon UV exposure.

The cyclic voltammetry experiments revealed the disappearance of the Eu^3+^/Eu^2+^ redox couple after photoreduction and the presence of free ligands and other species, providing direct evidence for the photoreduction process. Additionally, the emergence of the Eu(II) emission band following the electrochemical reduction further corroborates the transformation of Eu(III) to Eu(II).

The insights shared in this research suggest that there is a trade-off between the brightness of luminescence and the ability of the Eu complex to resist fading. In general, we observed that enhancing one may imply diminishing the other. Indeed, if a compound is highly luminescent, it tends to be less stable; conversely, if a complex is more photostable, it tends to be less luminescent.

Another important insight is that when the complex and cation are close together in nonpolar solvents, this situation creates coordination asymmetry that enhances luminescence but reduces photostability. Conversely, when the cation is farther away, the complex lies in a more symmetrical solvent envelope, resulting in decreased luminescence and heightened photostability. We also found that, with organic cations, transitioning from polar to nonpolar solvents significantly increases the radiative decay rate of luminescence (A_rad_). On the other hand, for inorganic alkaline metal cations, the most significant impact of such a solvent change is a reduction in the nonradiative decay rate (A_nrad_).

Therefore, depending on the intended application, these findings may provide a foundation for assessing and designing complexes with tunable stability under prolonged exposure to UV radiation. For example, if one intends to use a complex as a UV sensor, one should aim for complexes whose photostabilities are compatible with the amount of UV radiation expected to be detected. To detect smaller amounts of UV radiation, a highly unstable complex is desirable, such as P_6,6,6,14_[Eu(BTFA)_4_] in chloroform. Conversely, a more stable complex should be used to detect larger amounts of radiation, such as C_5_mim[Eu(BTFA)_4_], in acetone. Alternatively, a reasonable degree of photostability is required if a high luminescence is desired. In such cases, complexes with higher E^PB^ values should be targeted; with Eu(III) complexes with organic cations in polar solvents being potential candidates. Indeed, large organic cations have emerged as the most useful counterions in both cases, with solvents being the most important modulators of photostability. Finally, our findings offer a photochemical pathway for the preparation of Eu(II) complexes under UV illumination.

We hope that the insights from this article will provide guidance for regulating the photostability of europium complexes, thereby expanding their utility across various fields, from optoelectronics to sensing technologies.

As a follow-up to the present study, investigations of specific ligands designed to mitigate the photoreduction of lanthanide trivalent ions are currently being conducted in our laboratories.

## Experimental Section

### Reagents and Solvents

The reagents and solvents used were: 4,4,4-Trifluoro-1-phenyl-1,3-butanedione (HBTFA) 99% (Sigma-Aldrich), potassium hydroxide (85%) from Dinâmica, and ethanol from Honeywell 99.9% HPLC. Chloroform 99.9% HPLC, dichloromethane 99.9% HPLC, acetone 99.8% HPLC and acetonitrile 99.8% HPLC were purchased from Sigma-Aldrich. Deuterated solvents: Chloroform*-d* (99.8% D atoms) and Acetone*-d*_*6*_ (99.9% D atoms) were obtained from Sigma-Aldrich. The [EuCl_2_(H_2_O)_6_]Cl salt was prepared from europium oxide (Eu_2_O_3;_ Alfa Aesar, 99%).

### Characterizations and Equipment

All syntheses of the complexes were performed using a CEM Discover Microwave System for Chemical Synthesis 908005. The complexes were characterized by MALDI-TOF (mass spectrometry model Auto flex 3 Smart Beam Vertical spectrometer), infrared spectroscopy (KBr disk method and 4000–400 cm^–1^ spectral range in a Bruker model IFS 66 spectrophotometer), NMR experiments using an Agilent 400 MHz spectrometer, photostability experiments using a USB 4000 Ocean Optics spectrometer coupled with a Transilluminator Cross-linking (UVP), and melting point experiments on a BUCHI H-560 fusiometer. All the data for the synthesized compounds are included in the Supporting Information.

### NMR Spectroscopy

All NMR experiments were carried out on Agilent 400 MHz spectrometer, operating at 298 K, with resonance frequencies of 399.75 MHz for ^1^H, using a 5 mm NMR tube. NMR experiments were performed using two different solvents: chloroform*-d* and acetone*-d*_*6*_. Samples were prepared using standard and established procedures.

### Luminescence Spectroscopy

All luminescence experiments (excitation spectra, emission spectra, and lifetime curves) were performed using a Fluorolog-3 Horiba Jobin Yvon right-angle collection method with a Hamamatsu R928P photomultiplier, SPEX 1934 D phosphorimeter, 450 W Xe arc lamp, and pulsed 150 W Xe–Hg lamp at room temperature. A slit of 1 nm was used for the excitation and emission experiments. The data were obtained using a cuvette (optical length 1 cm) and a concentration of 1.00 × 10^–4^ M of the sample for each solvent: chloroform, dichloromethane, acetone, or acetonitrile. All excitation spectra, emission spectra, and lifetime curves are presented in the Supporting Information. All experiments for the five complexes were repeated in triplicates to assess the accuracy of our measurements. The experimental errors in the luminescence data were within a 90% confidence interval (details are presented in the Supporting Information). The maximum intensity wavelengths of the excitation spectra for the [C_5_mim][Eu(BTFA)_4_] ionic liquid complex in each solvent were λ= 366 nm (chloroform), λ= 366 nm (dichloromethane), λ= 363 nm (acetone), and λ= 366 nm (acetonitrile). For the ionic liquid complex P_6,6,6,14_[Eu(BTFA)_4_], the maximum intensity wavelengths in each solvent were λ= 369 nm (chloroform), λ= 368 nm (dichloromethane), λ= 368 nm (acetone), and λ= 365 nm (acetonitrile). Similarly, for the Li[Eu(BTFA)_4_] complex, the maximum intensity wavelengths of the excitation spectra for each solvent were λ= 361 nm (chloroform), λ= 360 nm (dichloromethane), λ= 361 nm (acetone), and λ= 364 nm (acetonitrile). For the Na[Eu(BTFA)_4_] complex, the maximum intensity wavelengths of the excitation spectra for each solvent were λ= 367 nm (benzene), λ= 366 nm (chloroform), λ= 362 nm (dichloromethane), λ= 365 nm (acetone), and λ= 365 nm (acetonitrile). For the K[Eu(BTFA)_4_] complex, the maximum intensity wavelengths of the excitation spectra in each solvent were 366 nm (chloroform), λ= 365 nm (dichloromethane), λ= 367 nm (acetone) and λ= 368 nm (acetonitrile). The lifetime decay curves were obtained by monitoring the ^5^D_0_ → ^7^F_2_ transition at the aforementioned excitation wavelengths. The experimental lifetime (τ_obs_) of the ^5^D_0_ level was determined by fitting luminescence decay curves to an exponential function. These τ_obs_ values are related to the radiative decay rates *A*_*rad*_ (or radiative rate constant *k*_*rad*_) and nonradiative decay rates *A*_*nrad*_ (or nonradiative rates *k*_*nrad*_) via eq 9.
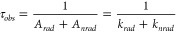
9

The value of A_rad_ can be determined using eq 10 below,^65^ where *I*_tot_ is the total intensity of the corrected emission spectrum, I_MD_ is the intensity corresponding to the magnetic dipole induced ^5^D_0_ → ^7^F_1_ transition, *n* is the refractive index of the solution (which can be replaced by the refractive index of the solvent in very dilute solutions), and A_MD,0_ = 14.65 s^–1^, is the spontaneous emission probability for the ^5^D_0_ → ^7^F_1_ transition in vacuum.^65^
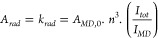
10

We can now determine the quantum efficiency η, which is also referred to by other schools, as the intrinsic metal-centered quantum yield for Eu(III) ϕ_*int*_^*Eu*^,^66^ according to eq 11 below.
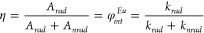
11

We employed the LUMPAC program to easily process and determine these photophysical properties based on the emission spectra and ^5^D_0_ lifetime values, according to the equations above and as detailed in the original LUMPAC article.^58^

### Procedure for Photobleaching Measurements

A UV DNA Crosslinker Transilluminator (UVP Inc.) was employed for the photobleaching measurements. This apparatus was equipped with five 8 W Hg lamps, serving as UV sources, precisely centered at 365 nm (320–400 nm) for the UVA range. Doses were digitally regulated using an integrated dosimeter. This system was connected through an optical fiber to a USB4000 spectrometer for real-time acquisition of luminescence spectral data during UV exposures. A total of 10 J/cm^2^ of accumulated energy were utilized, employing intervals of 0.5 J/cm^2^ for the acquisition of each emission spectrum, resulting in 20 consecutive accumulations for each sample. Solutions of luminescent complexes (20 samples, 2.50 mL each) were prepared at a concentration of 1 × 10^–4^ mol·L^–1^ for each of the following solvents: chloroform, dichloromethane, acetone, and acetonitrile. Each solution was then placed in a cuvette with a base area of 1 cm^2^.

### Electrochemical Experiments

Cyclic voltammetry (CV) experiments were carried out in a 3 mL V-cell containing three electrodes: vitreous carbon (Φ = 2.0 mm) as working electrode (WE), Pt wire as auxiliary electrode (AE), and Ag/AgCl, 3.0 mol·L^–1^ KCl as reference electrode (RE). The WE electrode was polished with an alumina slurry, rinsed with deionized water and acetone, and dried before use. An Autolab PGSTAT 128N (Metrohm) potentiostat/galvanostat with the NOVA software (2.1.5), was used for data acquisition and analysis. Acetonitrile was used as the solvent and tetra-*n*-butylammonium tetrafluorobotate (0.1 mol·L^–1^) was used as the supporting electrolyte. All experiments were performed by purging with an inert gas (argon) and conducted under inert atmosphere. CV experiments were carried out within the cathodic (−2.8 to 0.0 V) region at a scan rate of 100 mV·s^–1^. The controlled potential electrolysis (chronoamperometry) was carried out in the same V-cell at −1.5 V vs Ag/AgCl, using a vitreous carbon rod (area = 580 mm^2^) as cathode, and a Pt wire as anode placed in a separated compartment. Electrolysis was carried out until total luminescence quenching due to the total reduction of the P_6,6,6,14_[Eu^3+^(BTFA)_4_] complex, and the total charge passed was 12.5 coulombs.

### Microwave Synthetic Procedures

#### Microwave Procedure of the Complexes X[Eu(BTFA)_4_], X= Li^+^, Na^+^, or K^+^

First, deprotonation of the BTFA ligand (0.9 mmol HBTFA in 2 mL mL ethanol) was performed by dropping 1.5 mL of KOH (1.08 mmol) in ethanol into a microwave reactor tube. The tube was then placed in the reactor under the following conditions: 5 min at 35 °C and 100 W. In sequence, [EuCl_2_(H_2_O)_6_]Cl (0.18 mmol, 2 mL) was added to the deprotonated BTFA solution and allowed to react under the same conditions. The resulting yellowish solution was filtered, transferred to a beaker, and placed in a refrigerator for 1 h at 4 °C to allow further precipitation of KCl. In the next step, this reaction mixture was filtered to another beaker and left on the benchtop for slow evaporation of the solvent for 1 day. The obtained product was recrystallized in ethanol. The procedure described above for the K[Eu(BTFA)_4_] complex was also used to synthesize the other tetrakis complexes. For the Li[Eu(BTFA)_4_] complex, 1.5 mL of water was used to dissolve LiOH for the deprotonation of HBTFA in ethanolic solution. The characterization data for Na[Eu(BTFA)_4_] can be found in ref (53) and are crucial for understanding its properties. Additionally, the spectral data are included in the Supporting Information, providing comprehensive insights into the behavior of the compound.

Na[Eu(BTFA)_4_] (C_40_H_24_F_12_O_8_NaEu): white powder; reaction yield = 87%; melting point = 222–224 °C; elemental analysis calculated (found): C, 46.39% (47.25%); H, 2.34% (2.67%); FTIR (KBr disk): υ = 3069 cm^–1^ (=C–H, aromatic), υ= 1622 cm^–1^ (C = O), and υ= 1138 cm^–1^ (C–F). ^1^H NMR (400 MHz, (CD_3_)_2_CO δ ppm: 7.81 (d), 7.34 (t), 4.91 (s).

K[Eu(BTFA)_4_] (C_40_H_24_F_12_O_8_KEu): yellow powder; reaction yield = 86%; melting point = 211–213 °C; MALDI-TOF/MS [M/H]^+^ (*m*/*z*) calculated (found): 1051.56 g/mol (1051.05 g/mol); C, 45.68% (44.61%); H, 2.30% (2.56%); FTIR (KBr disk): υ = 3042 cm^–1^ (=C–H, aromatic), υ= 1616 cm^–1^ (C = O), and υ= 1136 cm^–1^ (C–F). ^1^H NMR (400 MHz, (CD_3_)_2_CO δ ppm: 7.85 (d), 7.36 (t) 5.19 (s).

Li[Eu(BTFA)_4_] (C_40_H_24_F_12_O_8_LiEu): white powder; reaction yield = 86%; melting point = 200–202 °C; FTIR (KBr disk): υ = 3065 cm^–1^ (=C–H, aromatic), υ= 1615 cm^–1^ (C = O), and υ= 1138 cm^–1^ (C–F).

#### Microwave Procedure of the Complexes P_6,6,6,14_[Eu(BTFA)_4_] and C_5_mim[Eu(BTFA)_4_]

First, deprotonation of the BTFA ligand (0.9 mmol HBTFA in 2 mL mL ethanol) was performed by dropping 1.5 mL of KOH (1.08 mmol) in ethanol into a microwave reactor tube. The tube was then placed in the reactor under the following conditions: 5 min at 35 °C and 100 W. In sequence, [EuCl_2_(H_2_O)_6_]Cl (0.18 mmol, 2 mL) was added to the deprotonated BTFA solution and allowed to react under the same conditions. When the reaction was completed, it was filtered and transferred directly to another tube containing a solution of ionic liquid of P_6,6,6,14_Cl or C_5_mimCl (0.18 mmol, 2 mL). The reaction mixture was then placed in a microwave reactor under the same conditions described above. The tube was left in a refrigerator for 1 h for further precipitation of KCl, followed by filtration and slow evaporation of the solvent over 1 day in a beaker. The obtained product was recrystallized from ethanol. The characterization data for [C_5_mim][Eu(BTFA)_4_] can be found in ref (53) and is crucial for understanding its properties. The spectral data are included in the Supporting Information, providing comprehensive insights into the behavior of the compound.

[C_5_mim][Eu(BTFA)_4_] (C_49_H_41_F_12_O_8_N_2_Eu): Yellow powder; Reaction yield = 89%; Melting point = 87–89 °C; MALDI-TOF/MS [M/H]^+^ (*m*/*z*) calculated (found): 1165.80 g/mol (1166.55 g/mol); Elemental analysis calculated (found): C, 50.48% (49.86%), H, 3.54% (3.48%), N, 2.40% (2.41%); FTIR (KBr disk): υ = 3157 cm-1 (C–H, CH_3_), υ = 3080 cm^–1^ (=C–H, aromatic), υ = 3147 cm^–1^ (C–H, CH_2_), υ= 1618 cm^–1^ (C = O), υ = 1361 cm^–1^ (C = N), υ= 1241 cm^–1^ (C–F). ^1^H NMR (400 MHz, (CHCl_3_) δ ppm: 7.36–8.07 (Ar), 20.34(s), 11.31(s), 10.22(s), 8.45 (s), 6.61(d), 4.66 (s), 3.23 (t), 2.39 (t), 1,31 (d).

[P_6,6,6,14_][Eu(BTFA)_4_] (C_72_H_92_F_12_O_8_PEu): Yellow powder; reaction yield = 85%; melting point = 80–91 °C; FTIR (KBr disk): υ = 3072 cm^–1^ (=C–H, aromatic), υ = 2964–2864 cm^–1^ (C–H, CH_3_, and CH_2_), υ= 1624 cm^–1^ (C = O), υ= 1181–1130 cm^–1^ (C–F). ^1^H NMR (400 MHz, (CHCl_3_) δ ppm: 7.58–7.23 (Ar), 5.07(s), 4,19(s), 2,37(s), 1.98(d), 1.52(d), 1.38(t) 1.23(s), 0.88(t).

## Supporting Information Available

Excitation spectra, emission spectra, lifetime curves, cyclic voltammograms, polynomial adjustments of the emission intensity as a function of the UVA energy, NMR spectra, IR spectra of the synthesized complexes (PDF)

## References

[ref1] Nehra K.; Dalal A.; Hooda A.; Bhagwan S.; Saini R. K.; Mari B.; Kumar S.; Singh D. Lanthanides β-Diketonate Complexes as Energy-Efficient Emissive Materials: A Review. J. Mol. Struct. 2022, 1249, 131531 10.1016/j.molstruc.2021.131531.

[ref2] Leite Silva C. M. B.; Bispo-Jr A. G.; Canisares F. S. M.; Castilho S. A.; Lima S. A. M.; Pires A. M. Eu^3+^ -tetrakis Β-diketonate Complexes for Solid-state Lighting Application. Luminescence 2019, 34 (8), 877–886. 10.1002/bio.3686. 31347269

[ref3] Essahili O.; El Azzaoui A.; Ilsouk M.; Moudam O. Investigating the Long-Term Stability of Photoluminescence Lifetimes in PMMA Films Doped with β-Diketonate Europium Complexes Based on Bipyridine and Terpyridine Derivatives. J. Photochem. Photobiol. A Chem. 2024, 447, 115211 10.1016/j.jphotochem.2023.115211.

[ref4] Kai J.; Felinto M. C. F. C.; Nunes L. A. O.; Malta O. L.; Brito H. F. Intermolecular Energy Transfer and Photostability of Luminescence-Tuneable Multicolour PMMA Films Doped with Lanthanide−β-Diketonate Complexes. J. Mater. Chem. 2011, 21 (11), 3796 10.1039/c0jm03474f.

[ref5] Wen T.; Zhang W.; Hu X.; He L.; Li H. Insight into the Luminescence Behavior of Europium(III) Β-Diketonate Complexes Encapsulated in Zeolite L Crystals. ChemPlusChem. 2013, 78 (5), 438–442. 10.1002/cplu.201300073.

[ref6] Ding Y.; Wang Y.; Li H.; Duan Z.; Zhang H.; Zheng Y. Photostable and Efficient Red-Emitters Based on Zeolite L Crystals. J. Mater. Chem. 2011, 21 (38), 14755 10.1039/c1jm12282g.

[ref7] Lima P. P.; Paz F. A. A.; Brites C. D. S.; Quirino W. G.; Legnani C.; Costa e Silva M.; Ferreira R. A. S.; Júnior S. A.; Malta O. L.; Cremona M.; Carlos L. D. White OLED Based on a Temperature Sensitive Eu^3+^/Tb^3+^ β-Diketonate Complex. Org. Electron 2014, 15 (3), 798–808. 10.1016/j.orgel.2014.01.009.

[ref8] Zinna F.; Pasini M.; Cabras M.; Scavia G.; Botta C.; Di Bari L.; Giovanella U. Impact of Chiral Ligands on Photophysical and Electro-optical Properties of Β-diketonate Europium Complexes in Circularly Polarized OLEDs. Chirality 2023, 35 (5), 270–280. 10.1002/chir.23538. 36847610

[ref9] Chen C.; Corry B.; Huang L.; Hildebrandt N. FRET-Modulated Multihybrid Nanoparticles for Brightness-Equalized Single-Wavelength Barcoding. J. Am. Chem. Soc. 2019, 141 (28), 11123–11141. 10.1021/jacs.9b03383. 31251609

[ref10] Zhang G.; Cui J.; Zhang H.; Yang J.; Zhang H.; Han H.; Wang G. A Series of Carbonate-Brisdged Ln (Ln = Eu, Tb, Gd) Frameworks: Colour Tunability for Barcode Applications and Selective Luminescence Sensing towards Nitroimidazole Antibiotics. Inorg. Chem. Commun. 2022, 137, 109173 10.1016/j.inoche.2021.109173.

[ref11] Kaczmarek A. M.; Liu Y.; Wang C.; Laforce B.; Vincze L.; Van Der Voort P.; Van Hecke K.; Van Deun R. Lanthanide “Chameleon” Multistage Anti-Counterfeit Materials. Adv. Funct. Mater. 2017, 27 (20), 1700258 10.1002/adfm.201700258.

[ref12] Karachousos-Spiliotakopoulos K.; Tangoulis V.; Panagiotou N.; Tasiopoulos A.; Nastopoulos V.; Moreno-Pineda E.; Wernsdorfer W.; Schulze M.; Botas A. M. P.; Carlos L. D. Lanthanide Luminescence Thermometry and Slow Magnetic Relaxation in 3-D Polycyanidometallate-Based Materials. Inorg. Chem. 2022, 61 (46), 18629–18639. 10.1021/acs.inorgchem.2c03128. 36345918

[ref13] Parker D.; Fradgley J. D.; Wong K.-L. The Design of Responsive Luminescent Lanthanide Probes and Sensors. Chem. Soc. Rev. 2021, 50 (14), 8193–8213. 10.1039/D1CS00310K. 34075982

[ref14] Wheeler S.; Breen C.; Li Y.; Hewitt S. H.; Robertson E.; Yates E. A.; Barsukov I. L.; Fernig D. G.; Butler S. J. Anion Binding to a Cationic Europium(iii) Probe Enables the First Real-Time Assay of Heparan Sulfotransferase Activity. Org. Biomol Chem. 2022, 20 (3), 596–605. 10.1039/D1OB02071D. 34951618 PMC8767414

[ref15] Gorai T.; Schmitt W.; Gunnlaugsson T. Highlights of the Development and Application of Luminescent Lanthanide Based Coordination Polymers, MOFs and Functional Nanomaterials. Dalton Transactions 2021, 50 (3), 770–784. 10.1039/D0DT03684F. 33351011

[ref16] Viana R. da S.; Costa L. A. de M.; Harmon A. C.; Gomes Filho M. A.; Falcão E. H. L.; Vicente M. G. H.; Junior S. A.; Mathis J. M. ^177^ Lu-Labeled Eu-Doped Mesoporous SiO _2_ Nanoparticles as a Theranostic Radiopharmaceutical for Colorectal Cancer. ACS Appl. Nano Mater. 2020, 3 (9), 8691–8701. 10.1021/acsanm.0c01427.

[ref17] Wang Y.; Zhao G.; Chi H.; Yang S.; Niu Q.; Wu D.; Cao W.; Li T.; Ma H.; Wei Q. Self-Luminescent Lanthanide Metal–Organic Frameworks as Signal Probes in Electrochemiluminescence Immunoassay. J. Am. Chem. Soc. 2021, 143 (1), 504–512. 10.1021/jacs.0c12449. 33370533

[ref18] Mathieu E.; Sipos A.; Demeyere E.; Phipps D.; Sakaveli D.; Borbas K. E. Lanthanide-Based Tools for the Investigation of Cellular Environments. Chem. Commun. 2018, 54 (72), 10021–10035. 10.1039/C8CC05271A. 30101249

[ref19] Fu L.; Wen X.; Ai X.; Sun Y.; Wu Y.; Zhang J.; Wang Y. Efficient Two-Photon-Sensitized Luminescence of a Europium(III) Complex. Angew. Chem., Int. Ed. 2005, 44 (5), 747–750. 10.1002/anie.200462382. 15602752

[ref20] Quirino W.; Reyes R.; Legnani C.; Nóbrega P. C.; Santa-Cruz P. A.; Cremona M. Eu-β-Diketonate Complex OLED as UV Portable Dosimeter. Synth. Met. 2011, 161 (11–12), 964–968. 10.1016/j.synthmet.2011.03.001.

[ref21] Sousa F. L. N.; Mojica-Sánchez L. C.; Gavazza S.; Florencio L.; Vaz E. C. R.; Santa-Cruz P. A. Printable UV Personal Dosimeter: Sensitivity as a Function of DoD Parameters and Number of Layers of a Functional Photonic Ink. Mater. Res. Express 2016, 3 (4), 045701 10.1088/2053-1591/3/4/045701.

[ref22] Cavalcanti Rodrigues Vaz E.; Domingues T.; Cavalcante Santos T. E.; Moura L.; Tavares T.; Melo L. F.; Henriques D.; De Barros Melo S.; Santa-Cruz P. Personal Monitoring of Cutaneous Vitamin D3 Production through a Printable UV Molecular Dosimeter. Braz. J. Radiat. Sci. 2022, 10 (2A), 16 10.15392/bjrs.v10i2A.2032.

[ref23] Castelletto S.; Boretti A. Luminescence Solar Concentrators: A Technology Update. Nano Energy 2023, 109, 108269 10.1016/j.nanoen.2023.108269.

[ref24] Lima P. P.; Nolasco M. M.; Paz F. A. A.; Ferreira R. A. S.; Longo R. L.; Malta O. L.; Carlos L. D. Photo–Click Chemistry to Design Highly Efficient Lanthanide β-Diketonate Complexes Stable under UV Irradiation. Chem. Mater. 2013, 25 (4), 586–598. 10.1021/cm303776x.

[ref25] Kovacs D.; Borbas K. E. The Role of Photoinduced Electron Transfer in the Quenching of Sensitized Europium Emission. Coord. Chem. Rev. 2018, 364, 1–9. 10.1016/j.ccr.2018.03.004.

[ref26] Kovacs D.; Mathieu E.; Kiraev S. R.; Wells J. A. L.; Demeyere E.; Sipos A.; Borbas K. E. Coordination Environment-Controlled Photoinduced Electron Transfer Quenching in Luminescent Europium Complexes. J. Am. Chem. Soc. 2020, 142 (30), 13190–13200. 10.1021/jacs.0c05518. 32623881

[ref27] Wei H.; Zhao Z.; Wei C.; Yu G.; Liu Z.; Zhang B.; Bian J.; Bian Z.; Huang C. Antiphotobleaching: A Type of Structurally Rigid Chromophore Ready for Constructing Highly Luminescent and Highly Photostable Europium Complexes. Adv. Funct Mater. 2016, 26 (13), 2085–2096. 10.1002/adfm.201505040.

[ref28] Dar W. A.; Iftikhar K. Phase Controlled Colour Tuning of Samarium and Europium Complexes and Excellent Photostability of Their PVA Encapsulated Materials. Structural Elucidation, Photophysical Parameters and the Energy Transfer Mechanism in the Eu ^3+^ Complex by Sparkle/PM3 Calculations. Dalton Transactions 2016, 45 (21), 8956–8971. 10.1039/C6DT00549G. 27157414

[ref29] Zheng W.; Li S.-J.; Li C.-H.; Zheng Y.-X.; You X.-Z. Dramatic Improvement in Photostability of Luminescent Eu(III) Complexes with Tetraphenylimidodiphosphinate Ligand. J. Lumin. 2014, 146, 544–549. 10.1016/j.jlumin.2013.10.051.

[ref30] Wu J.; Feng J. A Multifunctional Luminescent Europium Photostabilizer Based on a Novel Hindered Amine Ligand and Phenanthroline. J. Appl. Polym. Sci. 2013, 130 (2), 1399–1405. 10.1002/app.39325.

[ref31] Del Rosso T.; Zaman Q.; Cremona M.; Pandoli O.; Barreto A. R. J. SPR Sensors for Monitoring the Degradation Processes of Eu(Dbm)_3_(Phen) and Alq3 Thin Films under Atmospheric and UVA Exposure. Appl. Surf. Sci. 2018, 442, 759–766. 10.1016/j.apsusc.2018.02.219.

[ref32] Gameiro C. G.; da Silva E. F.; Alves S.; de Sá G. F.; Santa-Cruz P. A. Lanthanide Complexes Dispersed in Enamel: A Promising New Material for Photonic Devices. J. Alloys Compd. 2001, 323–324, 820–823. 10.1016/S0925-8388(01)01152-5.

[ref33] Wei C.; Wei H.; Yan W.; Zhao Z.; Cai Z.; Sun B.; Meng Z.; Liu Z.; Bian Z.; Huang C. Water-Soluble and Highly Luminescent Europium(III) Complexes with Favorable Photostability and Sensitive PH Response Behavior. Inorg. Chem. 2016, 55 (20), 10645–10653. 10.1021/acs.inorgchem.6b01897. 27668968

[ref34] Emelina T.; Mirochnik A.; Kalinovskaya I. Photostability of Luminescent Europium(III) Hexafluoroacetylacetonates: Combined Theoretical and Experimental Study. J. Lumin. 2021, 238, 118274 10.1016/j.jlumin.2021.118274.

[ref35] Lapaev D. V.; Nikiforov V. G.; Lobkov V. S.; Knyazev A. A.; Galyametdinov Y. G. A Reusable and Self-Recoverable Vitrified Film of an Anisometric Europium(III) β-Diketonate Complex with UV Light-Responsive Eu3+ Emission. J. Photochem. Photobiol. A Chem. 2022, 427, 113821 10.1016/j.jphotochem.2022.113821.

[ref36] Knyazev A. A.; Karyakin M. E.; Krupin A. S.; Galyametdinov Y. G. Influence of β-Diketone Structure on Optical Properties of Formed by Eu(III) Adducts Photostable Transparent Films with Effective Luminescence. Dyes Pigm. 2022, 201, 110233 10.1016/j.dyepig.2022.110233.

[ref37] Hasan N.; Iftikhar K. Syntheses, Crystal Structure and Photophysical Properties of [Sm(Dbm)_3_(Impy)] and [Tb(Dbm)_3_(Impy)] and Their Hybrid Films. New J. Chem. 2019, 43 (11), 4391–4405. 10.1039/C8NJ05045G.

[ref38] Draper J. W. XLIX. Description of the Tithonometer, an Instrument for Measuring the Chemical Force of the Indigo-Tithonic Rays. London, Edinburgh, and Dublin Philosophical Magazine and Journal of Science 1843, 23 (154), 401–415. 10.1080/14786444308644763.

[ref39] Basal L. A.; Allen M. J. Synthesis, Characterization, and Handling of EuII-Containing Complexes for Molecular Imaging Applications. Front. Chem. 2018, 6, 65 10.3389/fchem.2018.00065. 29616213 PMC5867344

[ref40] Lewandowski E. C.; Arban C. B.; Deal M. P.; Batchev A. L.; Allen M. J. Europium(II/III) Coordination Chemistry toward Applications. Chem. Commun. 2024, 60 (77), 10655–10671. 10.1039/D4CC03080J. PMC11373536 39230388

[ref41] Melo L. L. L. S.; Castro G. P.; Gonçalves S. M. C. Substantial Intensification of the Quantum Yield of Samarium(III) Complexes by Mixing Ligands: Microwave-Assisted Synthesis and Luminescence Properties. Inorg. Chem. 2019, 58 (5), 3265–3270. 10.1021/acs.inorgchem.8b03340. 30775912

[ref42] Jiang J.; Higashiyama N.; Machida K.; Adachi G. The Luminescent Properties of Divalent Europium Complexes of Crown Ethers and Cryptands. Coord. Chem. Rev. 1998, 170 (1), 1–29. 10.1016/S0010-8545(98)00070-8.

[ref43] Tsuboi H.; Soga K.; Inoue H.; Makishima A. Synthesis and Fluorescence Properties of Eu ^2+^ -Complex-Doped SiO _2_ Gels. J. Am. Ceram. Soc. 1998, 81 (5), 1197–1202. 10.1111/j.1151-2916.1998.tb02468.x.

[ref44] Qiu J.; Miura K.; Sugimoto N.; Hirao K. Preparation and Fluorescence Properties of Fluoroaluminate Glasses Containing Eu2+ Ions. J. Non Cryst. Solids 1997, 213-214, 266–270. 10.1016/S0022-3093(97)00011-2.

[ref45] Wei Y.; Cao L.; Lv L.; Li G.; Hao J.; Gao J.; Su C.; Lin C. C.; Jang H. S.; Dang P.; Lin J. Highly Efficient Blue Emission and Superior Thermal Stability of BaAl_12_O_19_:Eu^2+^ Phosphors Based on Highly Symmetric Crystal Structure. Chem. Mater. 2018, 30 (7), 2389–2399. 10.1021/acs.chemmater.8b00464.

[ref46] Qiao J.; Ning L.; Molokeev M. S.; Chuang Y.-C.; Liu Q.; Xia Z. Eu ^2+^ Site Preferences in the Mixed Cation K _2_ BaCa(PO_4_)_2_ and Thermally Stable Luminescence. J. Am. Chem. Soc. 2018, 140 (30), 9730–9736. 10.1021/jacs.8b06021. 29985612

[ref47] Ottolenghi M.; Stein G. The Radiation Chemistry of Chloroform. Radiat. Res. 1961, 14 (3), 281 10.2307/3570922. 13731687

[ref48] Yabuta R.; Kobayashi N.; Nakamura K. Electrochemically Regulated Luminescence of Europium Complexes with β-Diketone in Polyether Matrices. Phys. Chem. Chem. Phys. 2023, 25 (38), 25979–25984. 10.1039/D3CP02283H. 37581218

[ref49] Burnett M. E.; Adebesin B.; Funk A. M.; Kovacs Z.; Sherry A. D.; Ekanger L. A.; Allen M. J.; Green K. N.; Ratnakar S. J. Electrochemical Investigation of the Eu3+/2+ Redox Couple in Complexes with Variable Numbers of Glycinamide and Acetate Pendant Arms. Eur. J. Inorg. Chem. 2017, 2017 (43), 5001–5005. 10.1002/ejic.201701070. 29403330 PMC5795619

[ref50] Wang X.; Zhou S.; Wu L. Stability, UV Shielding Properties, and Light Conversion Behavior of Eu(BMDM)_3_@polysiloxane Nanoparticles in Water and Polyurethane Films. Mater. Chem. Phys. 2012, 137 (2), 644–651. 10.1016/j.matchemphys.2012.09.070.

[ref51] Bolton J. R. A Master Equation for Photochemical Rates. Photochem. Photobiol. 2020, 96 (6), 1355–1357. 10.1111/php.13325. 32866292

[ref52] Tver’yanovich Yu. S. Concentration Quenching of Luminescence of Rare-Earth Ions in Chalcogenide Glasses. Glass Physics and Chemistry 2003, 29 (2), 166–168. 10.1023/A:1023407125519.

[ref53] Tamaki S.; Hasegawa Y.; Yajima H. Factors Influencing the Luminescence Intensity of Europium(III) Complexes Prepared via Synergistic Extraction. Talanta 2013, 105, 262–266. 10.1016/j.talanta.2012.11.075. 23598017

[ref54] Fang X.; Song H.; Xie L.; Liu Q.; Zhang H.; Bai X.; Dong B.; Wang Y.; Han W. Origin of Luminescence Enhancement and Quenching of Europium Complex in Solution Phase Containing Ag Nanoparticles. J. Chem. Phys. 2009, 131 (5), 054506 10.1063/1.3193721. 19673573

[ref55] Berglund A. J. Nonexponential Statistics of Fluorescence Photobleaching. J. Chem. Phys. 2004, 121 (7), 2899–2903. 10.1063/1.1773162. 15291600

[ref56] Lin J.-T. Optimal Efficacy in Light-Activated Biomedical Systems and Nonlinear Laws versus Linear Beer-Lambert Law and Bunsen-Roscoe Reciprocal Law. Open Access J. Biomed. Eng. Biosci. 2018, 1 (5), 114 10.32474/OAJBEB.2018.01.000123.

[ref57] Bunsen R. W.; Roscoe H. E. III. Photochemical Researches.—Part V. On the Measurement of the Chemical Action of Direct and Diffuse Sunlight. Proc. R. Soc. London 1863, 12, 306–312. 10.1098/rspl.1862.0069.

[ref58] Dutra J. D. L.; Bispo T. D.; Freire R. O. LUMPAC Lanthanide Luminescence Software: Efficient and User Friendly. J. Comput. Chem. 2014, 35 (10), 772–775. 10.1002/jcc.23542. 24532191

[ref59] Castro G. P.; Melo L. L. L. S.; Hallwass F.; Gonçalves S. M. C.; Simas A. M. NMR and Luminescence Experiments Reveal the Structure and Symmetry Adaptation of a Europium Ionic Liquid to Solvent Polarity. Dalton Transactions 2021, 50 (29), 10193–10205. 10.1039/D1DT01050F. 34231624

[ref60] Böhm A.; Bach T. Radical Reactions Induced by Visible Light in Dichloromethane Solutions of Hünig’s Base: Synthetic Applications and Mechanistic Observations. *Chemistry – A*. European Journal 2016, 22 (44), 15921–15928. 10.1002/chem.201603303. 27628907

[ref61] Zeldes H.; Livingston R. Paramagnetic Resonance Study of Liquids during Photolysis. II. Acetone and Solutions Containing Acetone. J. Chem. Phys. 1966, 45 (6), 1946–1954. 10.1063/1.1727877.

[ref62] Khudyakova I. V.; Levin P. P. Photogeneration of Organic Free Radicals in Liquid Solutions. Mini Rev. Org. Chem. 2021, 18 (7), 830–835. 10.2174/1570193X17999200816133108.

[ref63] Nekipelova T. D.; Kasparov V. V.; Kovarskii A. L.; Vorobiev A. Kh.; Podrugina T. A.; Vinogradov D. S.; Kuzmin V. A.; Zefirov N. S. Free Radicals in Photolysis of Mixed Phosphonium–Iodonium Ylides and in Their Reactions with Acetylenes. Doklady Physical Chemistry 2017, 474 (2), 109–113. 10.1134/S0012501617060070.

[ref64] Lima N. B. D.; Gonçalves S. M. C.; Júnior S. A.; Simas A. M. A Comprehensive Strategy to Boost the Quantum Yield of Luminescence of Europium Complexes. Sci. Rep 2013, 3 (1), 2395 10.1038/srep02395. 23928866 PMC3738935

[ref65] Werts M. H. V.; Jukes R. T. F.; Verhoeven J. W. The Emission Spectrum and the Radiative Lifetime of Eu^3+^ in Luminescent Lanthanide Complexes. Phys. Chem. Chem. Phys. 2002, 4 (9), 1542–1548. 10.1039/b107770h.

[ref66] Wong K.-L.; Bünzli J.-C. G.; Tanner P. A. Quantum Yield and Brightness. J. Lumin. 2020, 224, 117256 10.1016/j.jlumin.2020.117256.

