# Supplementary material for: Unmasking the UV Photobleaching of β-Diketonate [Eu(BTFA)4]− Complexes as an Energy-Driven Photoreduction Process
Source: Inorg Chem. 2025 Feb 18;64(8):3842–56. doi: 10.1021/acs.inorgchem.4c05014 (PMC11881040; doi:10.1021/acs.inorgchem.4c05014)
Supplement: Supplementary file 1 — ic4c05014_si_001.pdf [file ic4c05014_si_001.pdf]

Electronic Supplementary Information

Unmasking the UV Photobleaching of β-Diketonate $[Eu(BTFA)_4]^-$ Complexes as an Energy-Driven Photoreduction Process

Lizandra L. L. S. Melo, Gerson P. Castro Jr., Marcelo Navarro, Simone M. C. Gonçalves, and Alfredo M. Simas*

*e-mail: simas@ufpe.br

Departamento de Química Fundamental, CCEN,

Universidade Federal de Pernambuco,

50670-901, Recife, Pernambuco

Brazil

Table of Contents

Table of Contents 2

Table 3

List of Figures 3

1. Luminescence 10

   1.1 Excitation Spectra 10

   1.2 Emission Spectra 20

   1.3 Luminescence time decay profiles 33

2.Cyclic voltammograms 44

3. Photostability 49

   3.1. Polynomial adjustments of the emission intensity as a function of the UVA energy 49

4. NMR Data 59

5. Infrared Spectra 61

Table

Table S 1. Luminescence data for all 1x10^{-4} M solution of $[Eu(BTFA)_4]^-$ complexes in the four solvents studied. Lifetimes, τ; radiative decay rates, A_{rad}; nonradiative decay rates, A_{nrad}; quantum efficiency, η; luminescence data experimental errors within a 90% confidence interval are: τ_{obs}, 4% ; A^{rad} 3% ; A_{nrad} 8% ; η, 5%; of the reported values. 43

List of Figures

Figure S 1: Excitation spectrum of a 1x10^{-4} M solution of a 1x10^{-4} M solution of $Li[Eu(BTFA)_4]$ complex in $CHCl_3$ with a maximum excitation wavelength of λ= 361 nm. 10

Figure S 2: Excitation spectrum of a 1x10^{-4} M solution of a 1x10^{-4}M solution of $Li[Eu(BTFA)_4]$ complex in CH_2Cl_2 with a maximum excitation wavelength of λ= 360 nm. 10

Figure S 3: Excitation spectrum of a 1x10^{-4} M solution of $Li[Eu(BTFA)_4]$ complex in $(CH_3)_2CO$ with a maximum excitation wavelength of λ= 361 nm. 11

Figure S 4: Excitation spectrum of a 1x10^{-4} M solution of a 1x10^{-4}M solution of $Li[Eu(BTFA)_4]$ complex in CH_3CN with a maximum excitation wavelength of λ= 364 nm. 11

Figure S 5: Excitation spectrum of a 1x10^{-4} M solution of $Na[Eu(BTFA)_4]$ complex in $CHCl_3$ with a maximum excitation wavelength of λ= 365 nm. 12

Figure S 6: Excitation spectrum of a 1x10^{-4} M solution of $Na[Eu(BTFA)_4]$ complex in CH_2Cl_2 with a maximum excitation wavelength of λ= 362 nm. 12

Figure S 7: Excitation spectrum of a 1x10^{-4} M solution of $Na[Eu(BTFA)_4]$ complex in $(CH_3)_2CO$ with a maximum excitation wavelength of λ= 368 nm. 13

Figure S 8: Excitation spectrum of a 1x10^{-4} M solution of $Na[Eu(BTFA)_4]$ complex in CH_3CN with a maximum excitation wavelength of λ= 367 nm. 13

Figure S 9: Excitation spectrum of a 1x10^{-4} M solution of $K[Eu(BTFA)_4]$ complex in $CHCl_3$ with a maximum excitation wavelength of λ= 366 nm. 14

Figure S 10: Excitation spectrum of a 1x10^{-4} M solution of $K[Eu(BTFA)_4]$ complex in CH_2Cl_2 with a maximum excitation wavelength of λ= 365 nm. 14

Figure S 11: Excitation spectrum of a 1x10^{-4} M solution of $K[Eu(BTFA)_4]$ complex in $(CH_3)_2CO$ with a maximum excitation wavelength of λ= 367 nm. 15

Figure S 12: Excitation spectrum of a 1x10^{-4} M solution of $K[Eu(BTFA)_4]$ complex in CH_3CN with a maximum excitation wavelength of λ= 368 nm. 15

Figure S 13: Excitation spectrum of a 1x10^{-4} M solution of $C_5mim[Eu(BTFA)_4]$ complex in $CHCl_3$ with a maximum excitation wavelength of λ= 366 nm. 16

Figure S 14: Excitation spectrum of a $1x10^{-4}$ M solution of $C_5mim[Eu(BTFA)_4]$ complex in CH_2Cl_2 with a maximum excitation wavelength of λ= 366 nm. 16

Figure S 15: Excitation spectrum of a $1x10^{-4}$ M solution of $C_5mim[Eu(BTFA)_4]$ complex in $(CH_3)_2CO$ with a maximum excitation wavelength of λ= 363 nm. 17

Figure S 16: Excitation spectrum of a $1x10^{-4}$ M solution of $C_5mim[Eu(BTFA)_4]$ complex in CH_3CN with a maximum excitation wavelength of λ= 366 nm. 17

Figure S 17: Excitation spectrum of a $1x10^{-4}$ M solution of $P_{6,6,6,14}[Eu(BTFA)_4]$ complex in $CHCl_3$ with a maximum excitation wavelength of λ= 369 nm. 18

Figure S 18: Excitation spectrum of a $1x10^{-4}$ M solution of $P_{6,6,6,14}[Eu(BTFA)_4]$ complex in CH_2Cl_2 with a maximum excitation wavelength of λ= 368 nm. 18

Figure S 19: Excitation spectrum of a $1x10^{-4}$ M solution of $P_{6,6,6,14}[Eu(BTFA)_4]$ complex in $(CH_3)_2CO$ with a maximum excitation wavelength of λ= 368 nm. 19

Figure S 20: Excitation spectrum of a $1x10^{-4}$ M solution of $P_{6,6,6,14}[Eu(BTFA)_4]$ complex in CH_3CN with a maximum excitation wavelength of λ= 367 nm 19

Figure S 21: Emission spectrum of a $1x10^{-4}$ M solution of $Li[Eu(BTFA)_4]$ complex in $CHCl_3$ with a maximum emission wavelength of λ= 609 nm. 20

Figure S 22: Emission spectrum of a $1x10^{-4}$ M solution of $Li[Eu(BTFA)_4]$ complex in CH_2Cl_2 with a maximum emission wavelength of λ= 609 nm 20

Figure S 23: Emission spectrum of a $1x10^{-4}$ M solution of $Li[Eu(BTFA)_4]$ complex in $(CH_3)_2CO$ with a maximum emission wavelength of λ= 610 nm 21

Figure S 24: Emission spectrum of a $1x10^{-4}$ M solution of $Li[Eu(BTFA)_4]$ complex in CH_3CN with a maximum emission wavelength of λ= 610 nm. 21

Figure S 25: Emission spectrum of a $1x10^{-4}$ M solution of $Na[Eu(BTFA)_4]$ complex in $CHCl_3$ with a maximum emission wavelength of λ= 609 nm. 22

Figure S 26: Emission spectrum of a $1x10^{-4}$ M solution of $Na[Eu(BTFA)_4]$ complex in CH_2Cl_2 with a maximum emission wavelength of λ= 609 nm. 22

Figure S 27: Emission spectrum of a $1x10^{-4}$ M solution of $Na[Eu(BTFA)_4]$ complex in $(CH_3)_2CO$ with a maximum emission wavelength of λ= 610 nm. 23

Figure S 28: Emission spectrum of a $1x10^{-4}$ M solution of $Na[Eu(BTFA)_4]$ complex in CH_3CN with a maximum emission wavelength of λ= 610 nm. 23

Figure S 29: Emission spectrum of a $1x10^{-4}$ M solution of $K[Eu(BTFA)_4]$ complex in $CHCl_3$ with a maximum emission wavelength of λ= 609 nm. 24

Figure S 30: Emission spectrum of a $1x10^{-4}$ M solution of $K[Eu(BTFA)_4]$ complex in CH_2Cl_2 with a maximum emission wavelength of λ= 609 nm. 24

Figure S 31: Emission spectrum of a $1x10^{-4}$ M solution of $K[Eu(BTFA)_4]$ complex in $(CH_3)_2CO$ with a maximum emission wavelength of λ= 610 nm. 25

Figure S 32: Emission spectrum of a $1x10^{-4}$ M solution of $K[Eu(BTFA)_4]$ complex in CH_3CN with a maximum emission wavelength of λ= 610 nm 25

Figure S 33: Emission spectrum of a $1x10^{-4}$ M solution of $C_5mim[Eu(BTFA)_4]$ complex in $CHCl_3$ with a maximum emission wavelength of λ= 611 nm. 26

Figure S 34: Emission spectrum of a $1x10^{-4}$M solution of $C_5mim[Eu(BTFA)_4]$ complex in CH_2Cl_2 with a maximum emission wavelength of λ= 611 nm. 26

Figure S 35: Emission spectrum of a $1x10^{-4}$ M solution of $C_5mim[Eu(BTFA)_4]$ complex in $(CH_3)_2CO$ with a maximum emission wavelength of λ= 611 nm. 27

Figure S 36: Emission spectrum of a $1x10^{-4}$ M solution of $C_5mim[Eu(BTFA)_4]$ complex in CH_3CN with a maximum emission wavelength of λ= 611 nm. 27

Figure S 37: Emission spectrum of a $1x10^{-4}$ M solution of $P_{6,6,6,14}[Eu(BTFA)_4]$ complex in $CHCl_3$ with a maximum emission wavelength of λ= 610 nm. 28

Figure S 38: Emission spectrum of a $1x10^{-4}$ M solution of $P_{6,6,6,14}[Eu(BTFA)_4]$ complex in CH_2Cl_2 with a maximum emission wavelength of λ= 610 nm. 28

Figure S 39: Emission spectrum of a $1x10^{-4}$ M solution of $P_{6,6,6,14}[Eu(BTFA)_4]$ complex in $(CH_3)_2CO$ with a maximum emission wavelength of λ= 610 nm. 29

Figure S 40: Emission spectrum of a $1x10^{-4}$ M solution of $P_{6,6,6,14}[Eu(BTFA)_4]$ complex in CH_3CN with a maximum emission wavelength of λ= 610 nm. 29

Figure S 41: Emission spectra of a $1x10^{-4}$ M solution of $P_{6,6,6,14}[Gd(BTFA)_4]$ complex in chloroform, recorded before [(a) and (c)], and after UVA irradiation [(b) and (d)], from an excitation wavelength of 370 nm. The spectra show no signals in the Eu(II) emission region (400–500 nm). The intensity units are arbitrary but consistent across all four graphs and identical to those in the subsequent experiments, which depict similar conditions for $P_{6,6,6,14}[Eu(BTFA)_4]$ for comparison, with the scales in panels (c) and (d) also matching those in the subsequent experiments to emphasize the absence of any spectral changes in the range of 400–700 nm after UVA exposure. 30

Figure S 42: Emission spectra of a $1x10^{-4}$M solution of $Li[Eu(BTFA)_4]$ in chloroform from an excitation wavelength of 370 nm before (left) and after (right) complete photobleaching with UVC light. The intensity units were arbitrary, but identical in both cases, indicating the relative magnitudes of the emission intensities of both samples. 31

Figure S 43: Emission spectra of a 1x10^{-4}M solution of K[Eu(BTFA)$_4$] in chloroform from an excitation wavelength of 370 nm before (left) and after (right) complete photobleaching by UVC light. The intensity units are arbitrary, but identical for both cases indicating the relative magnitude se of the emission intensities from both samples. 31

Figure S 44: Emission spectra of a 1x10^{-4}M solution of $P_{6,6,6,14}$[Eu(BTFA)$_4$] in chloroform from an excitation wavelength of 370 nm before (left) and after (right) complete photobleaching with UVC light. The intensity units were arbitrary but identical in both cases, indicating the relative magnitudes of the emission intensities of both samples. 32

Figure S 45: Emission spectra of P_{66614}[Eu(BTFA)$_4$] solutions in acetonitrile (left) and chloroform (right) from an excitation wavelength of 370 nm after partial photobleaching under UVC and UVA light, respectively. The spectra illustrate the progressive photoreduction of Eu(III) to Eu(II) under UV illumination, as evidenced by the increasing relative intensity of the characteristic Eu(II) emission concurrent with the decreasing Eu(III) emission. The left spectrum exhibits less Eu(II) and more Eu(III), whereas the right spectrum shows the opposite trend, emphasizing the inverse relationship between the two emissions during photoreduction. 32

Figure S 46: Luminescence time decay profile of a 1x10^{-4} M solution of Li[Eu(BTFA)$_4$] complex in $CHCl_3$ at maximum excitation and emission wavelengths of λ= 361 nm and λ= 609 nm, respectively. 33

Figure S 47: Luminescence time decay profile of a 1x10^{-4} M solution of Li[Eu(BTFA)$_4$] complex, in CH_2Cl_2, at maximum excitation and emission wavelengths of 360 nm and 609 nm, respectively. 33

Figure S 48: Luminescence time decay profile of a 1x10^{-4} M solution of Li[Eu(BTFA)$_4$] complex in $(CH_3)_2CO$ at maximum excitation and emission wavelengths of λ= 361 nm and λ= 610 nm, respectively. 34

Figure S 49: Luminescence time decay profile of a 1x10^{-4} M solution of Li[Eu(BTFA)$_4$] complex in CH_3CN at maximum excitation and emission wavelengths of λ= 364 nm and λ= 610 nm, respectively. 34

Figure S 50: Luminescence time decay profile of a 1x10^{-4} M solution of Na[Eu(BTFA)$_4$] complex in $CHCl_3$ at maximum excitation and emission wavelengths of λ= 365 nm and λ= 609 nm, respectively. 35

Figure S 51: Luminescence time decay profile of a 1x10^{-4} M solution of Na[Eu(BTFA)$_4$] in CH_2Cl_2 at maximum excitation and emission wavelengths of λ= 362 nm and λ= 609 nm, respectively. 35

Figure S 52: Luminescence time decay profile of a 1x10^{-4} M solution of Na[Eu(BTFA)$_4$] complex in $(CH_3)_2CO$ at maximum excitation and emission wavelengths of λ= 368 nm and λ= 610 nm, respectively. 36

Figure S 53: Luminescence time decay profile of a 1x10^{-4} M solution of Na[Eu(BTFA)$_4$] complex in CH_3CN at maximum excitation and emission wavelengths of λ= 367 nm and λ= 610 nm, respectively. 36

Figure S 54: Luminescence time decay profile of a 1x10^{-4} M solution of K[Eu(BTFA)$_4$] complex in $CHCl_3$ at maximum excitation and emission wavelengths of λ= 366 nm and λ= 609 nm, respectively. 37

Figure S 55: Luminescence time decay profile of a 1x10^{-4} M solution of K[Eu(BTFA)$_4$] complex in CH_2Cl_2 at maximum excitation and emission wavelengths of λ= 365 nm and λ= 609 nm, respectively. 37

Figure S 56: Luminescence time decay profile of a 1x10^{-4} M solution of K[Eu(BTFA)$_4$] complex in $(CH_3)_2CO$ at maximum excitation and emission wavelengths of λ= 367 nm and λ= 610 nm, respectively. 38

Figure S 57: Luminescence time decay profile of a 1x10^{-4} M solution of K[Eu(BTFA)$_4$] complex in CH_3CN at maximum excitation and emission wavelengths of λ= 368 nm and λ= 610 nm, respectively. 38

Figure S 58: Luminescence time decay profile of a 1x10^{-4} M solution of C_5mim[Eu(BTFA)$_4$] complex in $CHCl_3$ at maximum excitation and emission wavelengths of λ= 366 nm and λ= 611 nm, respectively. 39

Figure S 59: Luminescence time decay profile of a 1x10^{-4} M solution of C_5mim[Eu(BTFA)$_4$] complex in CH_2Cl_2 at maximum excitation and emission wavelengths of λ= 366 nm and λ= 611 nm, respectively. 39

Figure S 60: Luminescence time decay profile of a 1x10^{-4} M solution of C_5mim[Eu(BTFA)$_4$] in $(CH_3)_2CO$ at maximum excitation and emission wavelengths of λ= 363 nm and λ= 611 nm, respectively. 40

Figure S 61: Luminescence time decay profile of a 1x10^{-4} M solution of C_5mim[Eu(BTFA)$_4$] in CH_3CN at maximum excitation and emission wavelengths of λ= 366 nm and λ= 611 nm, respectively. 40

Figure S 62: Luminescence time decay profile of a 1x10^{-4} M solution of $P_{6,6,6,14}$]Eu(BTFA)$_4$] complex in $CHCl_3$ at maximum excitation and emission wavelengths of λ= 369 nm and λ= 610 nm, respectively. 41

Figure S 63: Luminescence time decay profile of a 1x10^{-4} M solution of $P_{6,6,6,14}$[Eu(BTFA)$_4$] in CH_2Cl_2 at maximum excitation and emission wavelengths of λ= 368 nm and λ= 610 nm, respectively. 41

Figure S 64: Luminescence time decay profile of a 1x10^{-4} M solution of $P_{6,6,6,14}$[Eu(BTFA)$_4$] complex in $(CH_3)_2CO$ at maximum excitation and emission wavelengths of λ= 368 nm and λ= 610 nm, respectively. 42

Figure S 65: Luminescence time decay profile of a 1x10^{-4} M solution of $P_{6,6,6,14}$[Eu(BTFA)$_4$] in CH_3CN at maximum excitation and emission wavelengths of λ= 367 nm and λ= 610 nm, respectively. 42

Figure S 66: Cyclic voltammograms from -2,8 to 0 V of the 10 mmol·L^{-1} KBTFA in acetonitrile/0.1 mol·L^{-1} $TBABF_4$, v = 100 mV·s^{-1}, using Ag/AgCl, 3.0 mol·L^{-1} KCl as reference electrode. 44

Figure S 67: Cyclic voltammograms from -2,5 to 0 V of the 10 mmol·L^{-1} KBTFA in acetonitrile/0.1 mol·L^{-1} $TBABF_4$, v = 100 mV·s^{-1}, using Ag/AgCl, 3.0 mol·L^{-1} KCl as reference electrode. 44

Figure S 68: Cyclic voltammograms from -2.0 to 0 V of the 10 mmol·L^{-1} KBTFA in acetonitrile/0.1 mol·L^{-1} $TBABF_4$, v = 100 mV·s^{-1}, using Ag/AgCl, 3.0 mol·L^{-1} KCl as reference electrode. 45

Figure S 69: Cyclic voltammograms from -2.8 to 0 V of the 10 mmol·L^{-1} HBTFA in acetonitrile/0.1 mol·L^{-1} $TBABF_4$, v = 100 mV·s^{-1}, using Ag/AgCl, 3.0 mol·L^{-1} KCl as reference electrode. 45

Figure S 70: Cyclic voltammograms from -1.4 to 0 V of the 10 mmol·L^{-1} HBTFA in acetonitrile/0.1 mol·L^{-1} $TBABF_4$, v = 100 mV·s^{-1}, using Ag/AgCl, 3.0 mol·L^{-1} KCl as reference electrode. 46

Figure S 71: Cyclic voltammograms from -2.8 to 0 V of the 10 mmol·L^{-1} $P_{6,6,6,14}Cl$ in acetonitrile/0.1 mol·L^{-1} $TBABF_4$, v = 100 mV·s^{-1}, using Ag/AgCl, 3.0 mol·L^{-1} KCl as reference electrode. 46

Figure S 72: Cyclic voltammograms from -2,8 to 0 V of the 10 mmol·L^{-1} $P_{6,6,6,14}[Eu^{III}(BTFA)_4]$ in acetonitrile/0.1 mol·L^{-1} $TBABF_4$, v = 100 mV·s^{-1}, using Ag/AgCl, 3.0 mol·L^{-1} KCl as reference electrode.... 47

Figure S 73: Cyclic voltammograms from -1.7 to -1.1 V of the 10 mmol·L^{-1} $P_{6,6,6,14}[Eu^{III}(BTFA)_4]$ in acetonitrile/0.1 mol·L^{-1} $TBABF_4$, v = 100 mV·s^{-1}, using Ag/AgCl, 3.0 mol·L^{-1} KCl as reference electrode. The experiment was performed at the following different scan rates: 100mV/s (black line), 200mV/s (red line), 300mV/s (blue line), 700mV/s (purple line). No oxidation peaks were observed after scan reversion indicating irreversible behavior. 47

Figure S 74: Cyclic voltammograms from -2.8 to 0 V of the 10 mmol·L^{-1} $P_{6,6,6,14}[Eu^{II}(BTFA)_4]$ after electrolysis in acetonitrile/0.1 mol·L^{-1} $TBABF_4$, v = 100 mV·s^{-1}, using Ag/AgCl, 3.0 mol·L^{-1} KCl as reference electrode.48

Figure S 75: Cyclic voltammograms from -2.8 to 0 V of the 10 mmol·L^{-1} $P_{6,6,6,14}[Eu^{II}(BTFA)_4]$ after UV irradiation in acetonitrile/0.1 mol·L^{-1} $TBABF_4$, v = 100 mV·s^{-1}, using Ag/AgCl, 3.0 mol·L^{-1} KCl as reference electrode. 48

Figure S 76: Polynomial fit ln(I) = a + bE + CE^2 of the 1x10^{-4} M solution of $Li[Eu(BTFA)_4]$ complex in $CHCl_3$ as a function of the UVA energy. 49

Figure S 77: Polynomial fit ln(I) = a + bE + CE^2 of the 1x10^{-4} M solution of $Li[Eu(BTFA)_4]$ complex in CH_2Cl_2 as a function of UVA energy. 49

Figure S 78: Polynomial fit ln(I) = a + bE + CE^2 of the 1x10^{-4} M solution of $Li[Eu(BTFA)_4]$ complex in $(CH_3)_2CO$ as a function of UVA energy. 50

Figure S 79: Polynomial fit ln(I) = a + bE + CE^2 of the 1x10^{-4} M solution of $Li[Eu(BTFA)_4]$ complex in CH_3CN as a function of UVA energy. 50

Figure S 80: Polynomial fit ln(I) = a + bE + CE^2 of the 1x10^{-4} M solution of $Na[Eu(BTFA)_4]$ complex in $CHCl_3$ as a function of the UVA energy. 51

Figure S 81: Polynomial fit ln(I) = a + bE + CE^2 of the 1x10^{-4} M solution of $Na[Eu(BTFA)_4]$ complex in CH_2Cl_2 as a function of UVA energy. 51

Figure S 82: Polynomial fit ln(I) = a + bE + CE^2 of the 1x10^{-4} M solution of $Na[Eu(BTFA)_4]$ complex in $(CH_3)_2CO$ as a function of UVA energy. 52

Figure S 83: Polynomial fit ln(I) = a + bE + CE^2 of the 1x10^{-4} M solution of $Na[Eu(BTFA)_4]$ complex in CH_3CN as a function of UVA energy. 52

Figure S 84: Polynomial fit ln(I) = a + bE + CE^2 of the 1x10^{-4} M solution of $K[Eu(BTFA)_4]$ complex in $CHCl_3$ as a function of the UVA energy. 53

Figure S 85: Polynomial fit ln(I) = a + bE + CE^2 of the $1x10^{-4}$ M solution of K[Eu(BTFA)$_4$] complex in CH_2Cl_2 as a function of UVA energy. 53

Figure S 86: Polynomial fit ln(I) = a + bE + CE^2 of the $1x10^{-4}$ M solution of K[Eu(BTFA)$_4$] complex in $(CH_3)_2CO$ as a function of UVA energy. 54

Figure S 87: Polynomial fit ln(I) = a + bE + CE^2 of the $1x10^{-4}$ M solution of K[Eu(BTFA)$_4$] complex in CH_3CN as a function of UVA energy. 54

Figure S 88: Polynomial fit ln(I) = a + bE + CE^2 of the $1x10^{-4}$ M solution of C_5mim[Eu(BTFA)$_4$] in $CHCl_3$ as a function of UVA energy. 55

Figure S 89: Polynomial fit ln(I) = a + bE + CE^2 of the $1x10^{-4}$ M solution of C_5mim[Eu(BTFA)$_4$] complex in CH_2Cl_2 as a function of UVA energy. 55

Figure S 90: Polynomial fit ln(I) = a + bE + CE^2 of the $1x10^{-4}$ M solution of C_5mim[Eu(BTFA)$_4$] complex in $(CH_3)_2CO$ as a function of UVA energy. 56

Figure S 91: Polynomial fit ln(I) = a + bE + CE^2 of the $1x10^{-4}$ M solution of C_5mim[Eu(BTFA)$_4$] complex in CH_3CN as a function of UVA energy. 56

Figure S 92: Polynomial fit ln(I) = a + bE + CE^2 of the $1x10^{-4}$ M solution of $P_{6,6,6,14}$[Eu(BTFA)$_4$] complex in CH_2Cl_2 as a function of UVA energy.. 57

Figure S 93: Polynomial fit ln(I) = a + bE + CE^2 of the $1x10^{-4}$ M solution of $P_{6,6,6,14}$[Eu(BTFA)$_4$] complex in $(CH_3)_2CO$ as a function of UVA energy. 57

Figure S 94: Polynomial fit ln(I) = a + bE + CE^2 of the $1x10^{-4}$ M solution of $P_{6,6,6,14}$[Eu(BTFA)$_4$] complex in CH_3CN as a function of UVA energy. 58

Figure S 95: ^{1}H NMR spectrum of K[Eu(BTFA)$_4$] complex acquired using a 400 MHz spectrometer in acetone-d. 59

Figure S 96: ^{1}H NMR spectrum of the Na[Eu(BTFA)$_4$] complex. Acquired on a 400 MHz spectrometer in Acetone-d. 59

Figure S 97: ^{1}H NMR spectrum of C_5mim[Eu(BTFA)$_4$] complex. Acquired on a 400 MHz spectrometer in chloroform-d. 60

Figure S 98: ^{1}H NMR spectrum of the $P_{6,6,6,14}$[Eu(BTFA)$_4$] complex. Acquired on a 400 MHz spectrometer in chloroform-d. 60

Figure S 99: Infrared spectrum of K[Eu(BTFA)$_4$] complex. Acquired in KBr disk. 61

Figure S 100: Infrared spectrum of Na[Eu(BTFA)$_4$] complex. Acquired in KBr disk. 61

Figure S 101: Infrared spectrum of C_5mim[Eu(BTFA)$_4$] complex. Acquired in KBr disk. 62

Figure S 102: Infrared spectrum of $P_{6,6,6,14}$[Eu(BTFA)$_4$] complex. Acquired in KBr disk. 62

1. Luminescence

1.1 Excitation Spectra

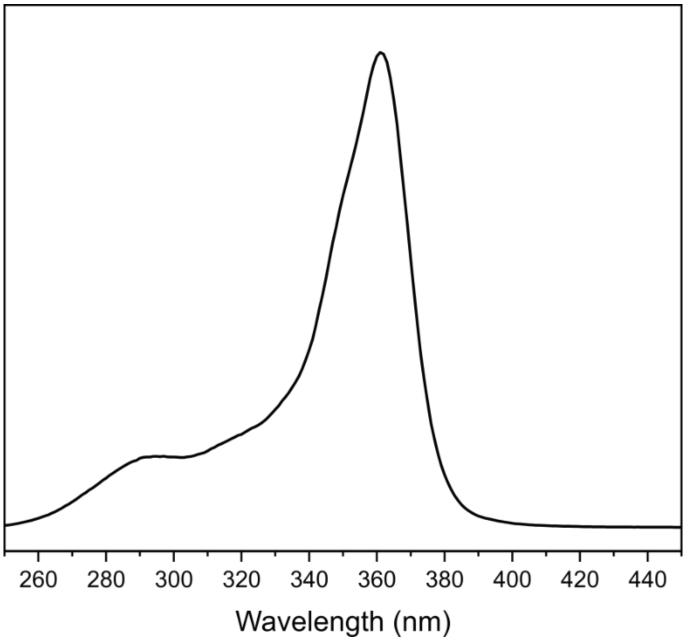

Figure S 1: Excitation spectrum of a 1x10^{-4} M solution of a 1x10^{-4} M solution of Li[Eu(BTFA)$_4$] complex in $CHCl_3$ with a maximum excitation wavelength of λ= 361 nm.

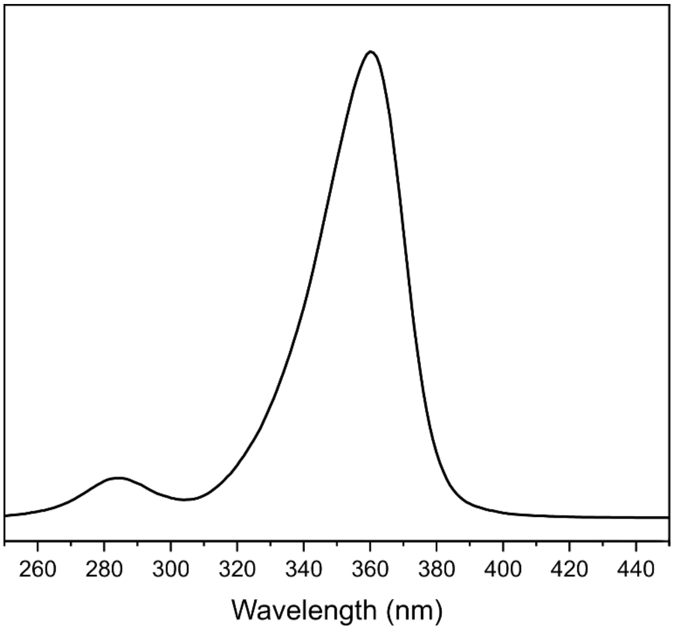

Figure S 2: Excitation spectrum of a 1x10^{-4} M solution of a 1x10^{-4}M solution of Li[Eu(BTFA)$_4$] complex in CH_2Cl_2 with a maximum excitation wavelength of λ= 360 nm.

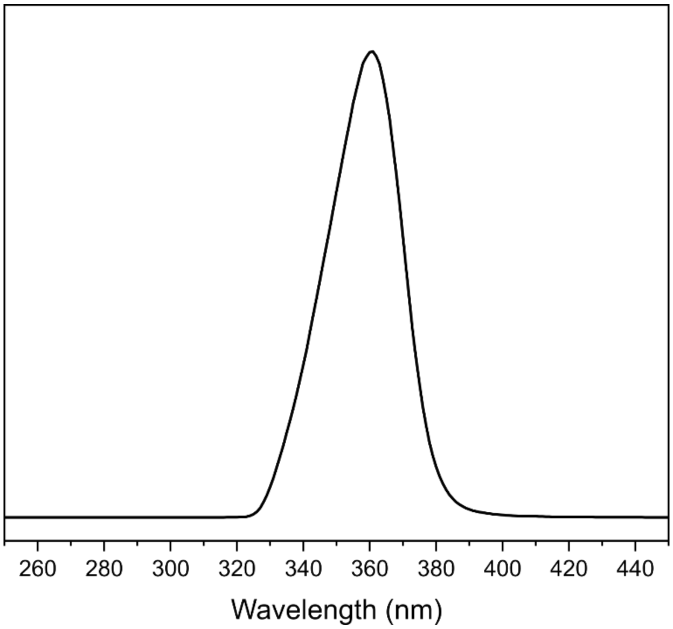

Figure S 3: Excitation spectrum of a $1x10^{-4}$ M solution of Li[Eu(BTFA)$_4$] complex in $(CH_3)_2CO$ with a maximum excitation wavelength of λ= 361 nm.

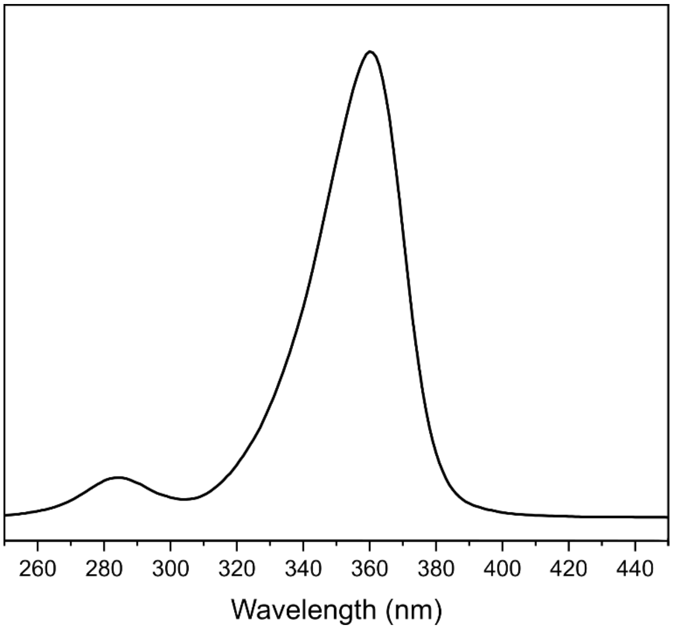

Figure S 4: Excitation spectrum of a $1x10^{-4}$ M solution of a $1x10^{-4}$M solution of Li[Eu(BTFA)$_4$] complex in CH_3CN with a maximum excitation wavelength of λ= 364 nm.

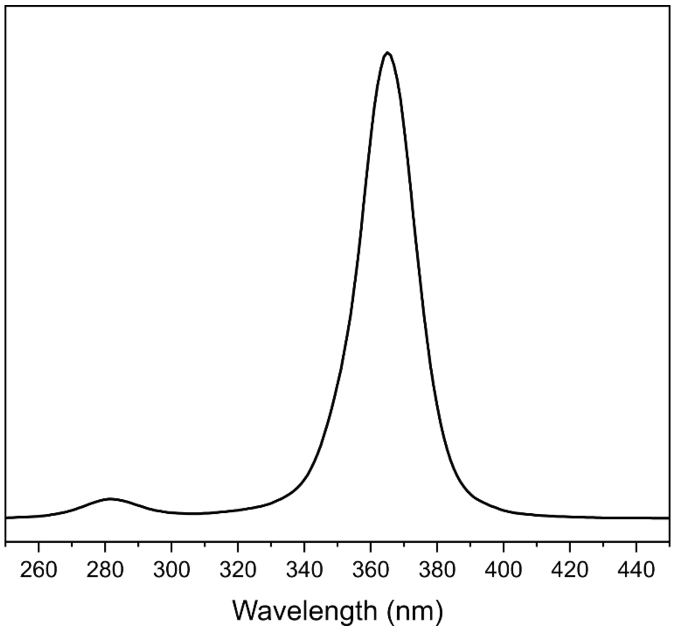

Figure S 5: Excitation spectrum of a 1x10^{-4} M solution of Na[Eu(BTFA)$_4$] complex in $CHCl_3$ with a maximum excitation wavelength of λ= 365 nm.

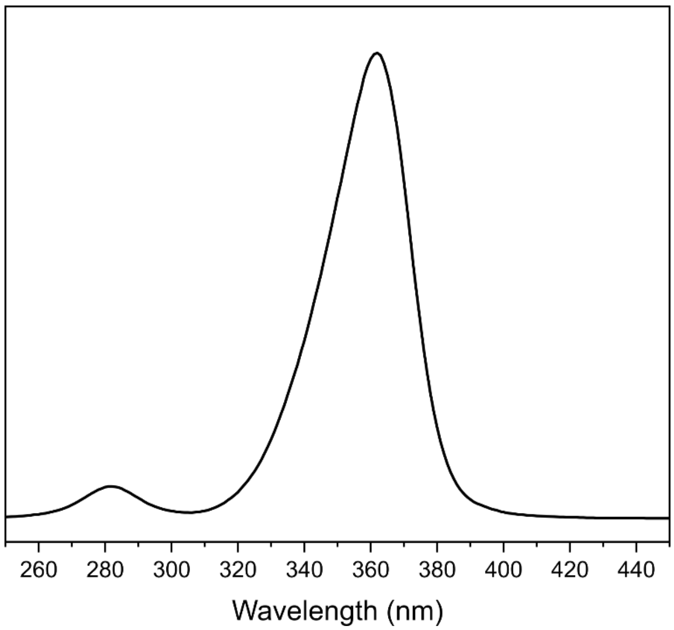

Figure S 6: Excitation spectrum of a 1x10^{-4} M solution of Na[Eu(BTFA)$_4$] complex in CH_2Cl_2 with a maximum excitation wavelength of λ= 362 nm.

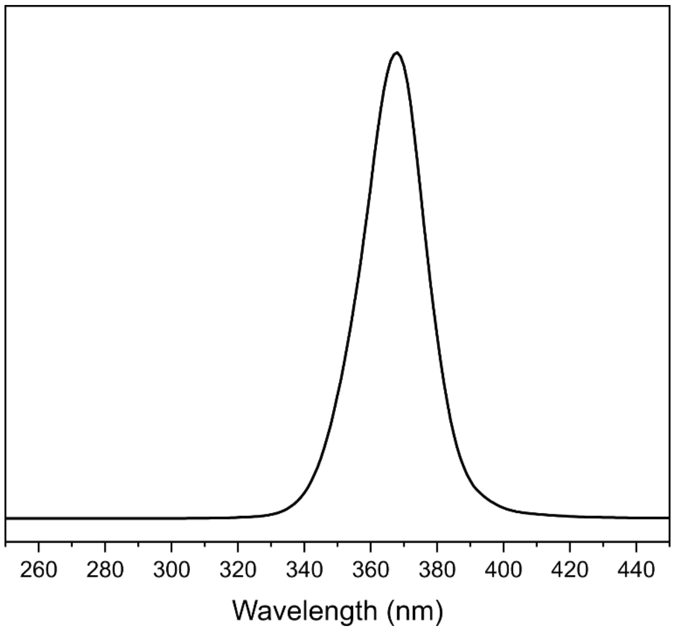

Figure S 7: Excitation spectrum of a $1x10^{-4}$ M solution of Na[Eu(BTFA)$_4$] complex in $(CH_3)_2CO$ with a maximum excitation wavelength of λ= 368 nm.

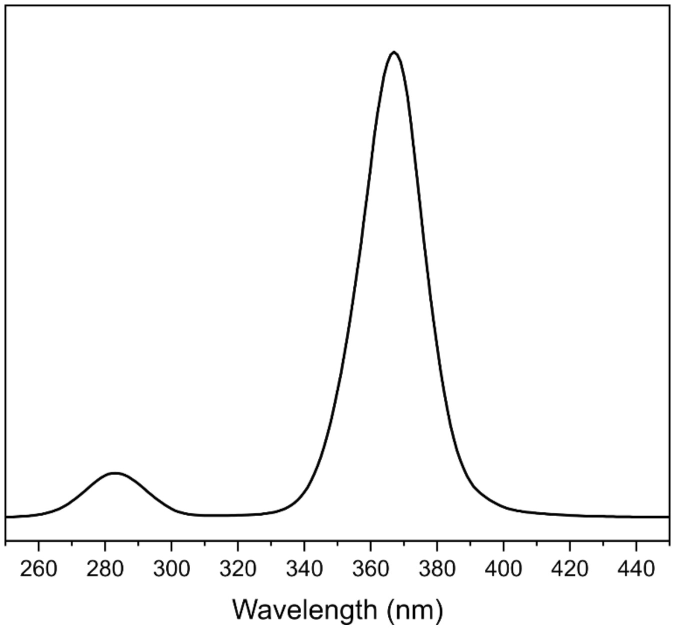

Figure S 8: Excitation spectrum of a $1x10^{-4}$ M solution of Na[Eu(BTFA)$_4$] complex in CH_3CN with a maximum excitation wavelength of λ= 367 nm.

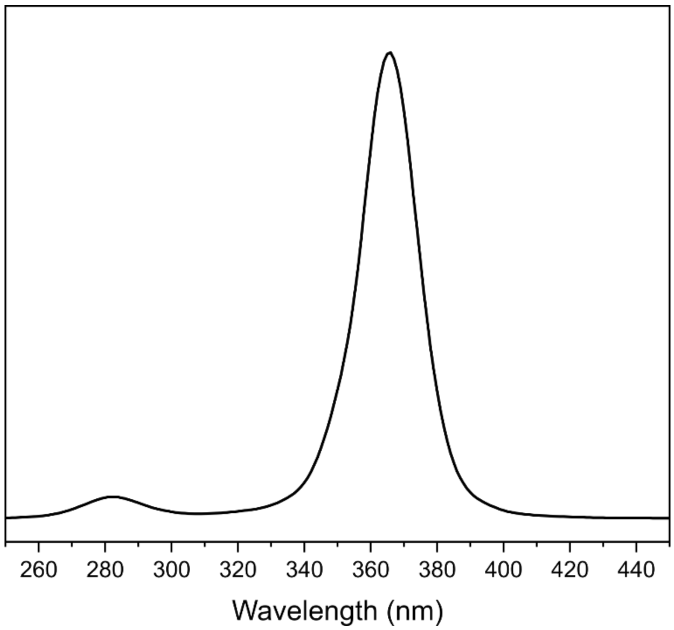

Figure S 9: Excitation spectrum of a 1x10^{-4} M solution of K[Eu(BTFA)$_4$] complex in $CHCl_3$ with a maximum excitation wavelength of λ= 366 nm.

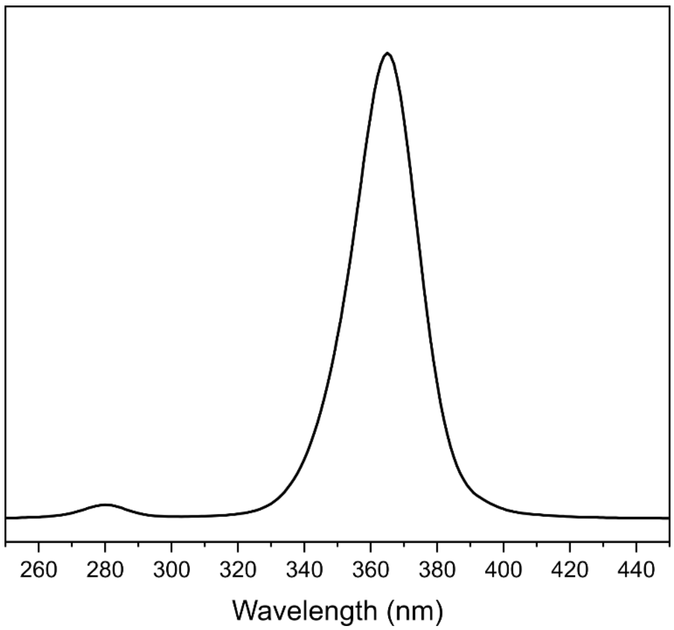

Figure S 10: Excitation spectrum of a 1x10^{-4} M solution of K[Eu(BTFA)$_4$] complex in CH_2Cl_2 with a maximum excitation wavelength of λ= 365 nm.

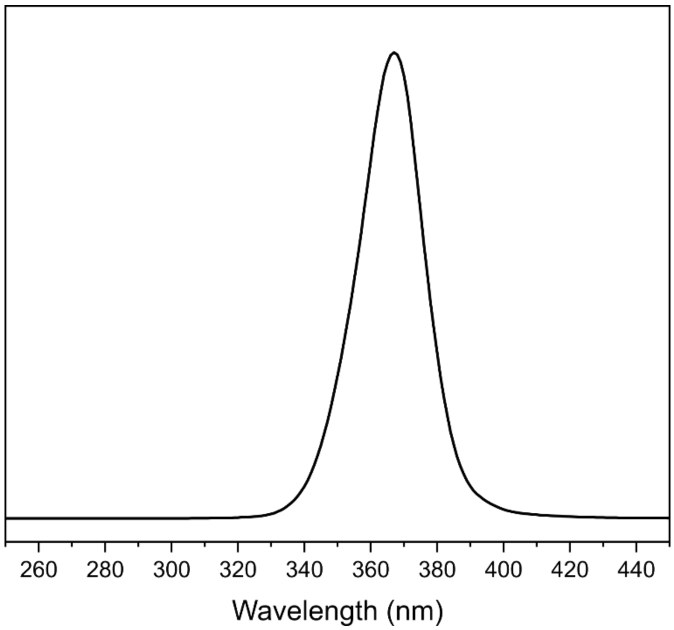

Figure S 11: Excitation spectrum of a $1x10^{-4}$ M solution of K[Eu(BTFA)$_4$] complex in $(CH_3)_2CO$ with a maximum excitation wavelength of λ= 367 nm.

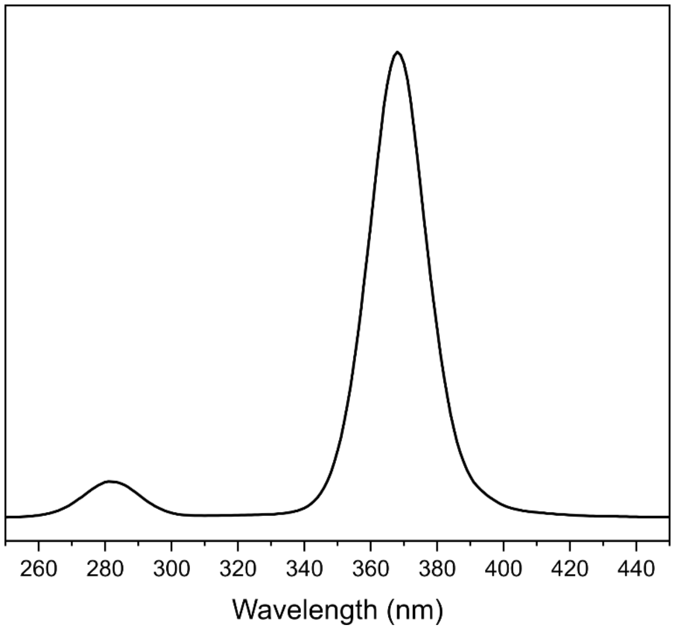

Figure S 12: Excitation spectrum of a $1x10^{-4}$ M solution of K[Eu(BTFA)$_4$] complex in CH_3CN with a maximum excitation wavelength of λ= 368 nm.

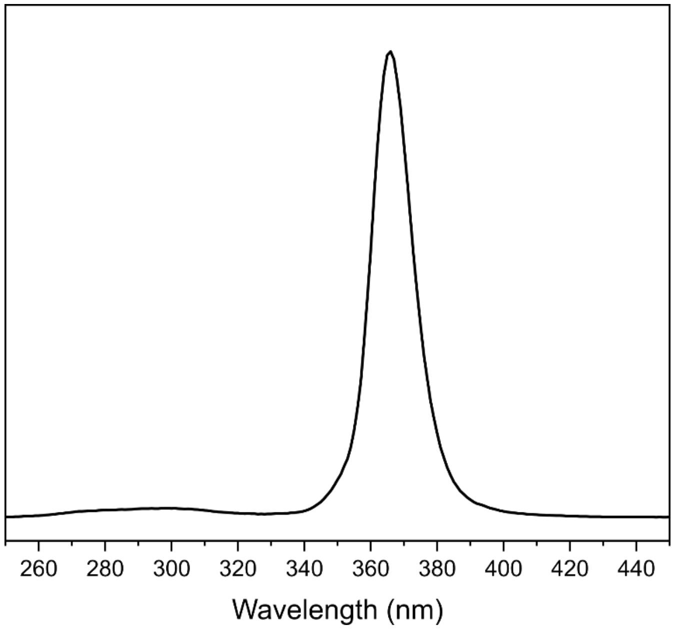

Figure S 13: Excitation spectrum of a $1x10^{-4}$ M solution of C_5mim[Eu(BTFA)$_4$] complex in $CHCl_3$ with a maximum excitation wavelength of λ= 366 nm.

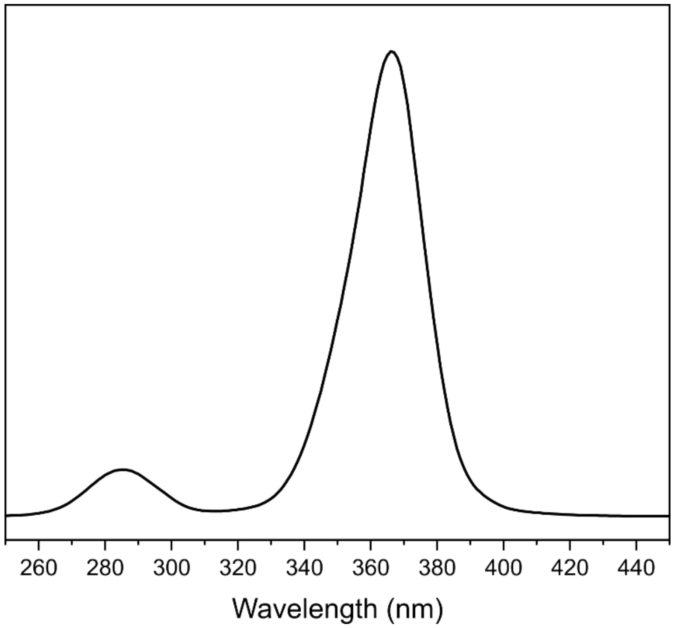

Figure S 14: Excitation spectrum of a $1x10^{-4}$ M solution of C_5mim[Eu(BTFA)$_4$] complex in CH_2Cl_2 with a maximum excitation wavelength of λ= 366 nm.

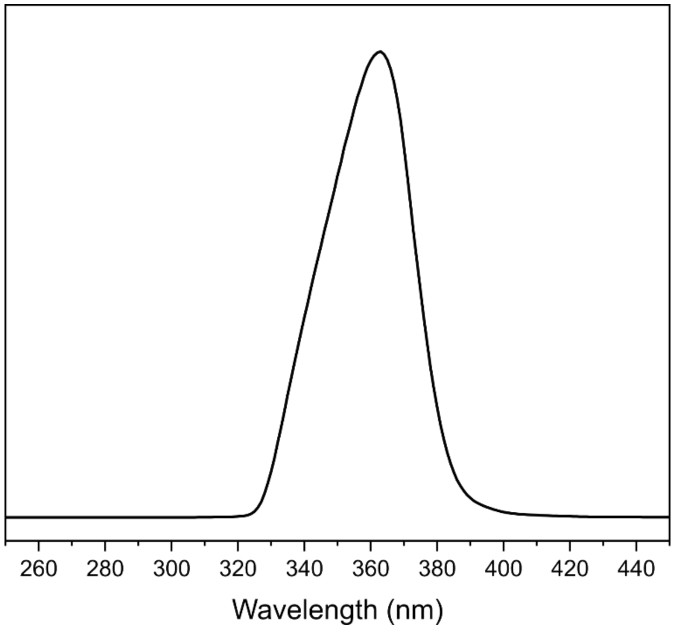

Figure S 15: Excitation spectrum of a $1x10^{-4}$ M solution of C_5mim[Eu(BTFA)$_4$] complex in $(CH_3)_2CO$ with a maximum excitation wavelength of λ= 363 nm.

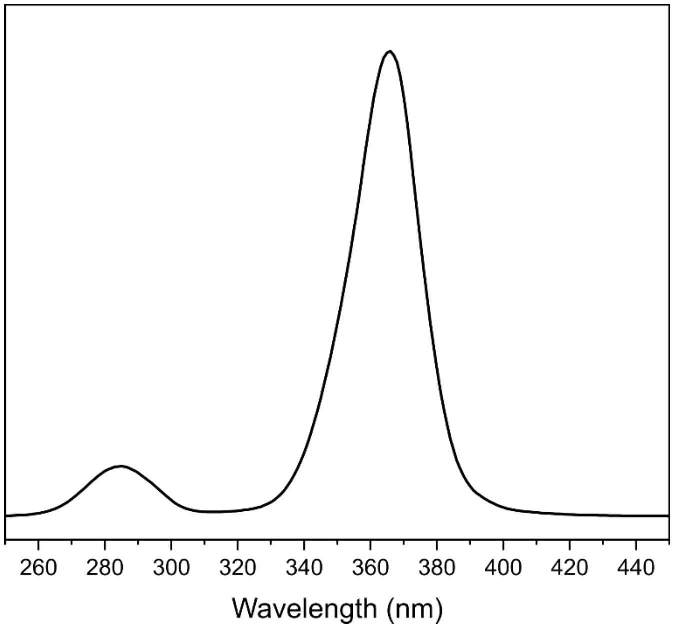

Figure S 16: Excitation spectrum of a $1x10^{-4}$ M solution of C_5mim[Eu(BTFA)$_4$] complex in CH_3CN with a maximum excitation wavelength of λ= 366 nm.

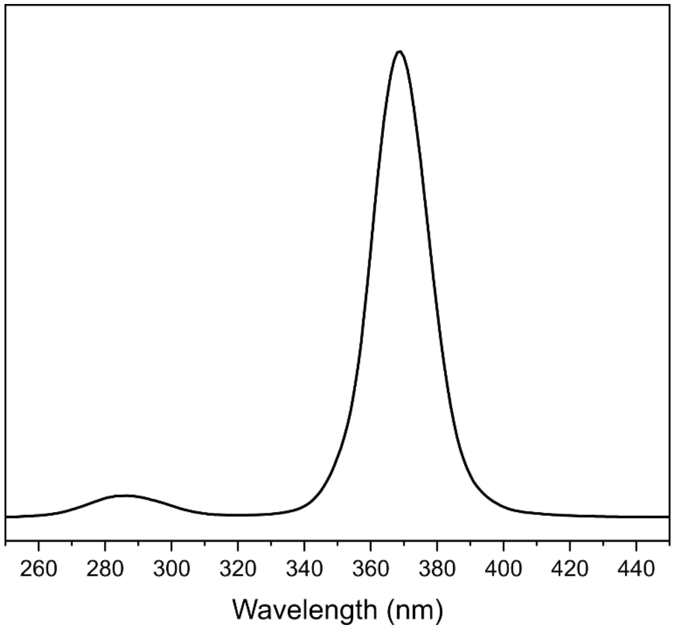

Figure S 17: Excitation spectrum of a 1x10^{-4} M solution of $P_{6,6,6,14}$[Eu(BTFA)$_4$] complex in $CHCl_3$ with a maximum excitation wavelength of λ= 369 nm.

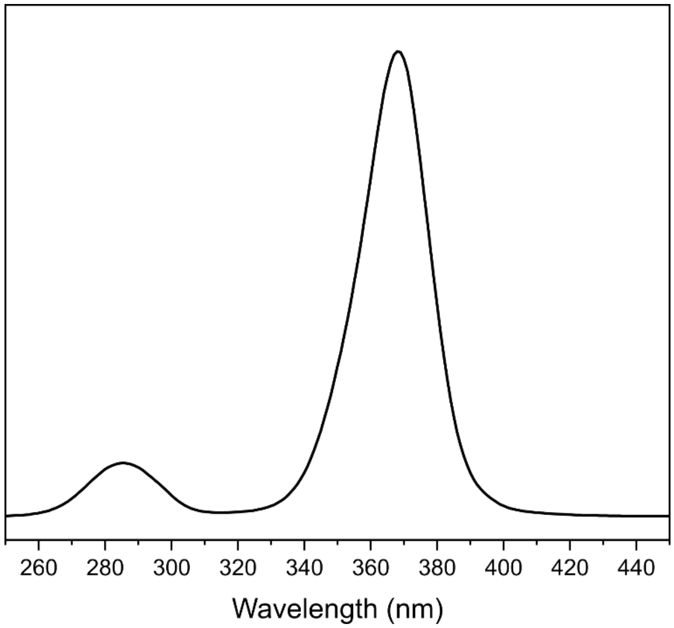

Figure S 18: Excitation spectrum of a 1x10^{-4} M solution of $P_{6,6,6,14}$[Eu(BTFA)$_4$] complex in CH_2Cl_2 with a maximum excitation wavelength of λ= 368 nm.

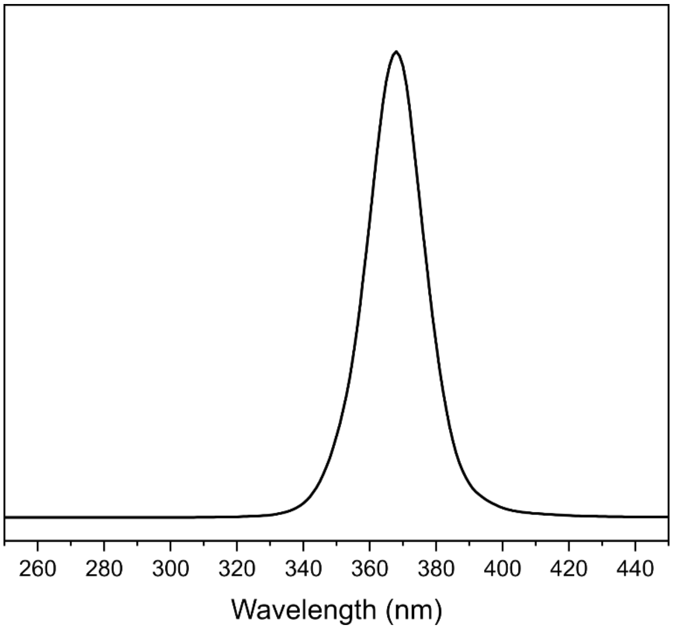

Figure S 19: Excitation spectrum of a 1x10^{-4} M solution of $P_{6,6,6,14}[Eu(BTFA)_4]$ complex in $(CH_3)_2CO$ with a maximum excitation wavelength of λ= 368 nm.

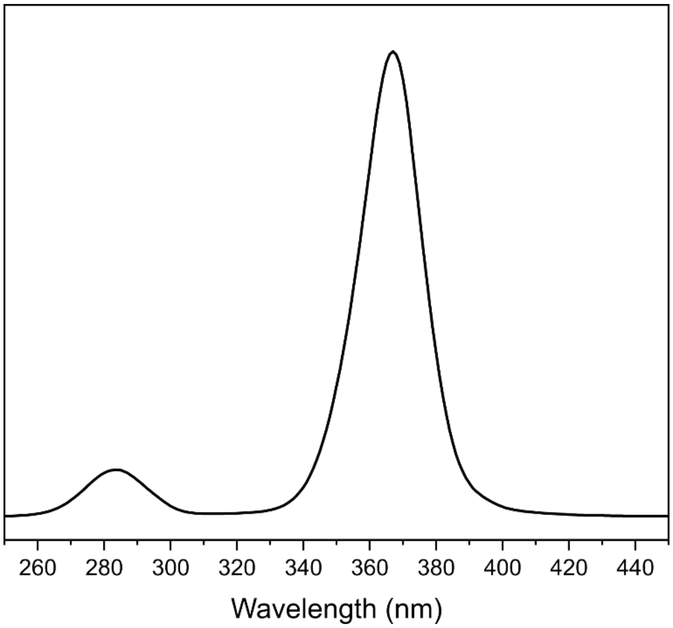

Figure S 20: Excitation spectrum of a 1x10^{-4} M solution of $P_{6,6,6,14}[Eu(BTFA)_4]$ complex in CH_3CN with a maximum excitation wavelength of λ= 367 nm

1.2 Emission Spectra

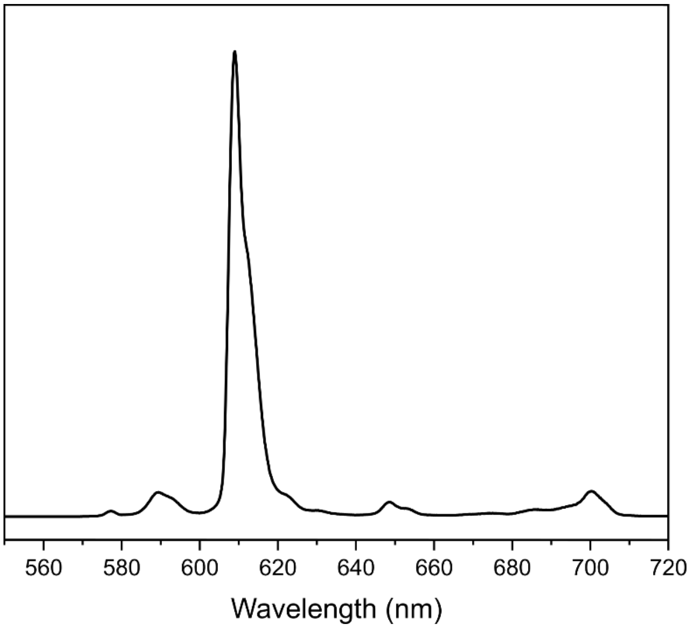

Figure S 21: Emission spectrum of a $1x10^{-4}$ M solution of Li[Eu(BTFA)$_4$] complex in $CHCl_3$ with a maximum emission wavelength of λ= 609 nm.

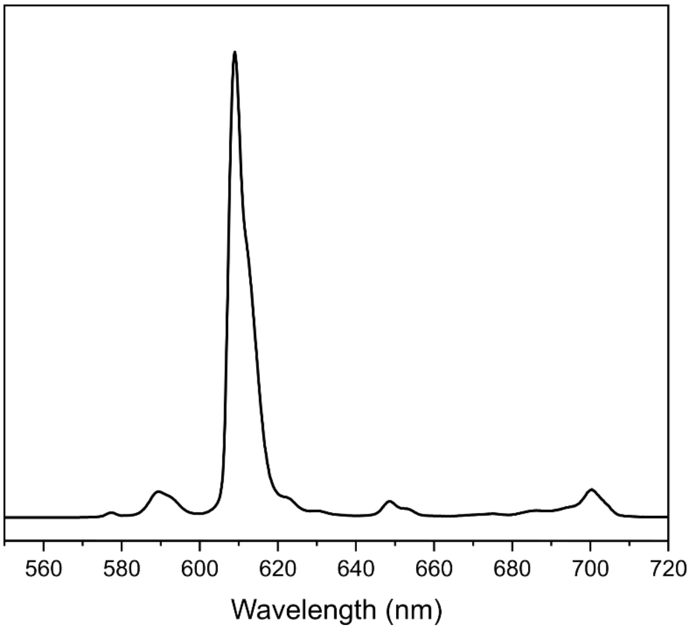

Figure S 22: Emission spectrum of a $1x10^{-4}$ M solution of Li[Eu(BTFA)$_4$] complex in CH_2Cl_2 with a maximum emission wavelength of λ= 609 nm

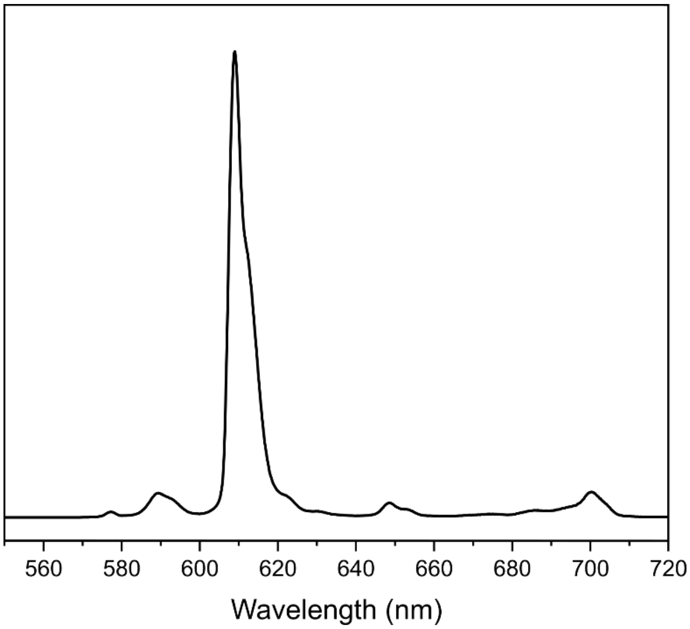

Figure S 23: Emission spectrum of a $1x10^{-4}$ M solution of Li[Eu(BTFA)$_4$] complex in $(CH_3)_2CO$ with a maximum emission wavelength of λ= 610 nm

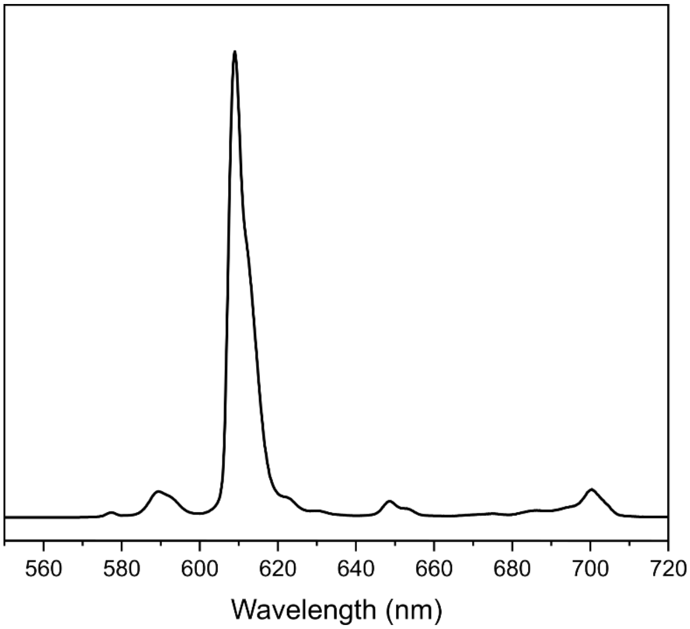

Figure S 24: Emission spectrum of a $1x10^{-4}$ M solution of Li[Eu(BTFA)$_4$] complex in CH_3CN with a maximum emission wavelength of λ= 610 nm.

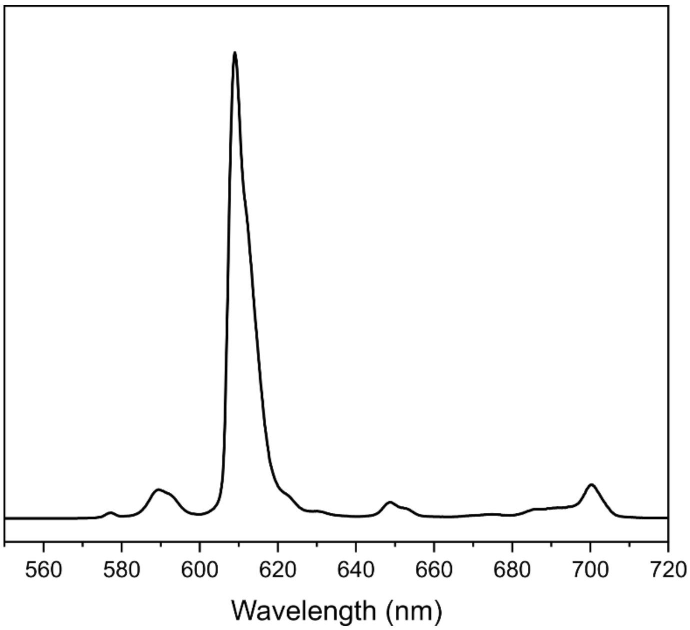

Figure S 25: Emission spectrum of a $1x10^{-4}$ M solution of Na[Eu(BTFA)$_4$] complex in $CHCl_3$ with a maximum emission wavelength of λ= 609 nm.

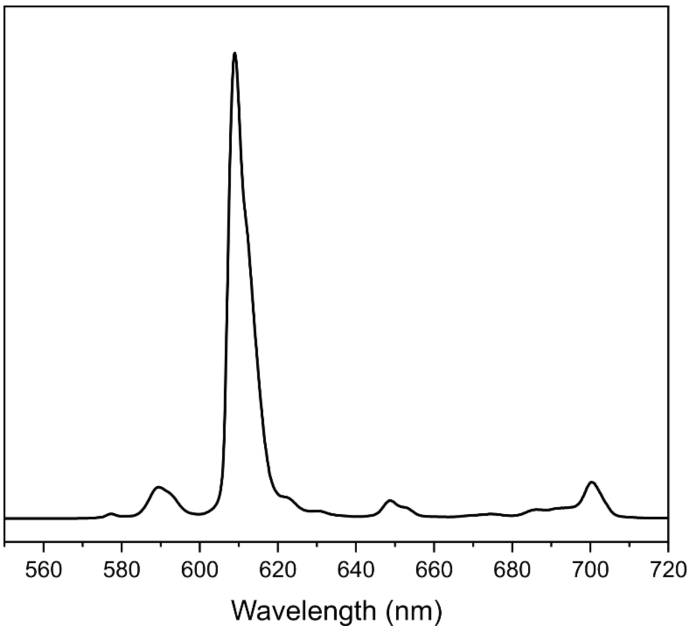

Figure S 26: Emission spectrum of a $1x10^{-4}$ M solution of Na[Eu(BTFA)$_4$] complex in CH_2Cl_2 with a maximum emission wavelength of λ= 609 nm.

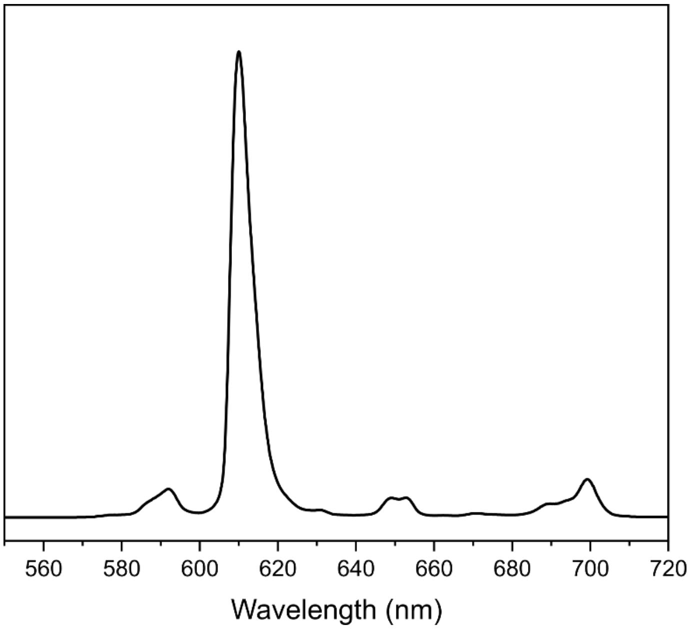

Figure S 27: Emission spectrum of a $1x10^{-4}$ M solution of $Na[Eu(BTFA)_4]$ complex in $(CH_3)_2CO$ with a maximum emission wavelength of λ= 610 nm.

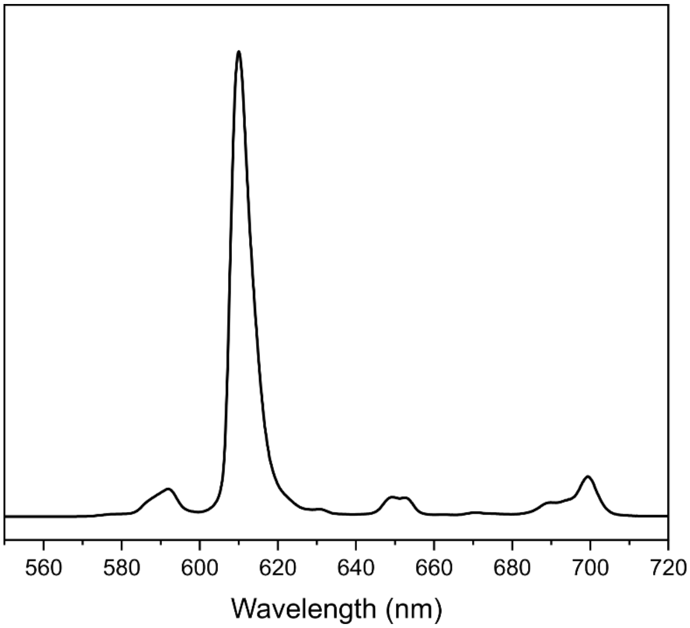

Figure S 28: Emission spectrum of a $1x10^{-4}$ M solution of $Na[Eu(BTFA)_4]$ complex in CH_3CN with a maximum emission wavelength of λ= 610 nm.

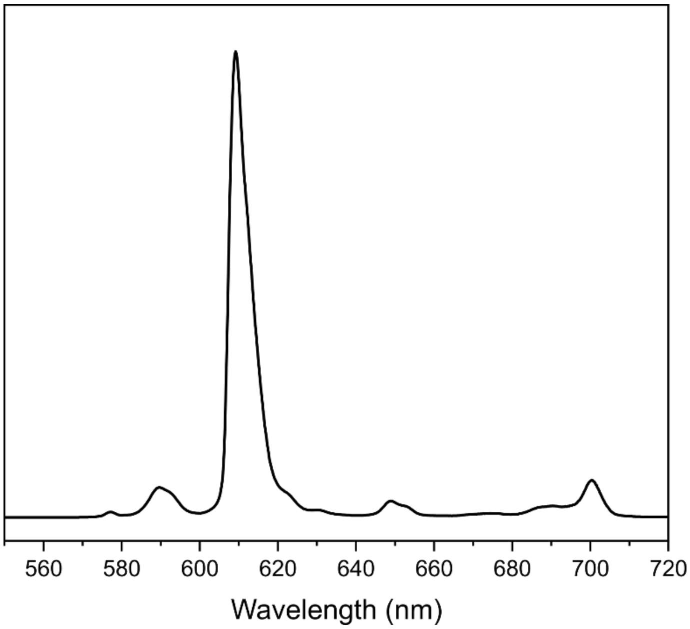

Figure S 29: Emission spectrum of a 1x10^{-4} M solution of K[Eu(BTFA)$_4$] complex in $CHCl_3$ with a maximum emission wavelength of λ= 609 nm.

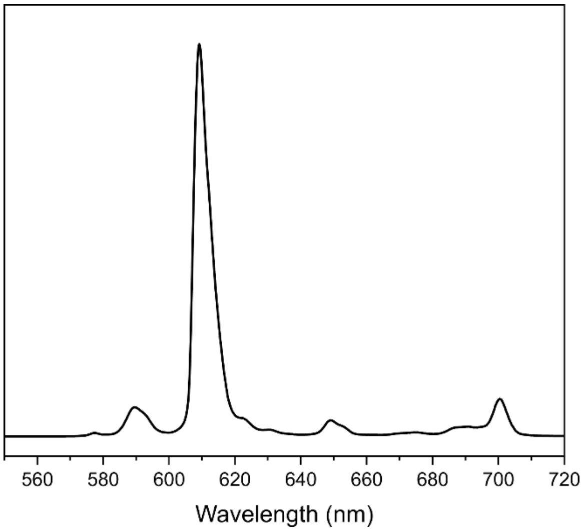

Figure S 30: Emission spectrum of a 1x10^{-4} M solution of K[Eu(BTFA)$_4$] complex in CH_2Cl_2 with a maximum emission wavelength of λ= 609 nm.

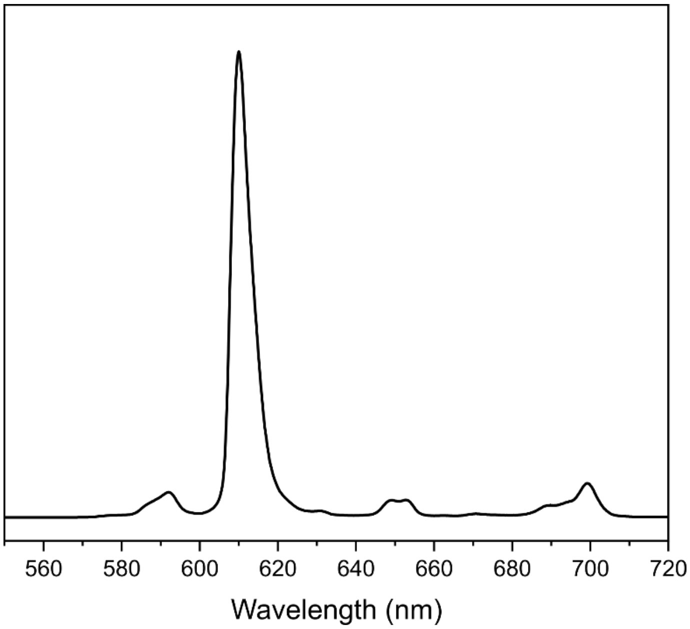

Figure S 31: Emission spectrum of a 1x10^{-4} M solution of K[Eu(BTFA)$_4$] complex in $(CH_3)_2CO$ with a maximum emission wavelength of λ= 610 nm.

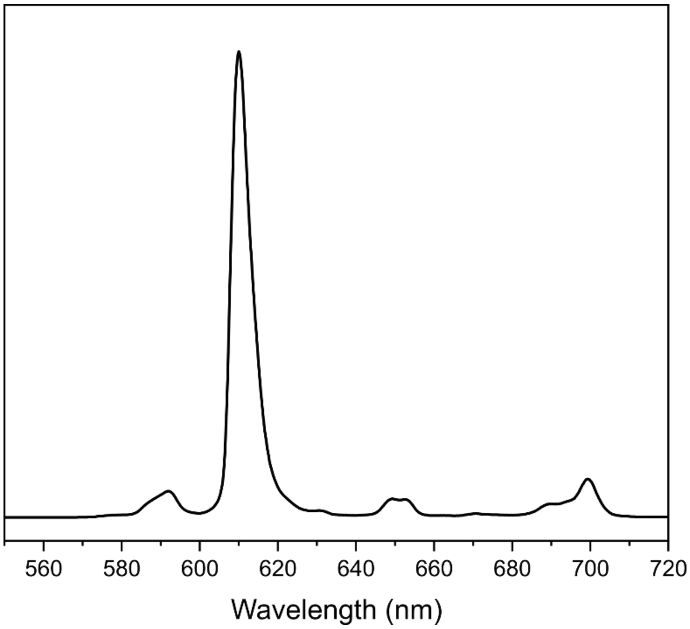

Figure S 32: Emission spectrum of a 1x10^{-4} M solution of K[Eu(BTFA)$_4$] complex in CH_3CN with a maximum emission wavelength of λ= 610 nm

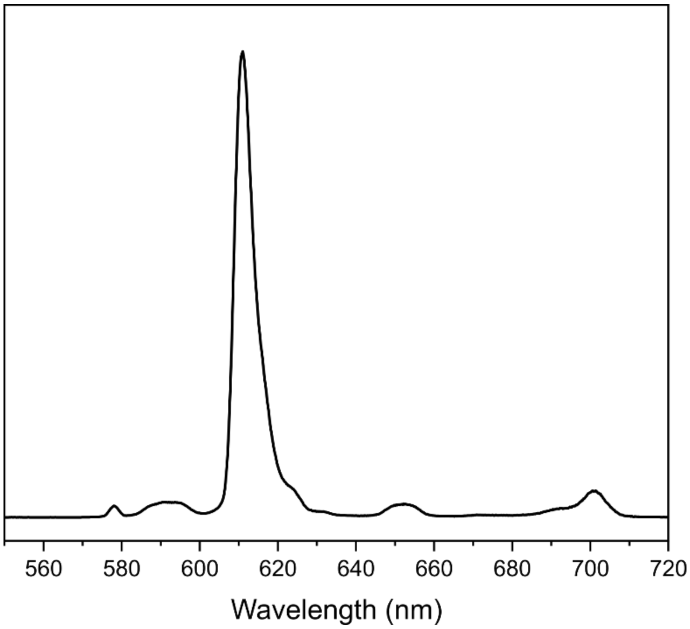

Figure S 33: Emission spectrum of a $1x10^{-4}$ M solution of C_5mim[Eu(BTFA)$_4$] complex in $CHCl_3$ with a maximum emission wavelength of λ= 611 nm.

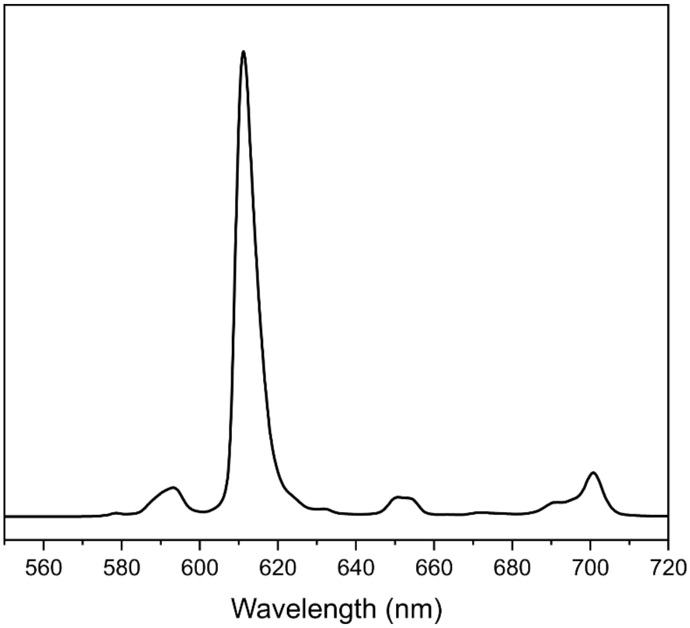

Figure S 34: Emission spectrum of a $1x10^{-4}$M solution of C_5mim[Eu(BTFA)$_4$] complex in CH_2Cl_2 with a maximum emission wavelength of λ= 611 nm.

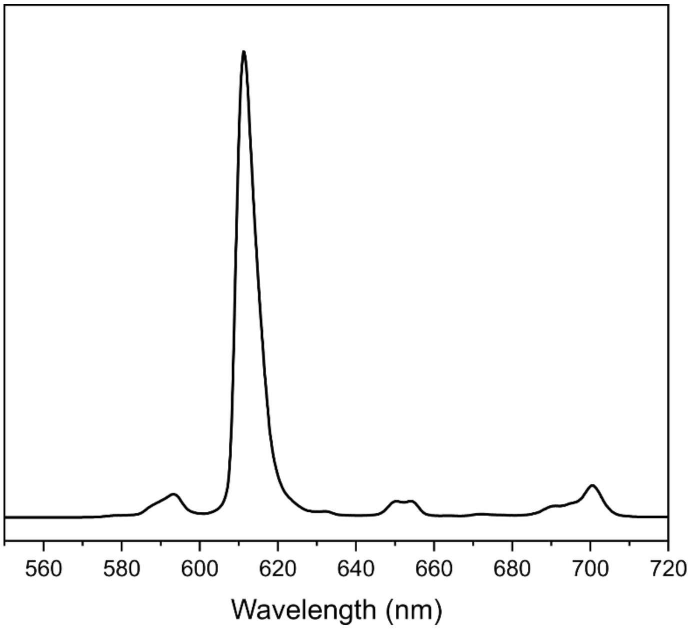

Figure S 35: Emission spectrum of a 1x10^{-4} M solution of C_5mim[Eu(BTFA)$_4$] complex in $(CH_3)_2CO$ with a maximum emission wavelength of λ= 611 nm.

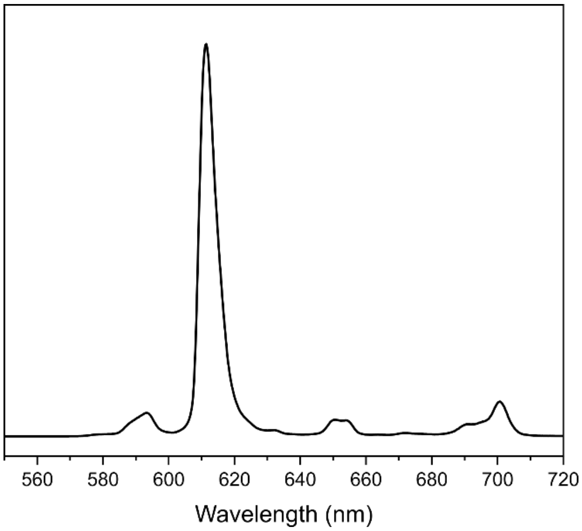

Figure S 36: Emission spectrum of a 1x10^{-4} M solution of C_5mim[Eu(BTFA)$_4$] complex in CH_3CN with a maximum emission wavelength of λ= 611 nm.

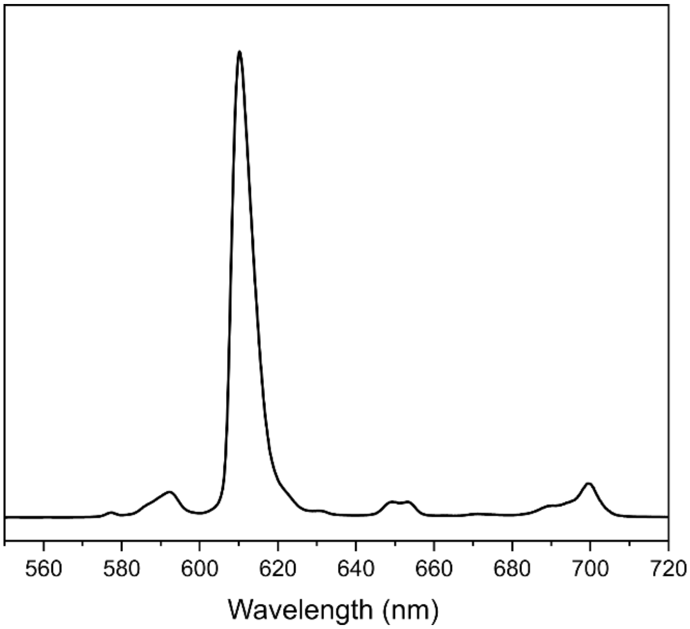

Figure S 37: Emission spectrum of a 1x10^{-4} M solution of $P_{6,6,6,14}[Eu(BTFA)_4]$ complex in $CHCl_3$ with a maximum emission wavelength of λ= 610 nm.

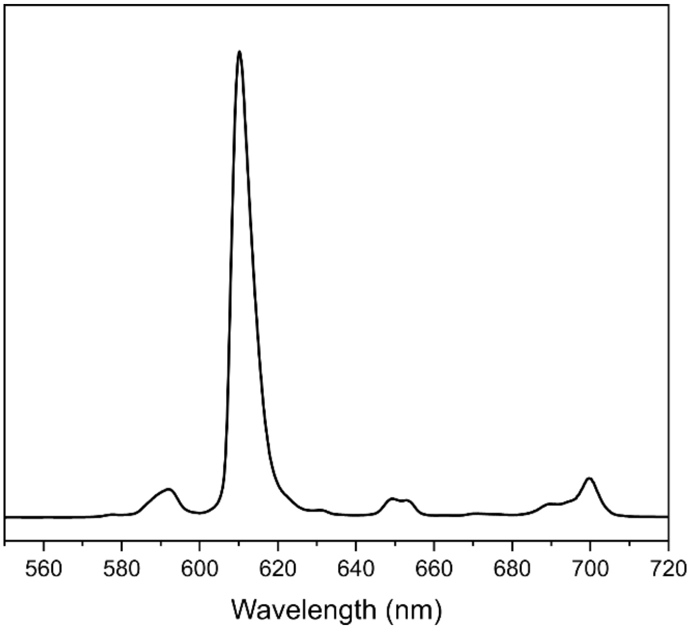

Figure S 38: Emission spectrum of a 1x10^{-4} M solution of $P_{6,6,6,14}[Eu(BTFA)_4]$ complex in CH_2Cl_2 with a maximum emission wavelength of λ= 610 nm.

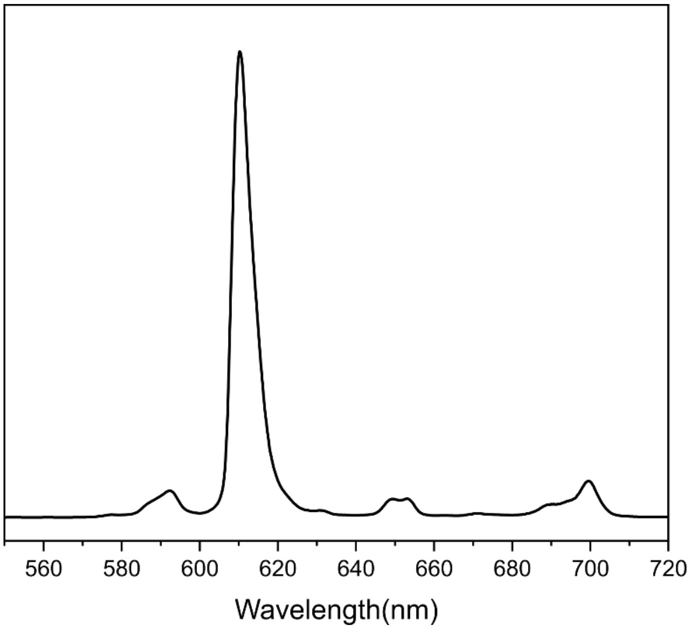

Figure S 39: Emission spectrum of a 1x10^{-4} M solution of $P_{6,6,6,14}$[Eu(BTFA)$_4$] complex in $(CH_3)_2CO$ with a maximum emission wavelength of λ= 610 nm.

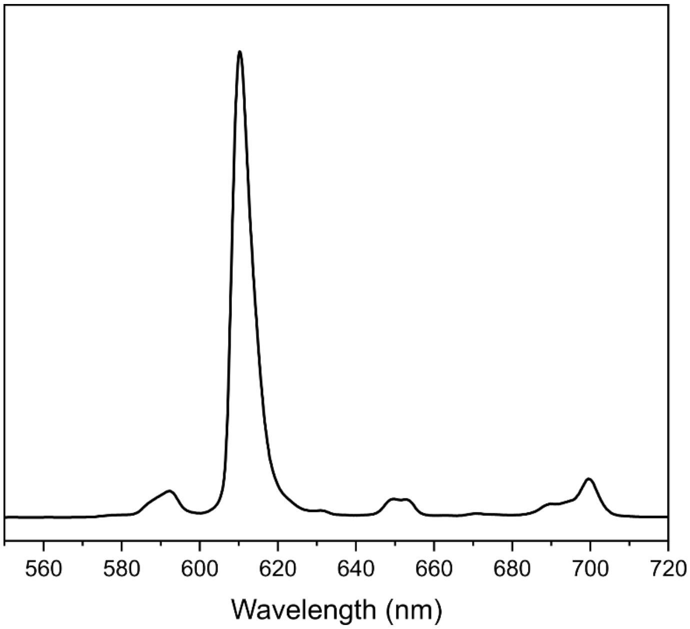

Figure S 40: Emission spectrum of a 1x10^{-4} M solution of $P_{6,6,6,14}$[Eu(BTFA)$_4$] complex in CH_3CN with a maximum emission wavelength of λ= 610 nm.

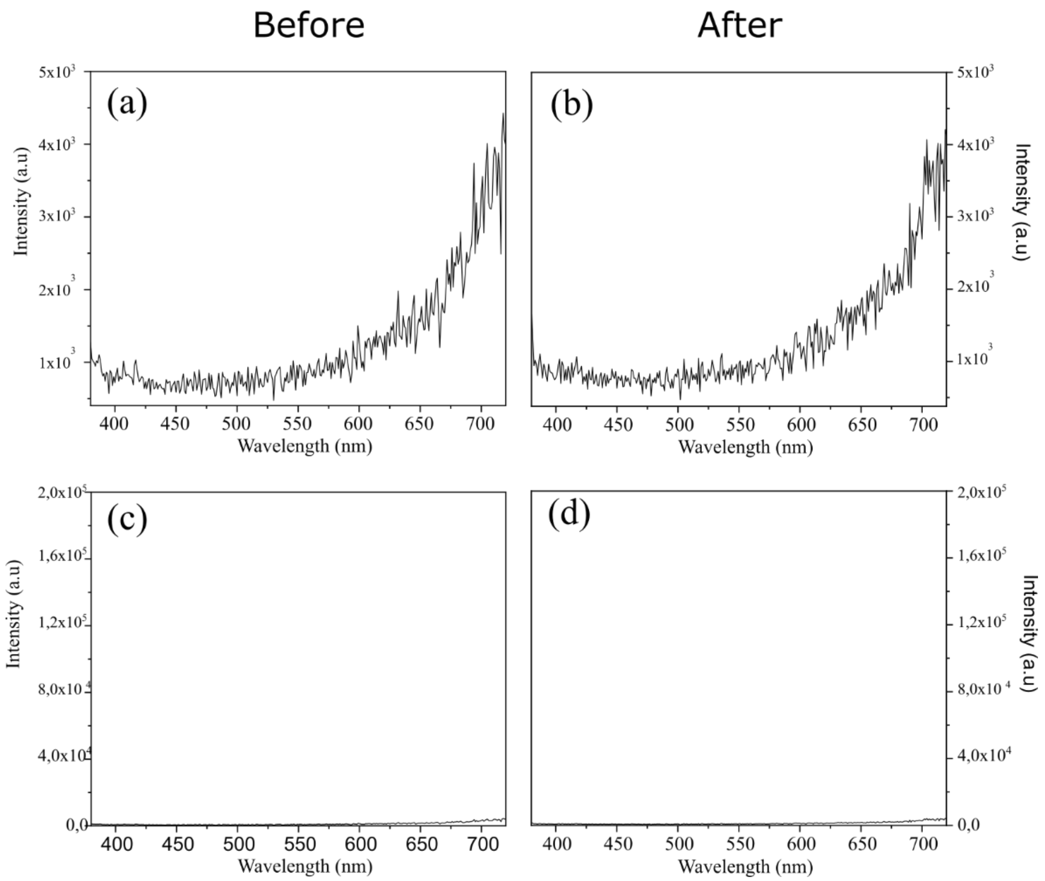

Figure S 41: Emission spectra of a 1x10^{-4} M solution of $P_{6,6,6,14}$[Gd(BTFA)$_4$] complex in chloroform, recorded before [(a) and (c)], and after UVA irradiation [(b) and (d)], from an excitation wavelength of 370 nm. The spectra show no signals in the Eu(II) emission region (400–500 nm). The intensity units are arbitrary but consistent across all four graphs and identical to those in the subsequent experiments, which depict similar conditions for $P_{6,6,6,14}$[Eu(BTFA)$_4$] for comparison, with the scales in panels (c) and (d) also matching those in the subsequent experiments to emphasize the absence of any spectral changes in the range of 400–700 nm after UVA exposure.

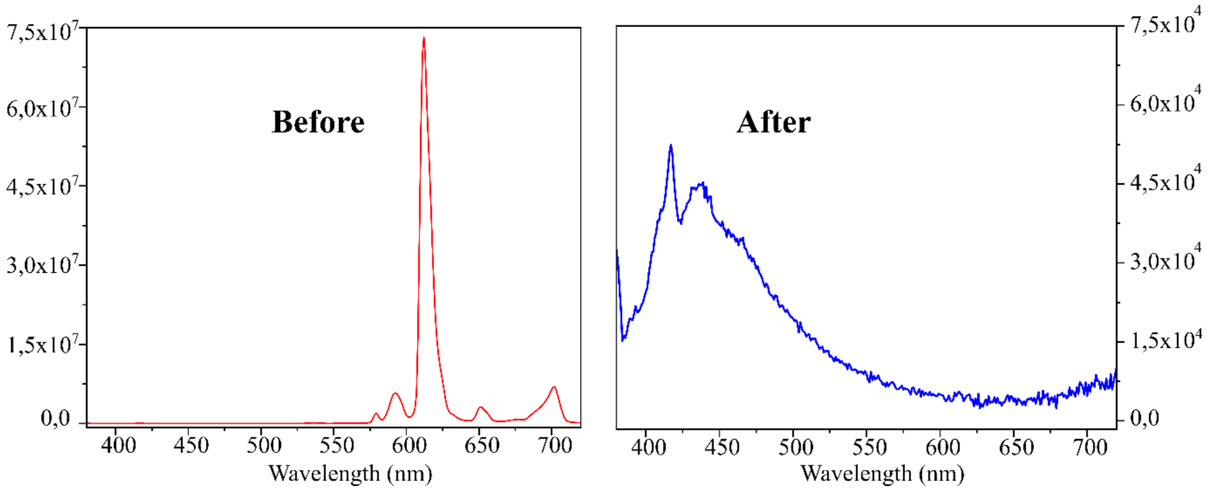

Figure S 42: Emission spectra of a 1x10^{-4}M solution of Li[Eu(BTFA)$_4$] in chloroform from an excitation wavelength of 370 nm before (left) and after (right) complete photobleaching with UVC light. The intensity units were arbitrary, but identical in both cases, indicating the relative magnitudes of the emission intensities of both samples.

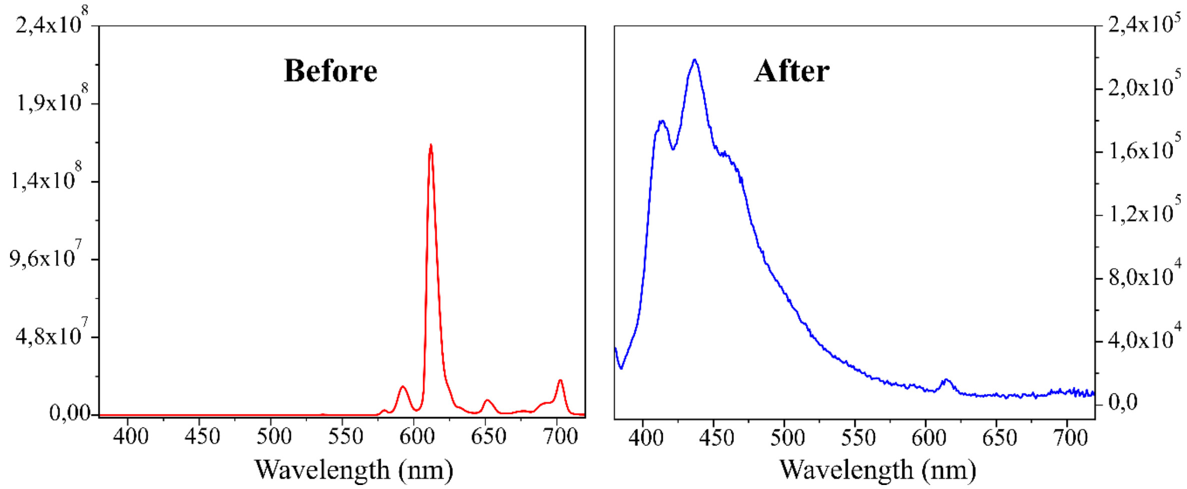

Figure S 43: Emission spectra of a 1x10^{-4}M solution of K[Eu(BTFA)$_4$] in chloroform from an excitation wavelength of 370 nm before (left) and after (right) complete photobleaching by UVC light. The intensity units are arbitrary, but identical for both cases indicating the relative magnitude se of the emission intensities from both samples.

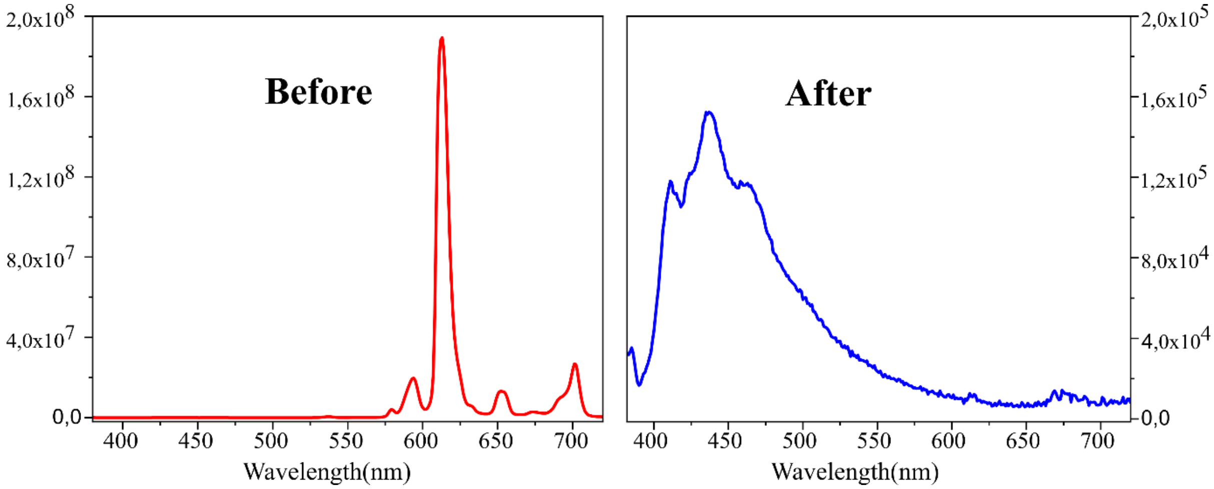

Figure S 44: Emission spectra of a $1x10^{-4}$M solution of $P_{6,6,6,14}$[Eu(BTFA)$_4$] in chloroform from an excitation wavelength of 370 nm before (left) and after (right) complete photobleaching with UVC light. The intensity units were arbitrary but identical in both cases, indicating the relative magnitudes of the emission intensities of both samples.

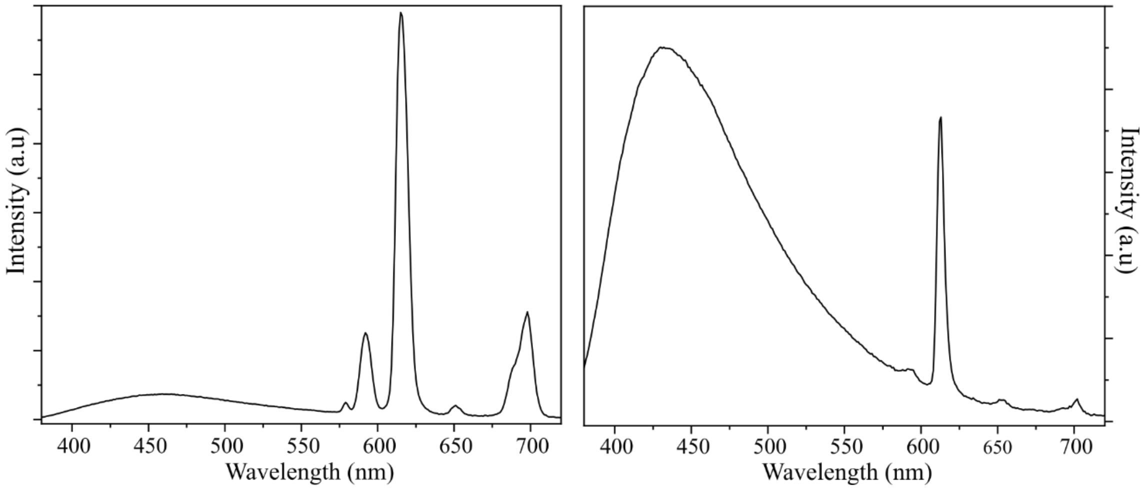

Figure S 45: Emission spectra of P_{66614}[Eu(BTFA)$_4$] solutions in acetonitrile (left) and chloroform (right) from an excitation wavelength of 370 nm after partial photobleaching under UVC and UVA light, respectively. The spectra illustrate the progressive photoreduction of Eu(III) to Eu(II) under UV illumination, as evidenced by the increasing relative intensity of the characteristic Eu(II) emission concurrent with the decreasing Eu(III) emission. The left spectrum exhibits less Eu(II) and more Eu(III), whereas the right spectrum shows the opposite trend, emphasizing the inverse relationship between the two emissions during photoreduction.

1.3 Luminescence time decay profiles

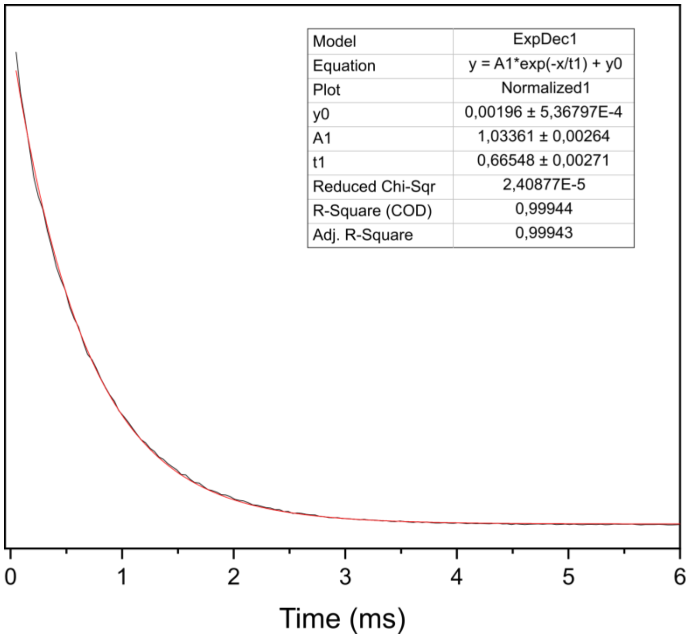

Figure S 46: Luminescence time decay profile of a 1x10^{-4} M solution of Li[Eu(BTFA)$_4$] complex in $CHCl_3$ at maximum excitation and emission wavelengths of λ= 361 nm and λ= 609 nm, respectively.

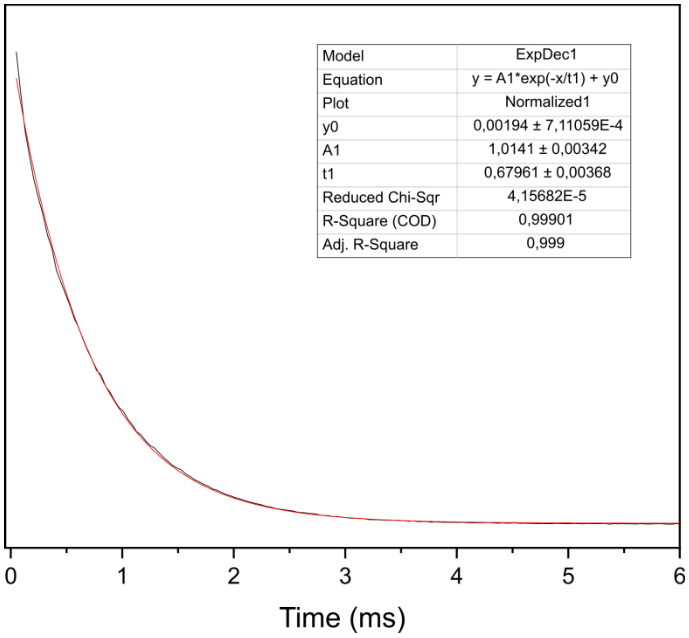

Figure S 47: Luminescence time decay profile of a 1x10^{-4} M solution of Li[Eu(BTFA)$_4$] complex, in CH_2Cl_2, at maximum excitation and emission wavelengths of 360 nm and 609 nm, respectively.

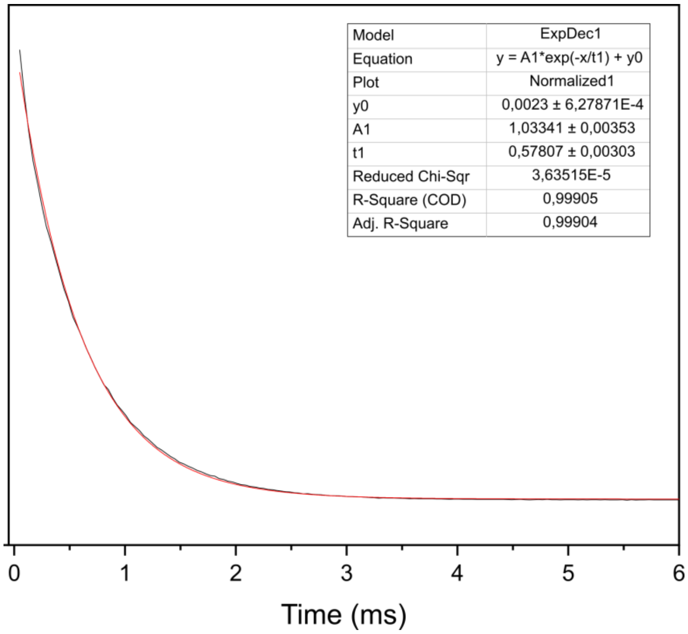

Figure S 48: Luminescence time decay profile of a 1x10^{-4} M solution of Li[Eu(BTFA)$_4$] complex in $(CH_3)_2CO$ at maximum excitation and emission wavelengths of λ= 361 nm and λ= 610 nm, respectively.

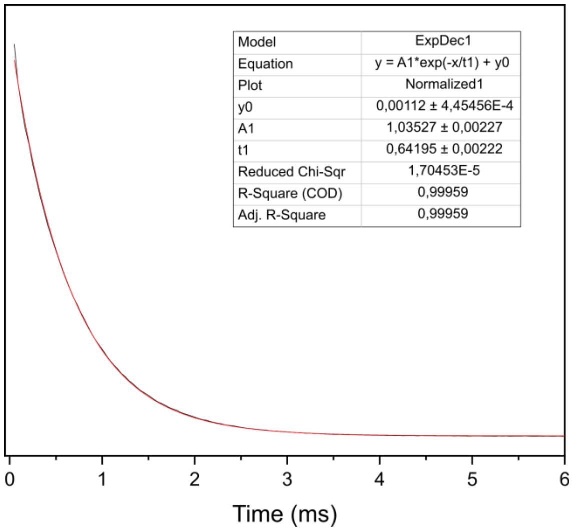

Figure S 49: Luminescence time decay profile of a 1x10^{-4} M solution of Li[Eu(BTFA)$_4$] complex in CH_3CN at maximum excitation and emission wavelengths of λ= 364 nm and λ= 610 nm, respectively.

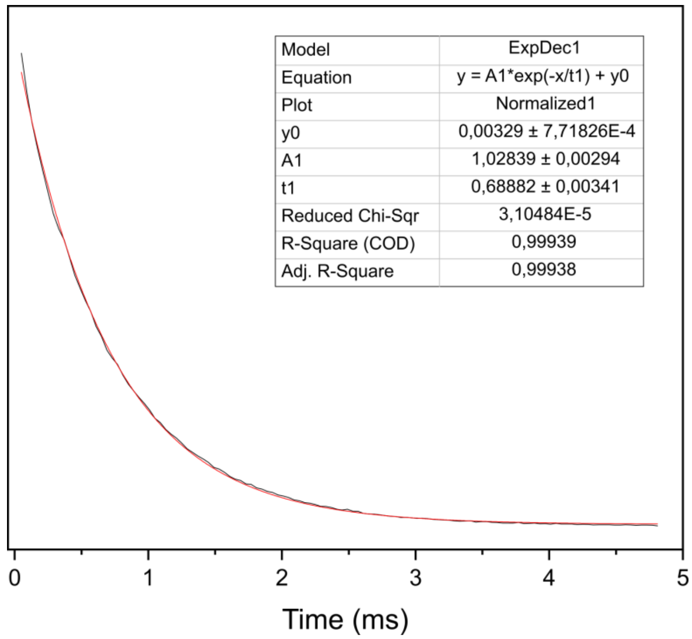

Figure S 50: Luminescence time decay profile of a 1x10^{-4} M solution of Na[Eu(BTFA)$_4$] complex in $CHCl_3$ at maximum excitation and emission wavelengths of λ= 365 nm and λ= 609 nm, respectively.

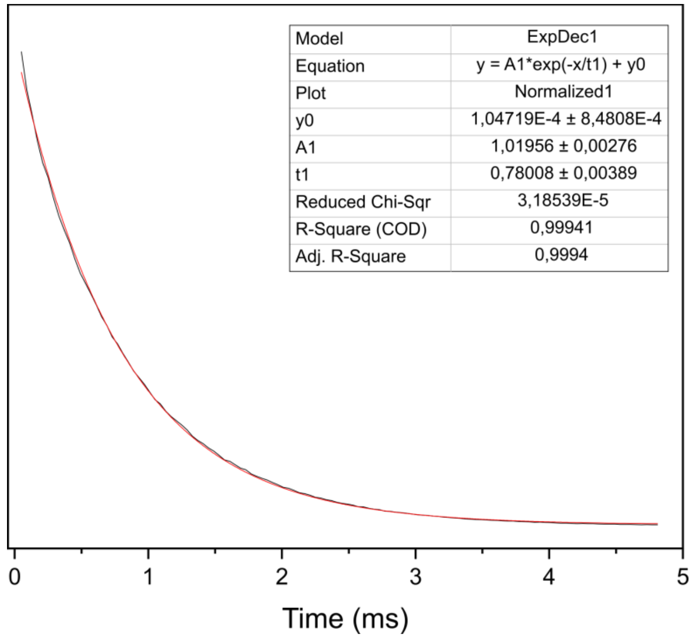

Figure S 51: Luminescence time decay profile of a 1x10^{-4} M solution of Na[Eu(BTFA)$_4$] in CH_2Cl_2 at maximum excitation and emission wavelengths of λ= 362 nm and λ= 609 nm, respectively.

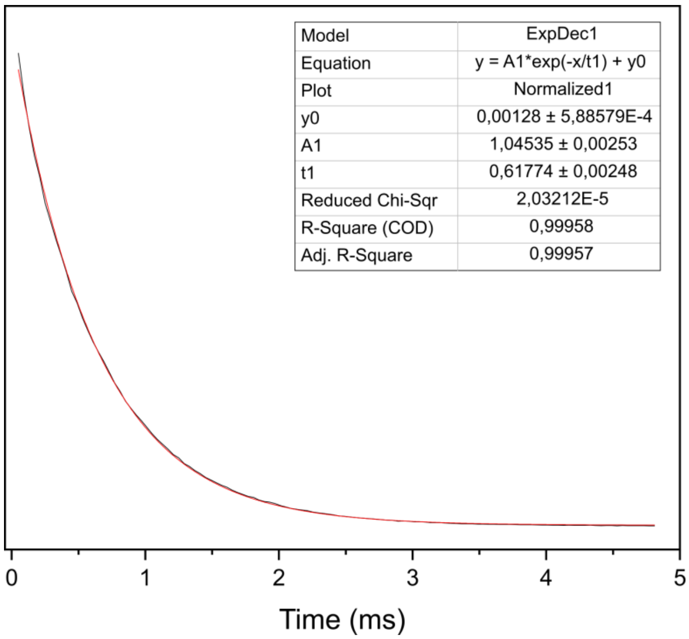

Figure S 52: Luminescence time decay profile of a 1x10^{-4} M solution of Na[Eu(BTFA)$_4$] complex in $(CH_3)_2CO$ at maximum excitation and emission wavelengths of λ= 368 nm and λ= 610 nm, respectively.

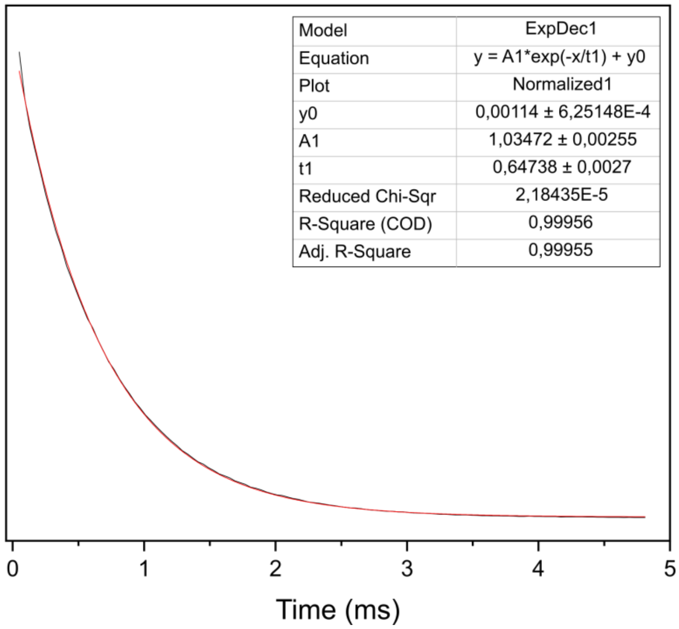

Figure S 53: Luminescence time decay profile of a 1x10^{-4} M solution of Na[Eu(BTFA)$_4$] complex in CH_3CN at maximum excitation and emission wavelengths of λ= 367 nm and λ= 610 nm, respectively.

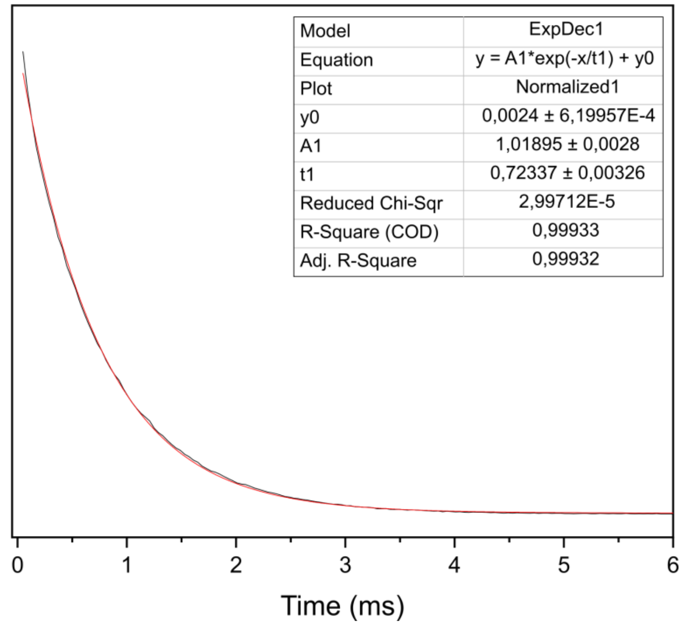

Figure S 54: Luminescence time decay profile of a 1x10^{-4} M solution of K[Eu(BTFA)$_4$] complex in $CHCl_3$ at maximum excitation and emission wavelengths of λ= 366 nm and λ= 609 nm, respectively.

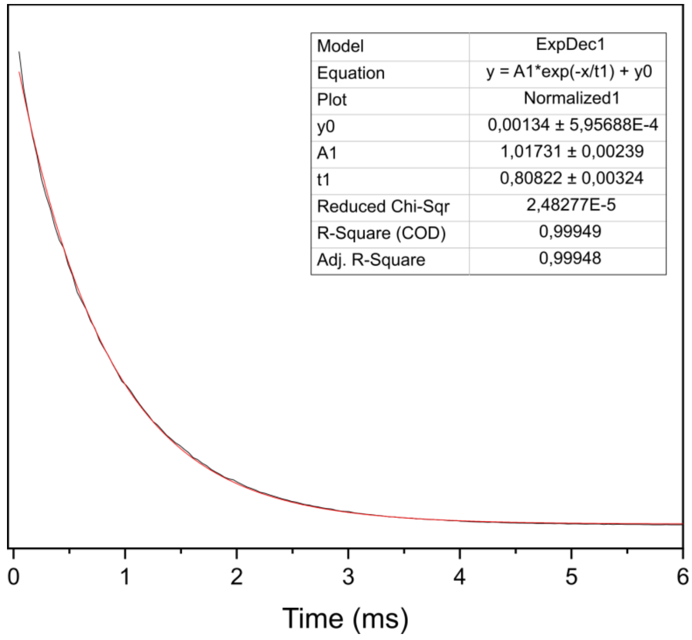

Figure S 55: Luminescence time decay profile of a 1x10^{-4} M solution of K[Eu(BTFA)$_4$] complex in CH_2Cl_2 at maximum excitation and emission wavelengths of λ= 365 nm and λ= 609 nm, respectively.

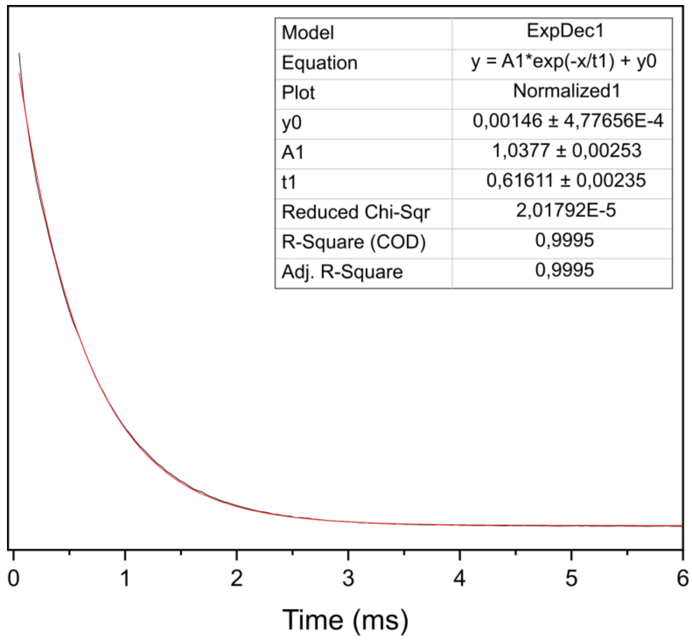

Figure S 56: Luminescence time decay profile of a 1x10^{-4} M solution of K[Eu(BTFA)$_4$] complex in $(CH_3)_2CO$ at maximum excitation and emission wavelengths of λ= 367 nm and λ= 610 nm, respectively.

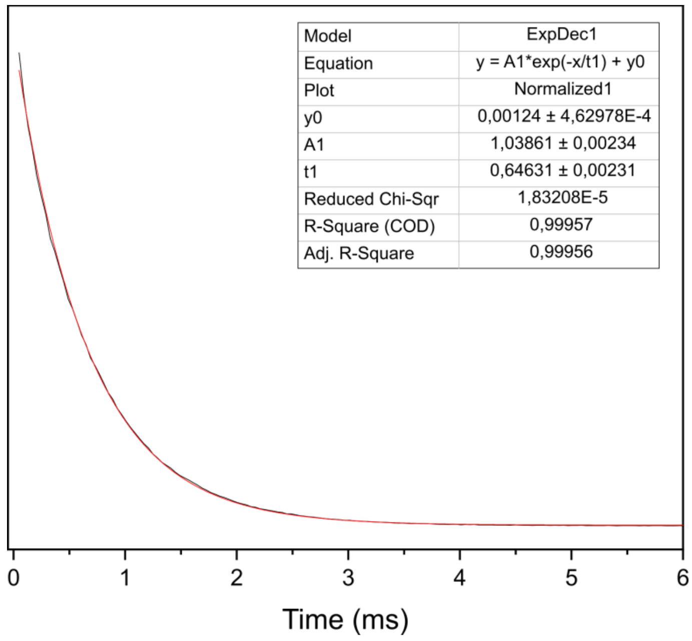

Figure S 57: Luminescence time decay profile of a 1x10^{-4} M solution of K[Eu(BTFA)$_4$] complex in CH_3CN at maximum excitation and emission wavelengths of λ= 368 nm and λ= 610 nm, respectively.

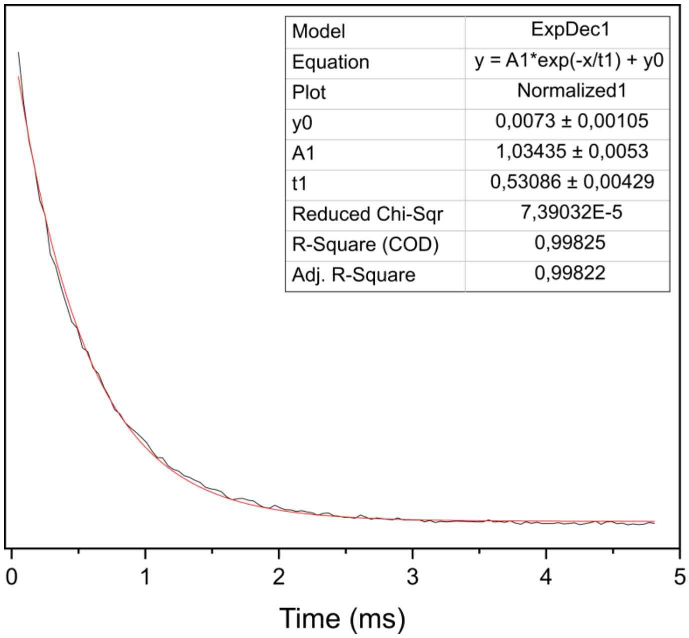

Figure S 58: Luminescence time decay profile of a 1x10^{-4} M solution of C_5mim[Eu(BTFA)$_4$] complex in $CHCl_3$ at maximum excitation and emission wavelengths of λ= 366 nm and λ= 611 nm, respectively.

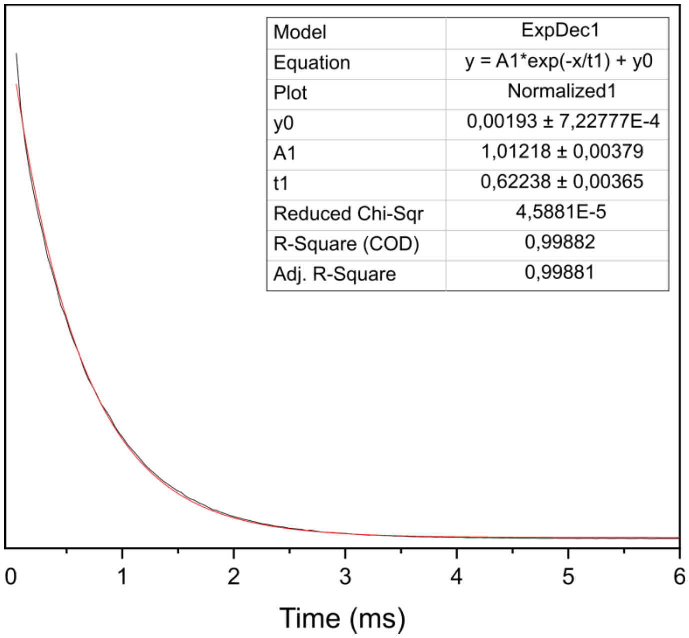

Figure S 59: Luminescence time decay profile of a 1x10^{-4} M solution of C_5mim[Eu(BTFA)$_4$] complex in CH_2Cl_2 at maximum excitation and emission wavelengths of λ= 366 nm and λ= 611 nm, respectively.

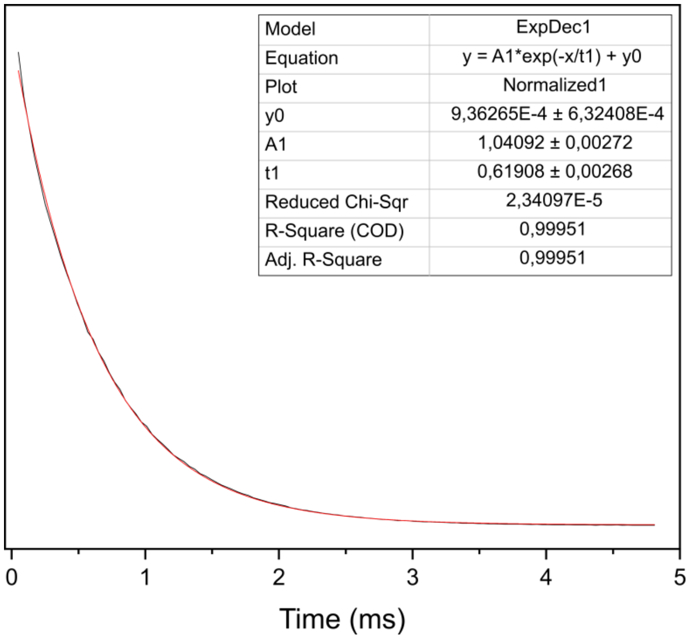

Figure S 60: Luminescence time decay profile of a $1x10^{-4}$ M solution of C_5mim[Eu(BTFA)$_4$] in $(CH_3)_2CO$ at maximum excitation and emission wavelengths of λ= 363 nm and λ= 611 nm, respectively.

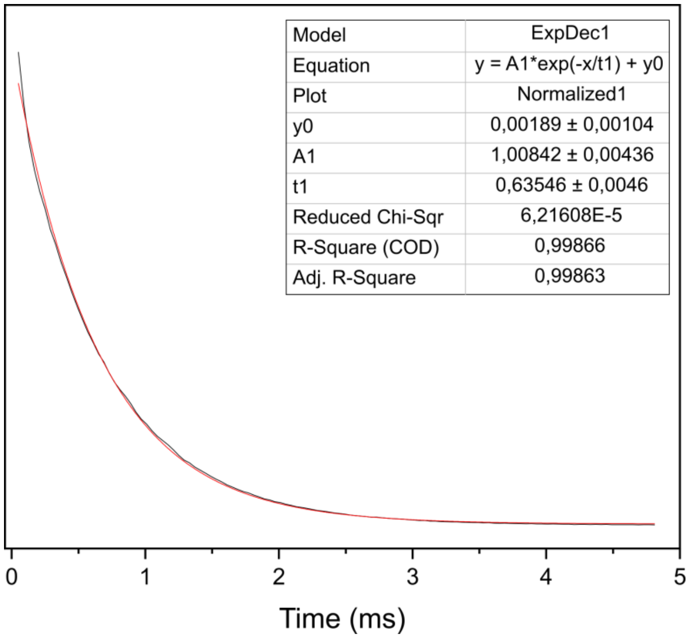

Figure S 61: Luminescence time decay profile of a $1x10^{-4}$ M solution of C_5mim[Eu(BTFA)$_4$] in CH_3CN at maximum excitation and emission wavelengths of λ= 366 nm and λ= 611 nm, respectively.

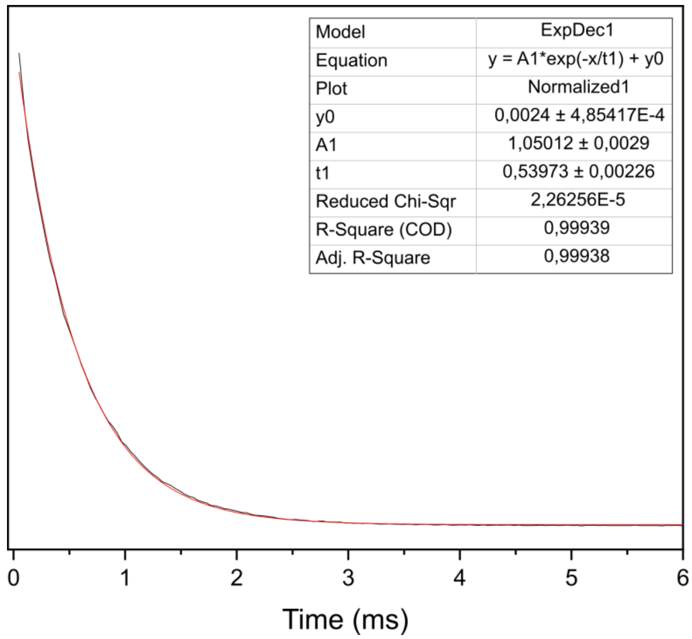

Figure S 62: Luminescence time decay profile of a 1x10^{-4} M solution of $P_{6,6,6,14}$]Eu(BTFA)$_4$] complex in $CHCl_3$ at maximum excitation and emission wavelengths of λ= 369 nm and λ= 610 nm, respectively.

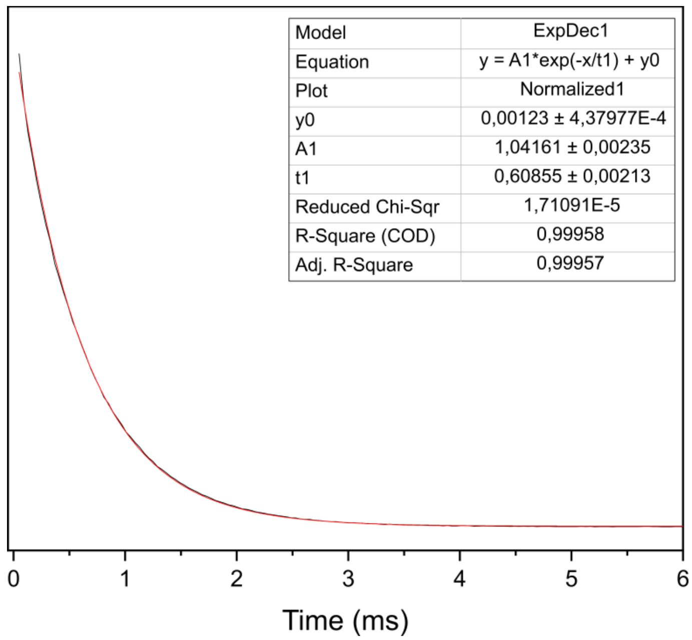

Figure S 63: Luminescence time decay profile of a 1x10^{-4} M solution of $P_{6,6,6,14}$[Eu(BTFA)$_4$] in CH_2Cl_2 at maximum excitation and emission wavelengths of λ= 368 nm and λ= 610 nm, respectively.

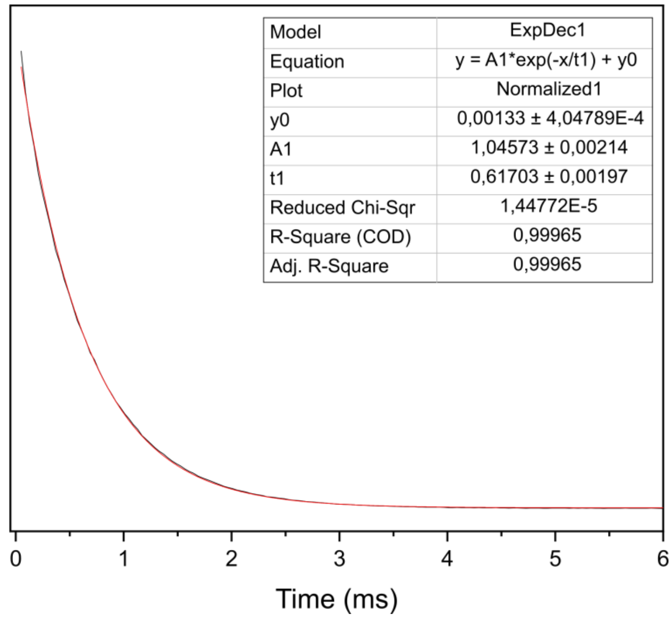

Figure S 64: Luminescence time decay profile of a 1x10^{-4} M solution of $P_{6,6,6,14}$[Eu(BTFA)$_4$] complex in $(CH_3)_2CO$ at maximum excitation and emission wavelengths of λ= 368 nm and λ= 610 nm, respectively.

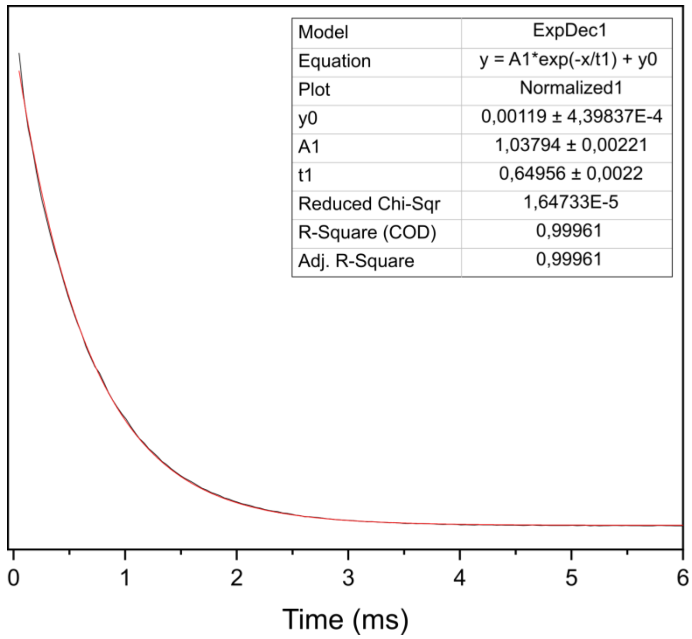

Figure S 65: Luminescence time decay profile of a 1x10^{-4} M solution of $P_{6,6,6,14}$[Eu(BTFA)$_4$] in CH_3CN at maximum excitation and emission wavelengths of λ= 367 nm and λ= 610 nm, respectively.

Table S 1. Luminescence data for all 1x10^{-4} M solution of $[Eu(BTFA)_4]^-$ complexes in the four solvents studied. Lifetimes, τ; radiative decay rates, A_{rad}; nonradiative decay rates, A_{nrad}; quantum efficiency, η; luminescence data experimental errors within a 90% confidence interval are: τ_{obs}, 4% ; A^{rad} 3% ; A_{nrad} 8% ; η, 5%; of the reported values.

$[Eu(BTFA)]^-$	Parameters	Chloroform	Dichloromethane	Acetone	Acetonitrile
**Li^+**	τ (ms)	0.668	0.678	0.581	0.636
	A_{rad} (s^{-1})	797.77	743.66	742.54	640.46
	A_{nrad} (s^{-1})	699.42	731.00	979.54	931.15
	***η* (%)**	**53.28**	**50.43**	**43.12**	**40.75**
**Na+**	τ (ms)	0.684	0.776	0.617	0.640
	A_{rad} (s^{-1})	769.55	690.07	676.95	636.19
	A_{nrad} (s^{-1})	698.95	599.20	942.89	925.70
	***η* (%)**	**52.43**	**53.53**	**41.79**	**40.74**
**K^+**	τ (ms)	0.708	0.749	0.617	0.642
	A_{rad} (s^{-1})	703.68	693.70	673.34	625.90
	A_{nrad} (s^{-1})	709.30	672.95	946.96	932.58
	***η* (%)**	**49.79**	**50.77**	**41.56**	**40.16**
**C_5mim^+**	τ (ms)	0.531	0.622	0.619	0.635
	A_{rad} (s^{-1})	1040.83	717.44	631.71	597.77
	A_{nrad} (s^{-1})	843.12	889.51	983.80	976.04
	***η* (%)**	**55.25**	**44.65**	**39.10**	**37.98**
**$P_{6,6,6,14}^+$**	τ (ms)	0.533	0.604	0.613	0.648
	A_{rad} (s^{-1})	853.79	704.17	642.24	618.40
	A_{nrad} (s^{-1})	1056.15	951.83	989.95	925.86
	***η* (%)**	**45.52**	**42.52**	**39.35**	**40.05**

2.Cyclic voltammograms

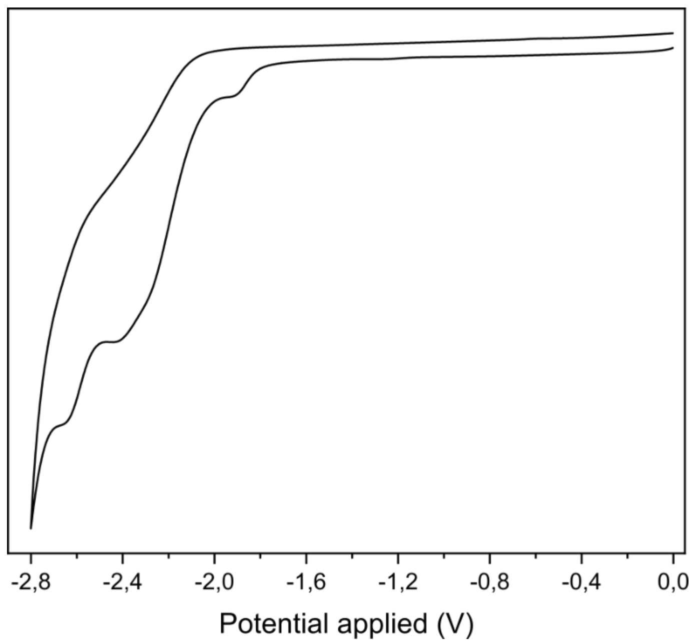

Figure S 66: Cyclic voltammograms from -2,8 to 0 V of the 10 mmol·L^{-1} KBTFA in acetonitrile/0.1 mol·L^{-1} $TBABF_4$, v = 100 mV·s^{-1}, using Ag/AgCl, 3.0 mol·L^{-1} KCl as reference electrode.

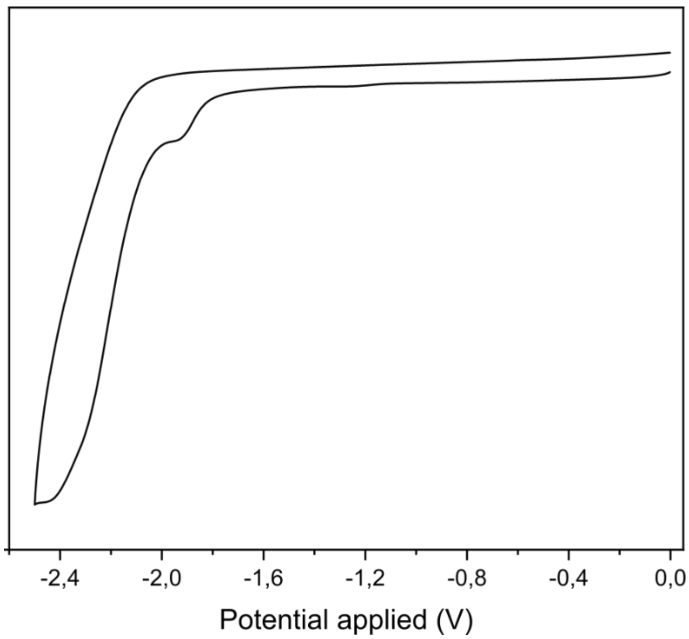

Figure S 67: Cyclic voltammograms from -2,5 to 0 V of the 10 mmol·L^{-1} KBTFA in acetonitrile/0.1 mol·L^{-1} $TBABF_4$, v = 100 mV·s^{-1}, using Ag/AgCl, 3.0 mol·L^{-1} KCl as reference electrode.

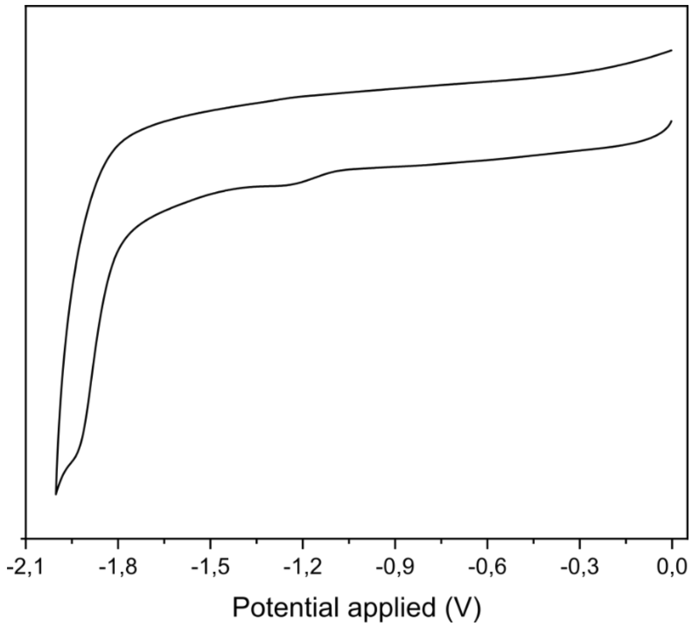

Figure S 68: Cyclic voltammograms from -2.0 to 0 V of the 10 mmol·L^{-1} KBTFA in acetonitrile/0.1 mol·L^{-1} $TBABF_4$, v = 100 mV·s^{-1}, using Ag/AgCl, 3.0 mol·L^{-1} KCl as reference electrode.

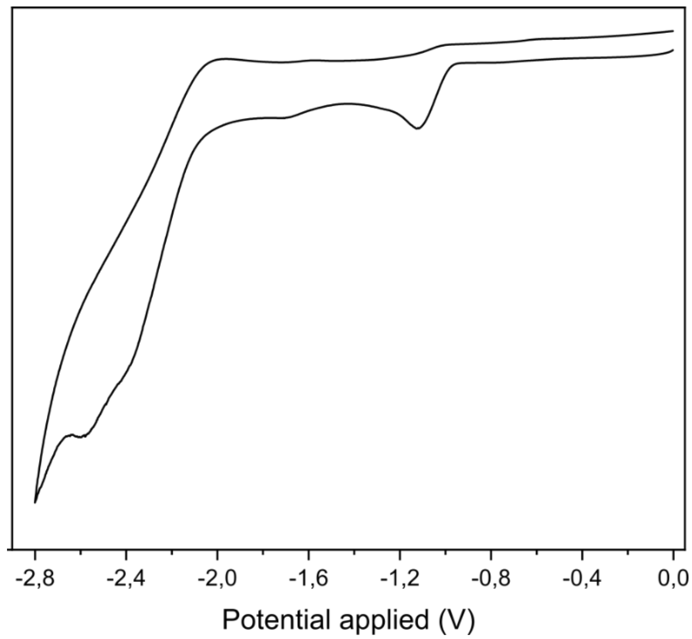

Figure S 69: Cyclic voltammograms from -2.8 to 0 V of the 10 mmol·L^{-1} HBTFA in acetonitrile/0.1 mol·L^{-1} $TBABF_4$, v = 100 mV·s^{-1}, using Ag/AgCl, 3.0 mol·L^{-1} KCl as reference electrode.

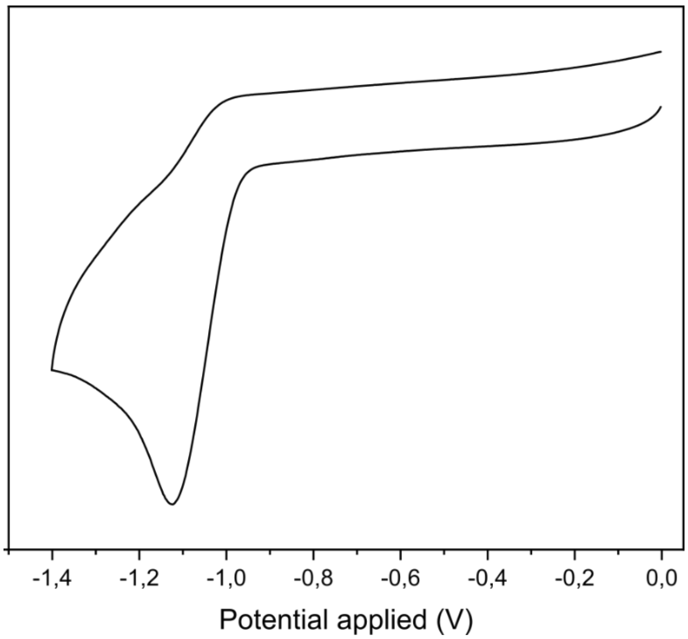

Figure S 70: Cyclic voltammograms from -1.4 to 0 V of the 10 mmol·L^{-1} HBTFA in acetonitrile/0.1 mol·L^{-1} $TBABF_4$, v = 100 mV·s^{-1}, using Ag/AgCl, 3.0 mol·L^{-1} KCl as reference electrode.

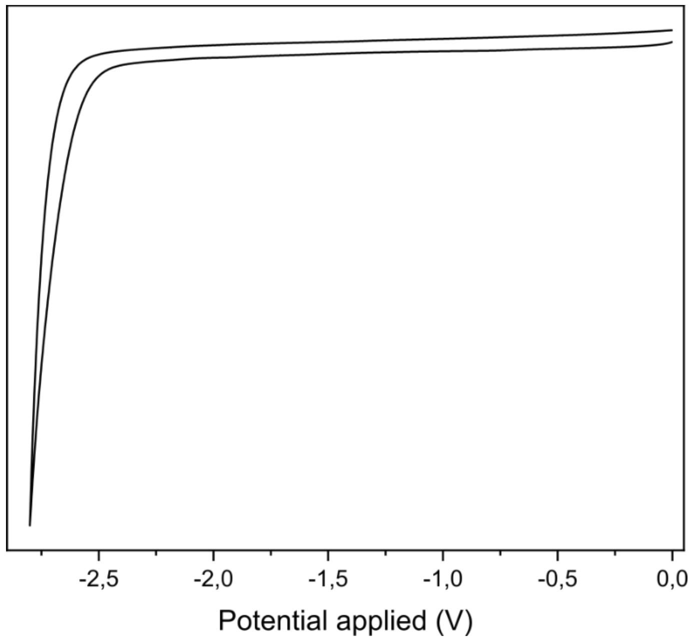

Figure S 71: Cyclic voltammograms from -2.8 to 0 V of the 10 mmol·L^{-1} $P_{6,6,6,14}Cl$ in acetonitrile/0.1 mol·L^{-1} $TBABF_4$, v = 100 mV·s^{-1}, using Ag/AgCl, 3.0 mol·L^{-1} KCl as reference electrode.

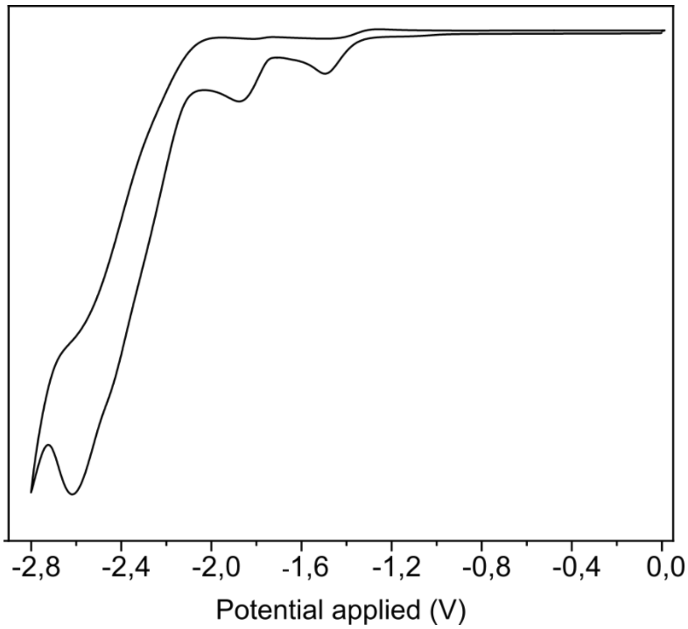

Figure S 72: Cyclic voltammograms from -2,8 to 0 V of the 10 mmol·L^{-1} $P_{6,6,6,14}[Eu^{III}(BTFA)_4]$ in acetonitrile/0.1 mol·L^{-1} $TBABF_4$, v = 100 mV·s^{-1}, using Ag/AgCl, 3.0 mol·L^{-1} KCl as reference electrode.

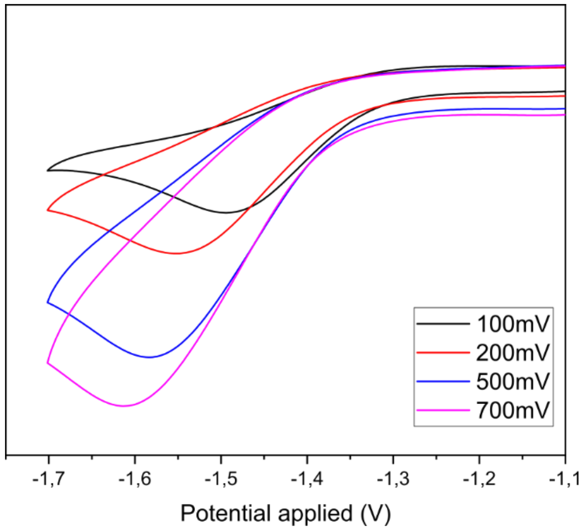

Figure S 73: Cyclic voltammograms from -1.7 to -1.1 V of the 10 mmol·L^{-1} $P_{6,6,6,14}[Eu^{III}(BTFA)_4]$ in acetonitrile/0.1 mol·L^{-1} $TBABF_4$, v = 100 mV·s^{-1}, using Ag/AgCl, 3.0 mol·L^{-1} KCl as reference electrode. The experiment was performed at the following different scan rates: 100mV/s (black line), 200mV/s (red line), 300mV/s (blue line), 700mV/s (purple line). No oxidation peaks were observed after scan reversion indicating irreversible behavior.

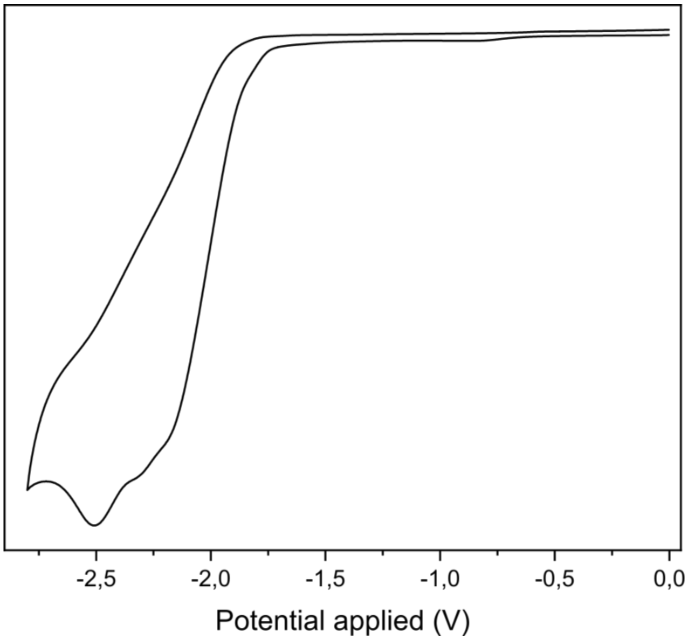

Figure S 74: Cyclic voltammograms from -2.8 to 0 V of the 10 mmol·L^{-1} $P_{6,6,6,14}[Eu^{II}(BTFA)_4]$ after electrolysis in acetonitrile/0.1 mol·L^{-1} $TBABF_4$, v = 100 mV·s^{-1}, using Ag/AgCl, 3.0 mol·L^{-1} KCl as reference electrode.

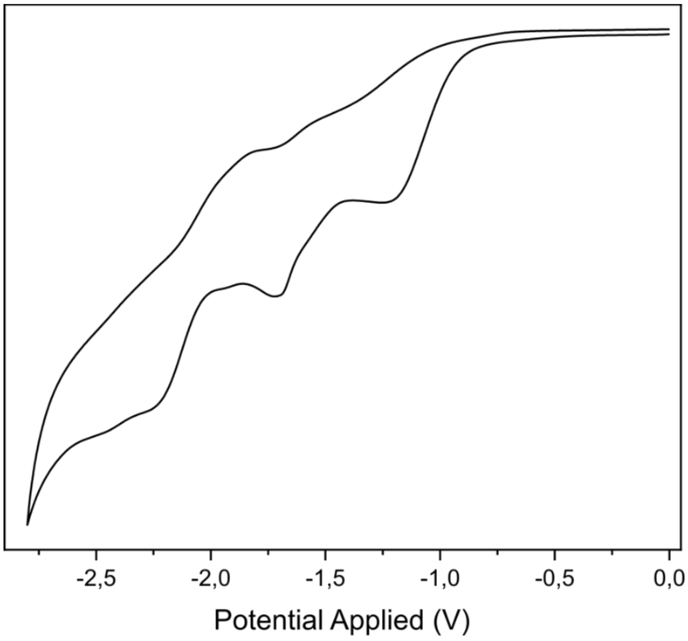

Figure S 75: Cyclic voltammograms from -2.8 to 0 V of the 10 mmol·L^{-1} $P_{6,6,6,14}[Eu^{II}(BTFA)_4]$ after UV irradiation in acetonitrile/0.1 mol·L^{-1} $TBABF_4$, v = 100 mV·s^{-1}, using Ag/AgCl, 3.0 mol·L^{-1} KCl as reference electrode.

3. Photostability

3.1. Polynomial adjustments of the emission intensity as a function of the UVA energy

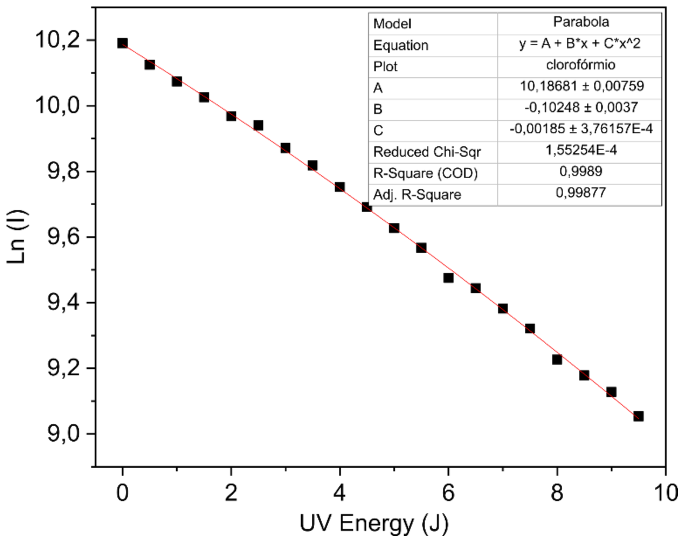

Figure S 76: Polynomial fit ln(I) = a + bE + CE^2 of the $1x10^{-4}$ M solution of Li[Eu(BTFA)$_4$] complex in $CHCl_3$ as a function of the UVA energy.

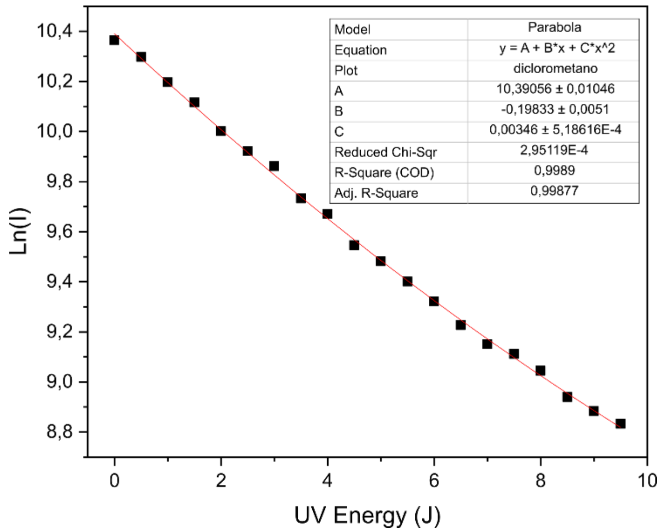

Figure S 77: Polynomial fit ln(I) = a + bE + CE^2 of the $1x10^{-4}$ M solution of Li[Eu(BTFA)$_4$] complex in CH_2Cl_2 as a function of UVA energy.

.

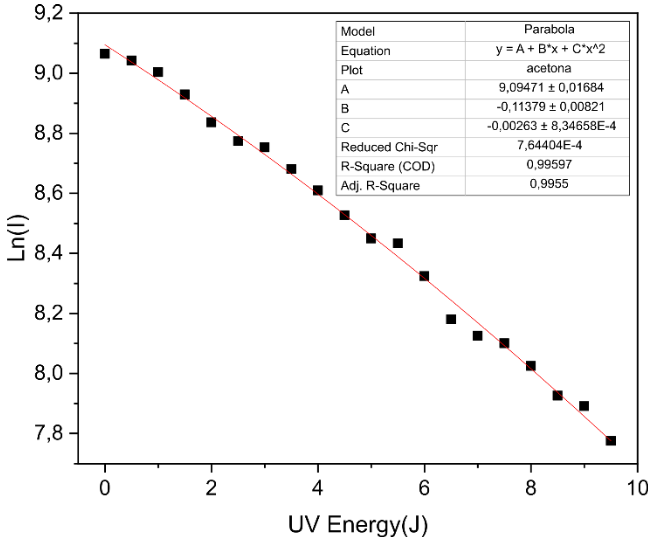

Figure S 78: Polynomial fit ln(I) = a + bE + CE^2 of the $1x10^{-4}$ M solution of $Li[Eu(BTFA)_4]$ complex in $(CH_3)_2CO$ as a function of UVA energy.

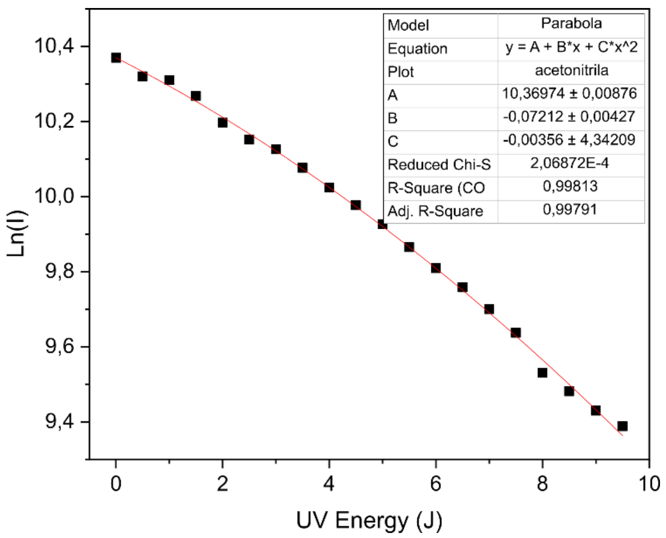

Figure S 79: Polynomial fit ln(I) = a + bE + CE^2 of the $1x10^{-4}$ M solution of $Li[Eu(BTFA)_4]$ complex in CH_3CN as a function of UVA energy.

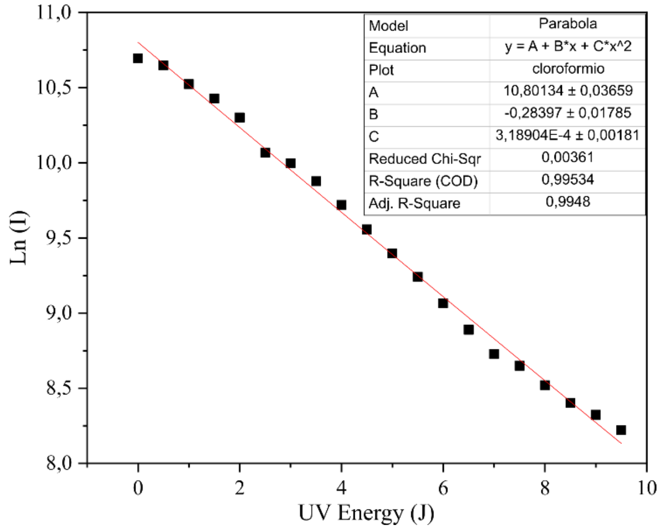

Figure S 80: Polynomial fit ln(I) = a + bE + CE^2 of the 1x10^{-4} M solution of Na[Eu(BTFA)$_4$] complex in $CHCl_3$ as a function of the UVA energy.

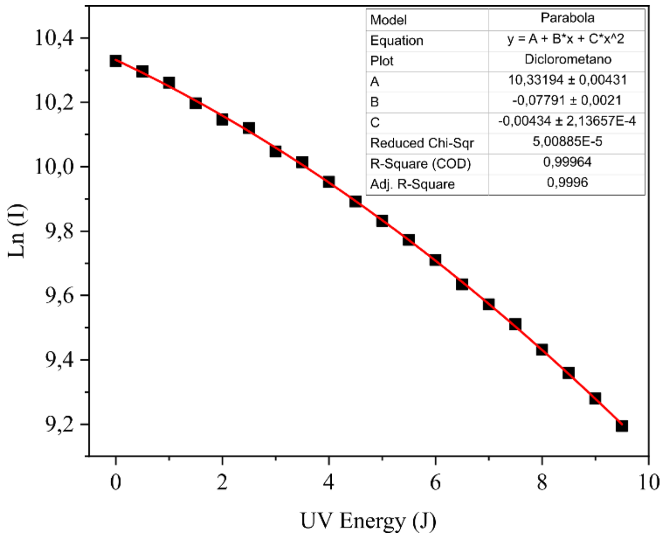

Figure S 81: Polynomial fit ln(I) = a + bE + CE^2 of the 1x10^{-4} M solution of Na[Eu(BTFA)$_4$] complex in CH_2Cl_2 as a function of UVA energy.

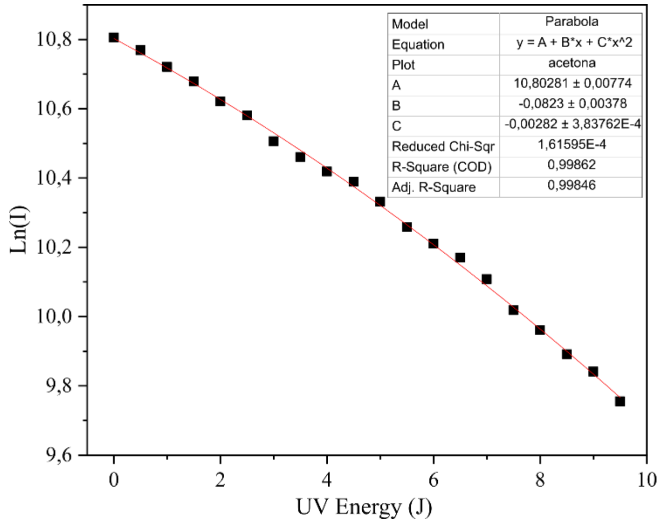

Figure S 82: Polynomial fit ln(I) = a + bE + CE^2 of the 1x10^{-4} M solution of Na[Eu(BTFA)$_4$] complex in $(CH_3)_2CO$ as a function of UVA energy.

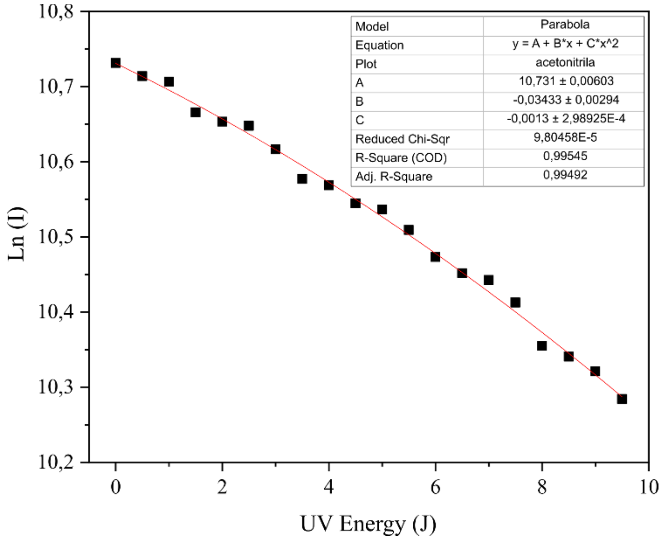

Figure S 83: Polynomial fit ln(I) = a + bE + CE^2 of the 1x10^{-4} M solution of Na[Eu(BTFA)$_4$] complex in CH_3CN as a function of UVA energy.

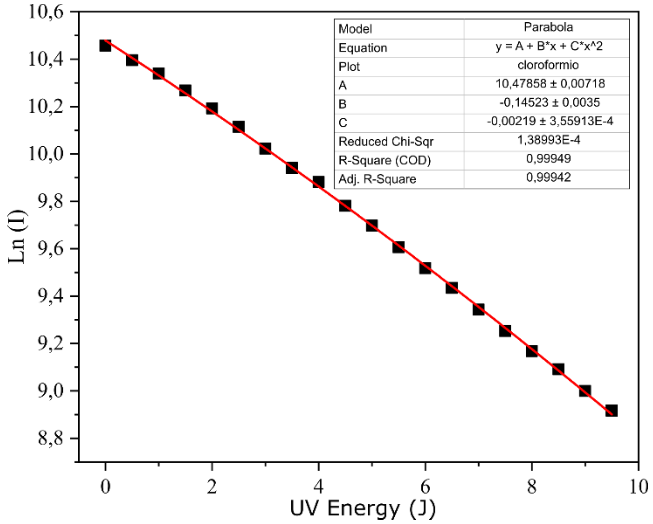

Figure S 84: Polynomial fit ln(I) = a + bE + CE^2 of the $1x10^{-4}$ M solution of K[Eu(BTFA)$_4$] complex in $CHCl_3$ as a function of the UVA energy.

.

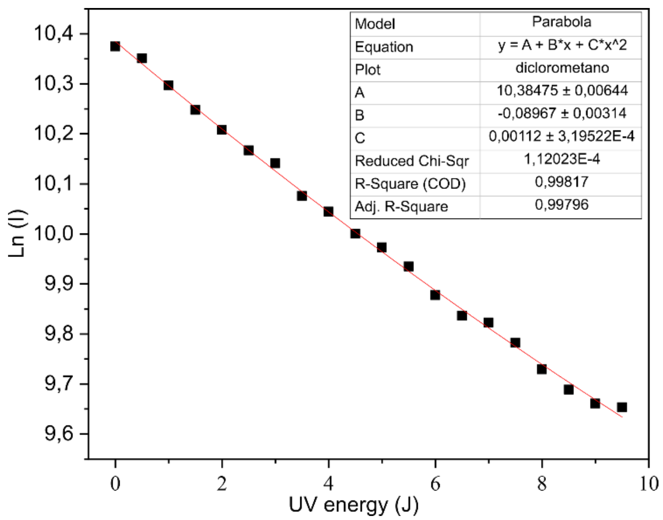

Figure S 85: Polynomial fit ln(I) = a + bE + CE^2 of the $1x10^{-4}$ M solution of K[Eu(BTFA)$_4$] complex in CH_2Cl_2 as a function of UVA energy.

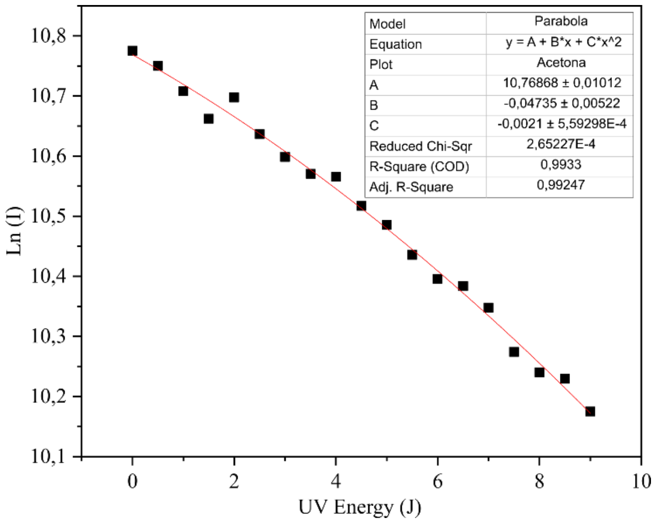

Figure S 86: Polynomial fit ln(I) = a + bE + CE^2 of the $1x10^{-4}$ M solution of K[Eu(BTFA)$_4$] complex in $(CH_3)_2CO$ as a function of UVA energy.

.

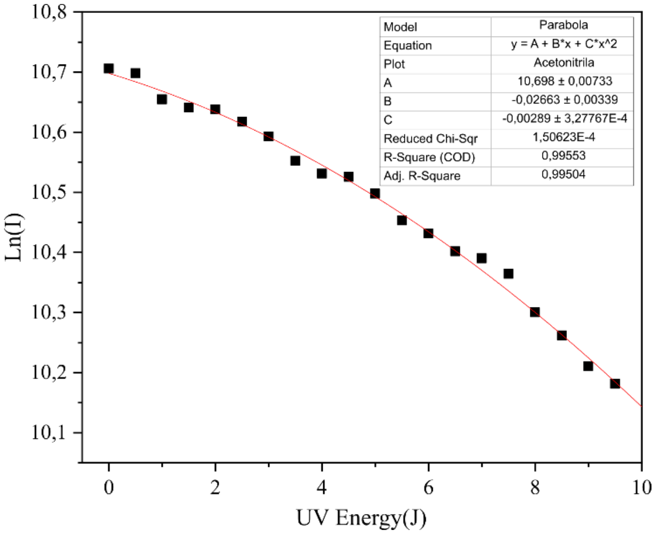

Figure S 87: Polynomial fit ln(I) = a + bE + CE^2 of the $1x10^{-4}$ M solution of K[Eu(BTFA)$_4$] complex in CH_3CN as a function of UVA energy.

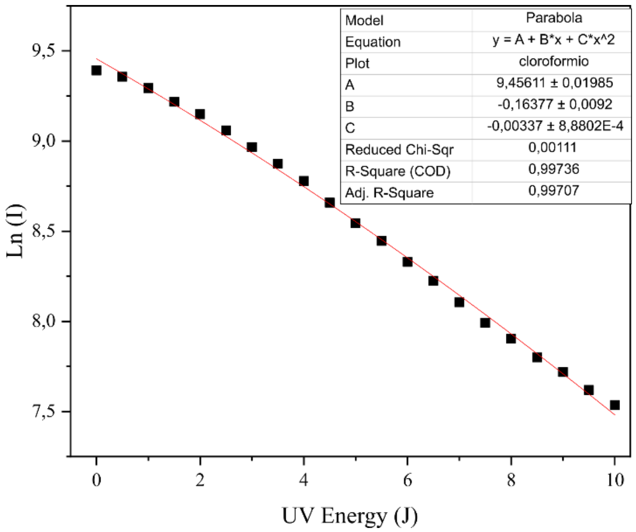

Figure S 88: Polynomial fit ln(I) = a + bE + CE^2 of the $1x10^{-4}$ M solution of C_5mim[Eu(BTFA)$_4$] in $CHCl_3$ as a function of UVA energy.

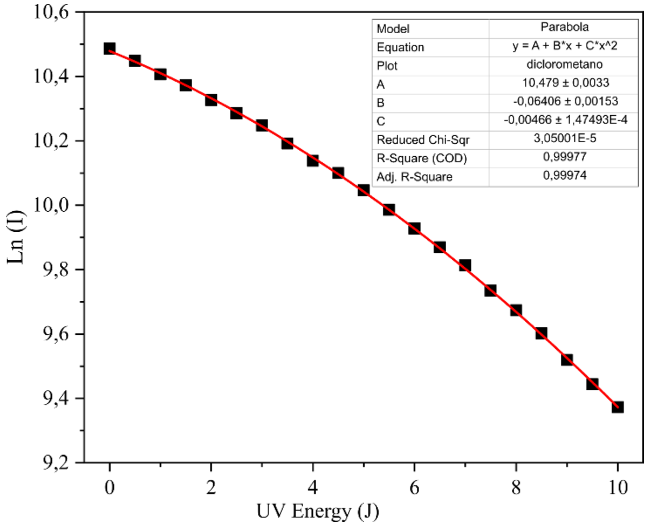

Figure S 89: Polynomial fit ln(I) = a + bE + CE^2 of the $1x10^{-4}$ M solution of C_5mim[Eu(BTFA)$_4$] complex in CH_2Cl_2 as a function of UVA energy.

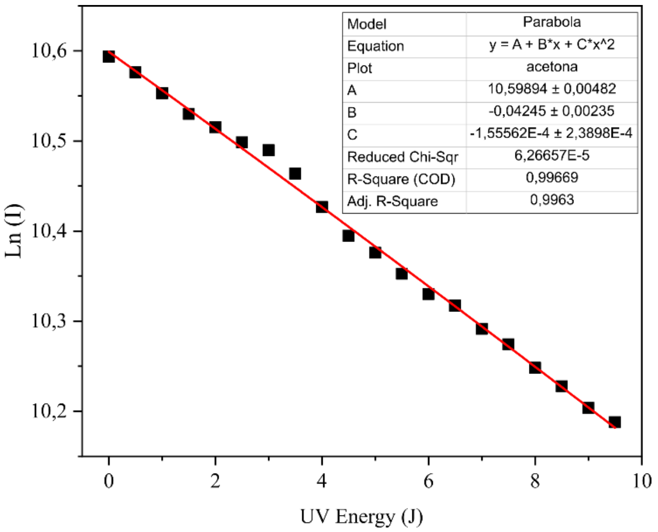

Figure S 90: Polynomial fit ln(I) = a + bE + CE^2 of the $1x10^{-4}$ M solution of C_5mim[Eu(BTFA)$_4$] complex in $(CH_3)_2CO$ as a function of UVA energy..

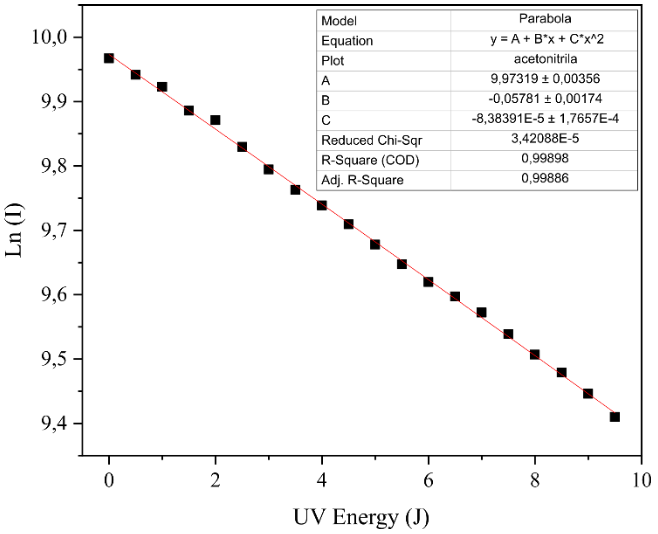

Figure S 91: Polynomial fit ln(I) = a + bE + CE^2 of the $1x10^{-4}$ M solution of C_5mim[Eu(BTFA)$_4$] complex in CH_3CN as a function of UVA energy.

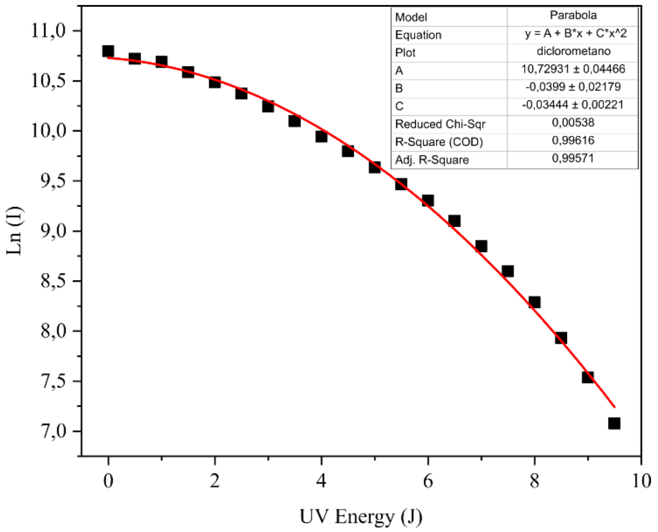

Figure S 92: Polynomial fit ln(I) = a + bE + CE^2 of the 1x10^{-4} M solution of $P_{6,6,6,14}$[Eu(BTFA)$_4$] complex in CH_2Cl_2 as a function of UVA energy..

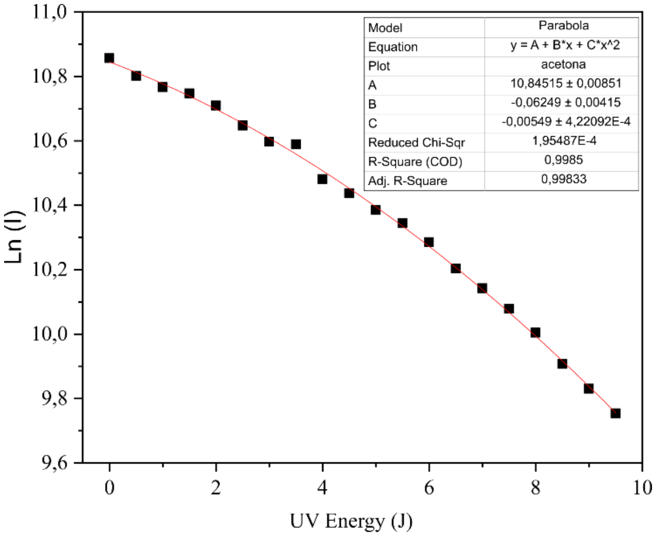

Figure S 93: Polynomial fit ln(I) = a + bE + CE^2 of the 1x10^{-4} M solution of $P_{6,6,6,14}$[Eu(BTFA)$_4$] complex in $(CH_3)_2CO$ as a function of UVA energy.

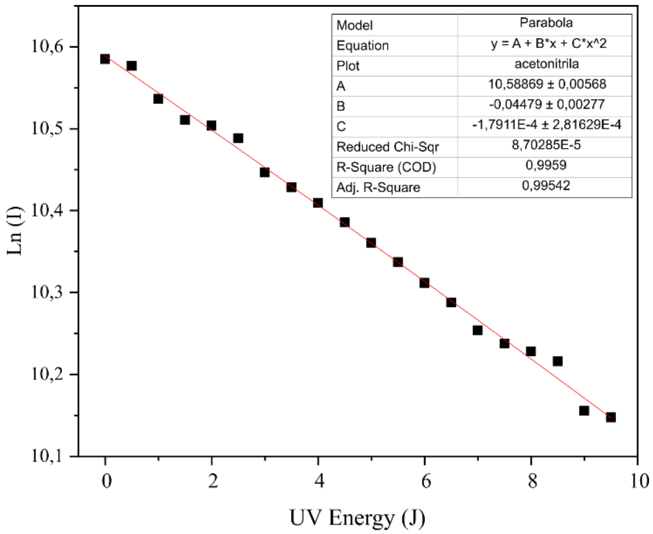

Figure S 94: Polynomial fit ln(I) = a + bE + CE^2 of the 1x10^{-4} M solution of $P_{6,6,6,14}$[Eu(BTFA)$_4$] complex in CH_3CN as a function of UVA energy.

4. NMR Data

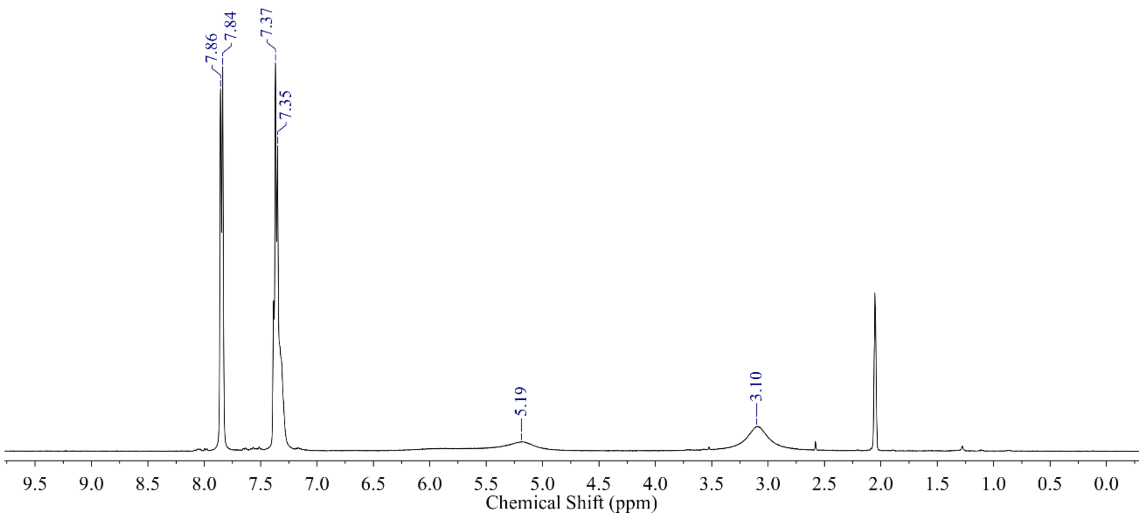

Figure S 95: ^{1}H NMR spectrum of K[Eu(BTFA)$_4$] complex acquired using a 400 MHz spectrometer in acetone-d.

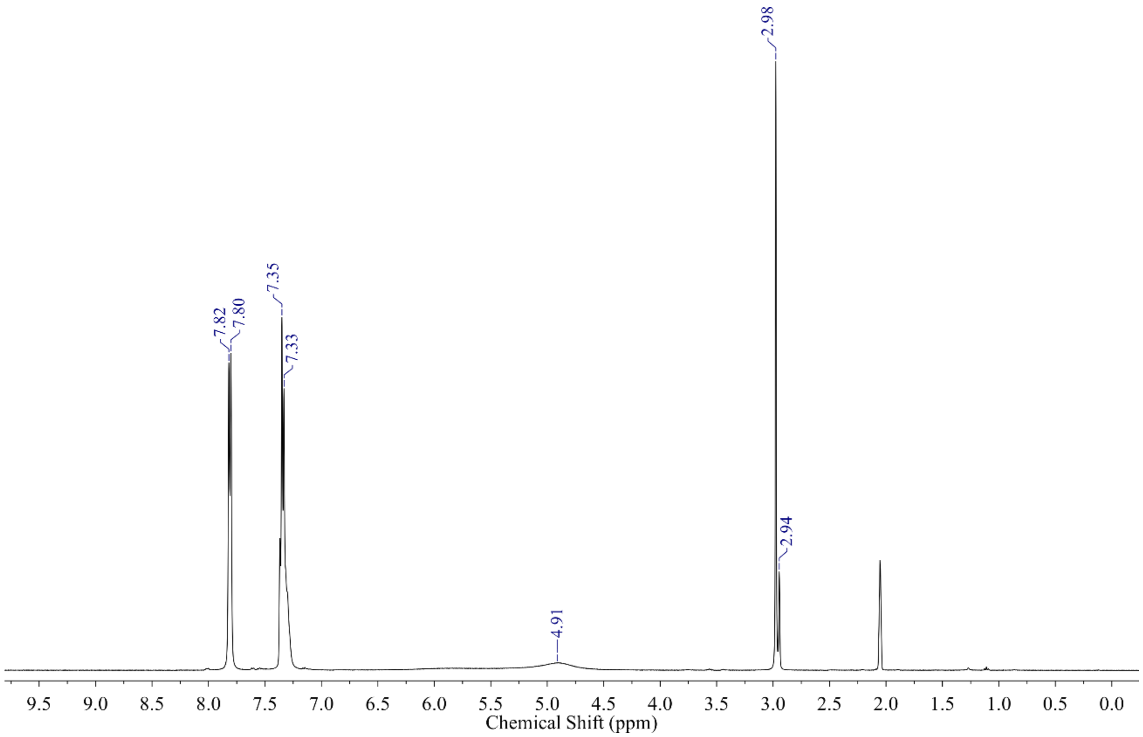

Figure S 96: ^{1}H NMR spectrum of the Na[Eu(BTFA)$_4$] complex. Acquired on a 400 MHz spectrometer in Acetone-d.

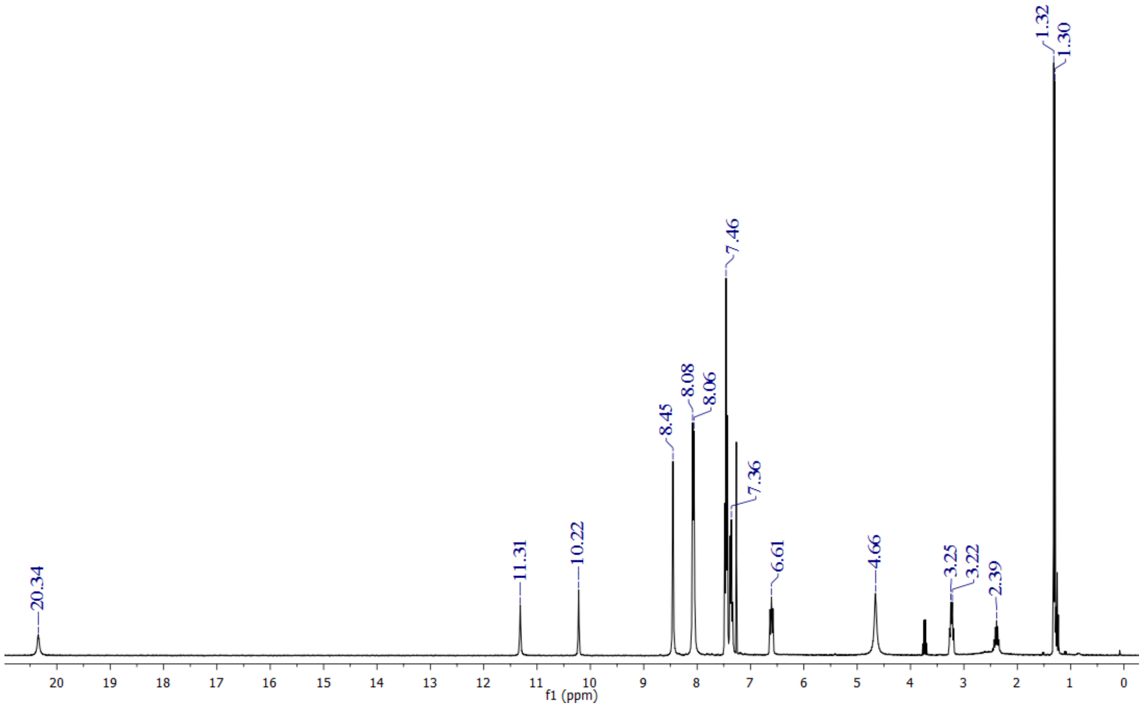

Figure S 97: ^{1}H NMR spectrum of C_5mim[Eu(BTFA)$_4$] complex. Acquired on a 400 MHz spectrometer in chloroform-d.

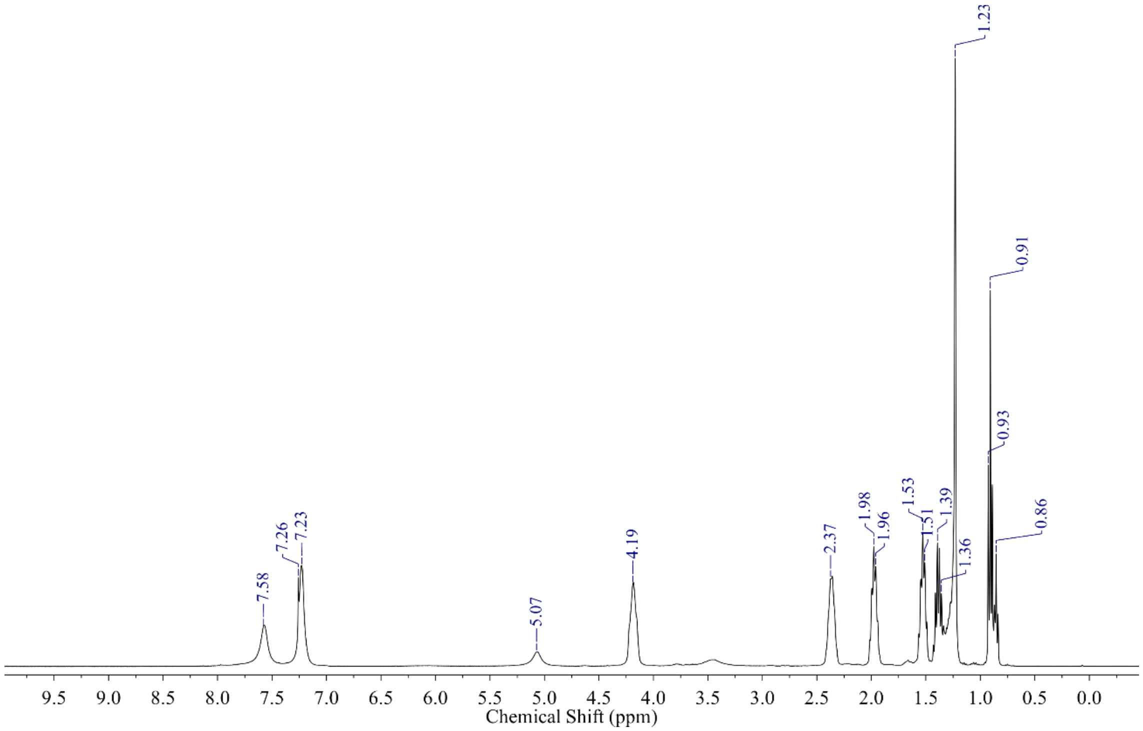

Figure S 98: ^{1}H NMR spectrum of the $P_{6,6,6,14}$[Eu(BTFA)$_4$] complex. Acquired on a 400 MHz spectrometer in chloroform-d.

5. Infrared Spectra

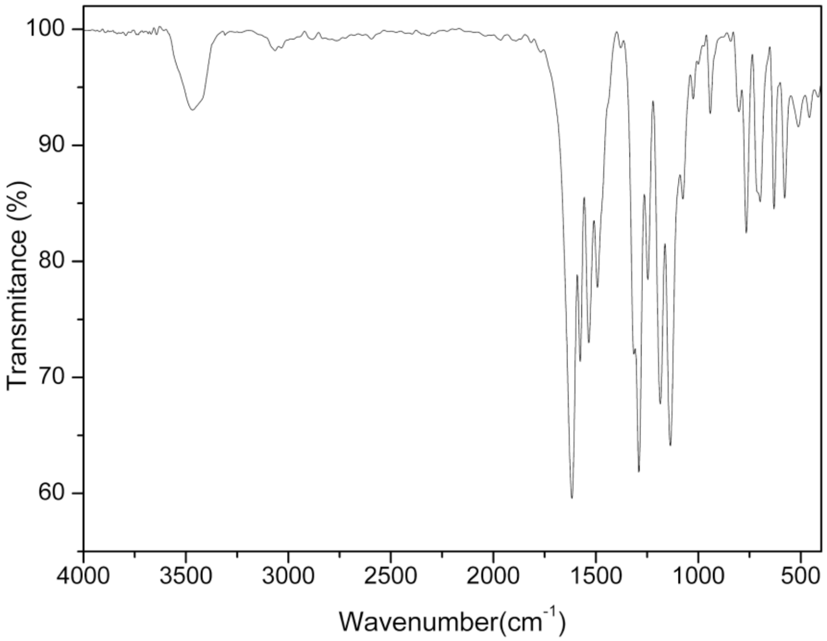

Figure S 99: Infrared spectrum of $K[Eu(BTFA)_4]$ complex. Acquired in KBr disk.

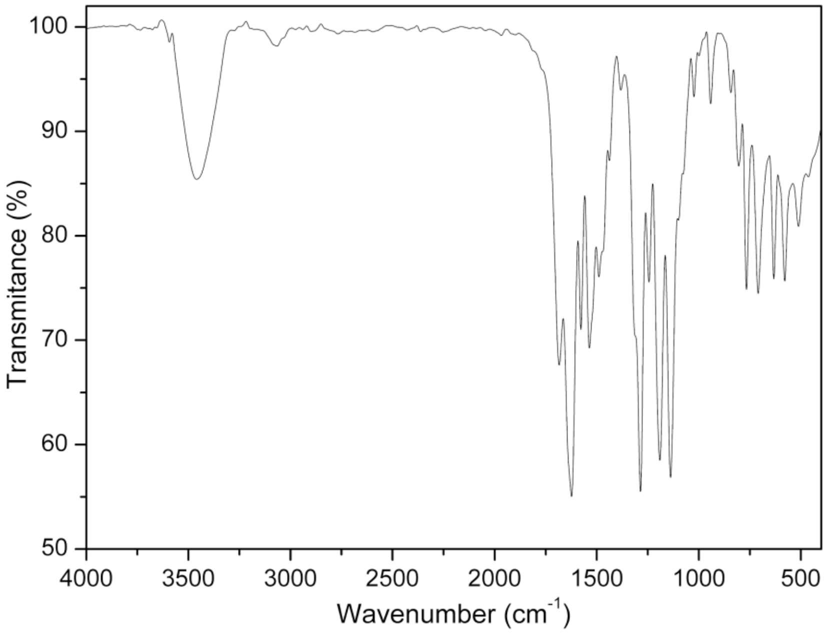

Figure S 100: Infrared spectrum of $Na[Eu(BTFA)_4]$ complex. Acquired in KBr disk.

.

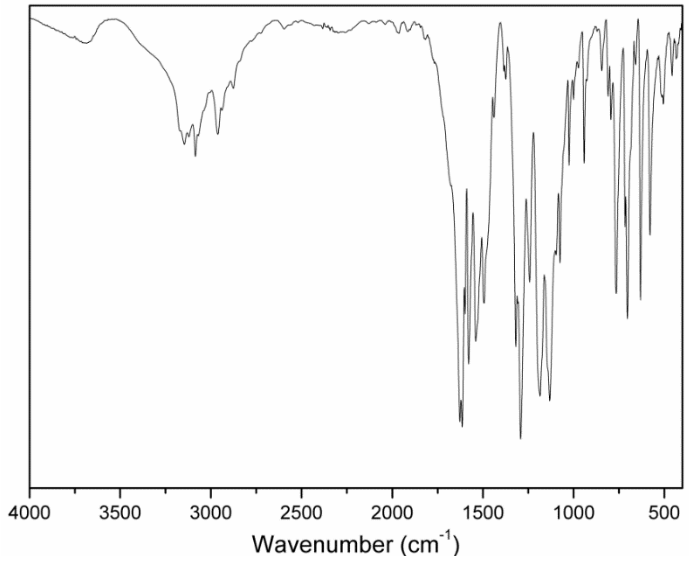

Figure S 101: Infrared spectrum of C_5mim[Eu(BTFA)$_4$] complex. Acquired in KBr disk.

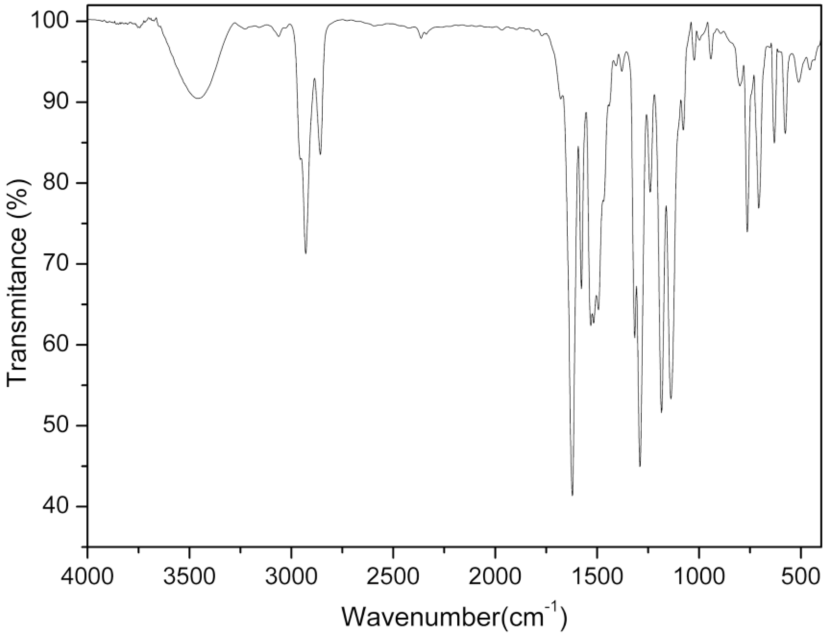

Figure S 102: Infrared spectrum of $P_{6,6,6,14}$[Eu(BTFA)$_4$] complex. Acquired in KBr disk.
